# Capture of activated dioxygen intermediates at the copper-active site of a lytic polysaccharide monooxygenase†

**DOI:** 10.1039/d2sc05031e

**Published:** 2022-11-02

**Authors:** Gabriela C. Schröder, William B. O'Dell, Simon P. Webb, Pratul K. Agarwal, Flora Meilleur

**Affiliations:** Department of Molecular and Structural Biochemistry, North Carolina State University Raleigh NC 27695 USA fmeille@ncsu.edu; Neutron Scattering Division, Oak Ridge National Laboratory Oak Ridge TN 37831 USA; VeraChem LLC 12850 Middlebrook Rd. Ste 205 Germantown MD 20874-5244 USA; Department of Physiological Sciences and High-Performance Computing Center, Oklahoma State University Stillwater OK 74078 USA pratul.agarwal@okstate.edu

## Abstract

Metalloproteins perform a diverse array of redox-related reactions facilitated by the increased chemical functionality afforded by their metallocofactors. Lytic polysaccharide monooxygenases (LPMOs) are a class of copper-dependent enzymes that are responsible for the breakdown of recalcitrant polysaccharides *via* oxidative cleavage at the glycosidic bond. The activated copper-oxygen intermediates and their mechanism of formation remains to be established. Neutron protein crystallography which permits direct visualization of protonation states was used to investigate the initial steps of oxygen activation directly following active site copper reduction in *Neurospora crassa* LPMO9D. Herein, we cryo-trap an activated dioxygen intermediate in a mixture of superoxo and hydroperoxo states, and we identify the conserved second coordination shell residue His157 as the proton donor. Density functional theory calculations indicate that both superoxo and hydroperoxo active site states are stable. The hydroperoxo formed is potentially an early LPMO catalytic reaction intermediate or the first step in the mechanism of hydrogen peroxide formation in the absence of substrate. We observe that the N-terminal amino group of the copper coordinating His1 remains doubly protonated directly following molecular oxygen reduction by copper. Aided by molecular dynamics and mining minima free energy calculations we establish that the conserved second-shell His161 in *Mt*PMO3* displays conformational flexibility in solution and that this flexibility is also observed, though to a lesser extent, in His157 of *Nc*LPMO9D. The imidazolate form of His157 observed in our structure following oxygen intermediate protonation can be attributed to abolished His157 flexibility due steric hindrance in the crystal as well as the solvent-occluded active site environment due to crystal packing. A neutron crystal structure of *Nc*LPMO9D at low pH further supports occlusion of the active site since His157 remains singly protonated even at acidic conditions.

## Abstract 2

Superoxo and hydroperoxo intermediates were cryotrapped at the copper active site of lytic polysaccharide monooxygenase using neutron protein crystallography.

## Graphical abstract

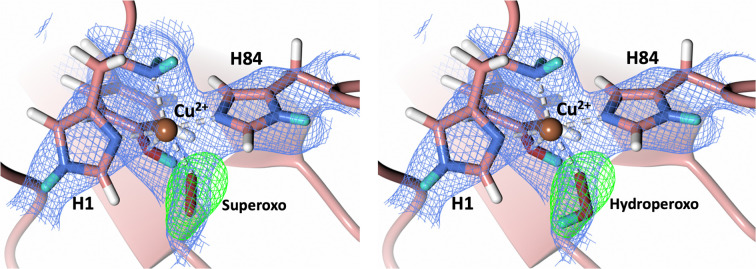

## Introduction

Lytic polysaccharide monooxygenases (LPMOs; also designated PMOs) are copper metalloenzymes that perform unique redox chemistry resulting in disruption of polysaccharide chains by oxygen atom insertion at the glycosidic bond.^1^ LPMOs originate from a range of organisms including bacteria, fungi, algae as well as animals and are classified according to Auxiliary Activity (AA),^2^ namely the AA9–AA11 and AA13–16 families. Herein we study activity of an AA9 LPMO, a family of LPMOs of fungal origin that are active on cellulose. The LPMO active site is highly conserved and contains a mononuclear Cu^2+^ center within a “histidine brace” motif in which the copper is coordinated by the N-terminal histidine amino group, the N_δ_ atom of His1 and the N_ε_ atom of a second conserved histidine in the equatorial plane.^3^ The LPMO axial position is occupied by a Tyr residue 2.5–3.0 Å from the copper center, with the exception of some AA10 LPMOs where the Tyr is replaced by a Phe.^4^ In the Cu^2+^ resting state, the remaining equatorial and axial positions are each occupied by a water molecule.^5,6^

**Scheme 1:**
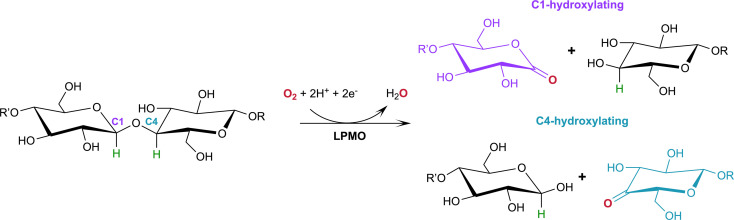
Oxidation of the C1/C4 carbon in the glycosidic bond catalyzed by LPMOs with oxygen as the co-substrate. H atoms to be abstracted by the reactive oxygen species shown in green.

Of particular commercial and biotechnological interest is the ability of fungal LPMOs to catalyze the oxidative cleavage of recalcitrant crystalline cellulose at the C1 and/or C4 position, thereby enhancing polysaccharide depolymerization by increasing glucan access to hydrolytic enzymes and ensuring efficient bioconversion.^7,8^ The overall reaction of LPMOs for oxidative cleavage of the glycosidic bond proceeds as shown in Scheme 1 in which oxygen is inserted at the C1 or C4 position which destabilizes the glycosidic bond resulting in bond breakage *via* an elimination reaction.^9^

While H_2_O_2_ has also been shown to be a LPMO co-substrate,^10–12^ the work presented here focuses on the activation of O_2_, the first characterized co-substrate in polysaccharide oxidation and the substrate in the synthesis of H_2_O_2_ in the absence of polysaccharide substrate.^9,13,14^ One-electron reduction of the copper active site to Cu^1+^ and binding of the molecular oxygen co-substrate initiates the reaction cycle with the generally accepted formation of a Cu^2+^ superoxide, [Cu–O_2_]^+^.^9,13,15,16^ In the current consensus for the O_2_-based mechanism, following the rapid reduction of molecular dioxygen to the superoxide, the LPMO reaction mechanism requires further delivery of electrons and protons to form a reactive oxygen intermediate which oxidizes the polysaccharide substrate.^17^ The identity of the activated oxygen species responsible for hydrogen atom abstraction (HAA) from the polysaccharide substrate leading to subsequent substrate oxidation, is however still a matter of ongoing research.^18^ Intensive studies encompassing X-ray crystallographic, computational and spectroscopic examinations of mononuclear copper monooxygenases as well as biomimetic copper complexes have proposed dioxygen intermediates such as the superoxide [Cu–O_2_]^+^ and hydroperoxo [CuOOH]^+^ species where the O–O bond of molecular oxygen remains intact as the reactive intermediate, however the superoxide species is unlikely to act as the HAA since this has been shown to be energetically unfavorable.^16,19–23^ Furthermore, formation of the hydroxide [CuOH]^2+^ and oxyl [CuO]^+^ intermediates after O–O bond scission, with subsequent involvement in HAA, have been proposed.^23–31^ Formation of a potential hydroperoxo, hydroxy or oxyl HAA species would require further protonation and reduction of the superoxide; however, the identity of the proton donor is still debated. Several second-shell residues including histidine (His147 in *Ls*LPMO9A/His161 in *Mt*PMO3*) as well as glutamine (Gln167 in *Mt*PMO3*) and glutamate (Glu201 in *Jd*LPMO10A) have been proposed to be involved in intermediate protonation.^31–33^ QM/MM calculations by Hedegård *et al.* propose that a positively charged second-shell His147 in *Ls*LPMO9A is involved in protonation of the superoxo intermediate.^34^ Mutagenesis and electron paramagnetic resonance (EPR) studies of His161 in *Mt*PMO3* by Span *et al.* found that His161 plays a role as a proton donor during oxygen activation. QM/MM studies of *Jd*LPMO10A have also proposed Glu201, a residue in the vicinity of the copper active site as a potential proton donor.^33^ In addition to protonation of the initially formed superoxide species *via* a second shell residue, recent quantum mechanics/molecular mechanics (QM/MM) calculations have also shown that the superoxide species can abstract a hydrogen atom from an exogenous reducing agent such as ascorbic acid to form a hydroperoxo species.^35^

Given their role in boosting polysaccharide degradation for downstream industrial applications, elucidation of intermediates in the LPMO reaction pathway will pave the way for improved utilization of LPMOs. While there have been extensive studies on the LPMO mechanism, direct characterization of catalytic intermediates remains elusive given the insoluble nature of the polysaccharide substrate and the risk of radiation damage-induced artefacts at the copper active site. Neutron protein crystallography is particularly well suited to the determination of protonation states within a protein due to the technique's unique sensitivity to hydrogen atoms and protons often invisible to X-ray diffraction.^36,37^ Neutron protein crystallography is also a non-destructive technique ideally suited for the study of radiation-sensitive metalloproteins for characterization of catalytic intermediates.^38^ The application of cryo-neutron protein crystallography to study the protonation states of activated oxygen catalytic intermediates has been demonstrated by Raven, Moody and coworkers for the characterization of compound I and compound II.^39,40^ In particular, a neutron crystallography study of cytochrome c peroxidase (CcP) resulted in cryo-capture and characterization of a deprotonated Fe(iv)=O compound I species in a 2.5 Å resolution structure.^41^ In further study, a potential protonated Fe(iv)–OH compound II species was observed in an dioxygen activated and cryocooled structure of ascorbate peroxidase (APX) at a 2.2 Å resolution.^42^

We report here two neutron models of a carbohydrate-free C4-hydroxylating *Neurospora crassa* LPMO9D: a structure of the activated, ascorbate-reduced form and a structure of the resting state at pH 4.4 (pD 4.8) to further address open questions regarding initial molecular oxygen activation. The neutron crystallographic structure of the reduced *Nc*LPMO9D shows cryo-capture of activated dioxygen at the copper active site modelled as a superoxo and protonated hydroperoxo species with a population ratio of 0.30 : 0.70. The neutron scattering length density maps of the conserved secondary coordination shell His157 is best represented as 70% imidazolate and a 30% singly protonated form, indicating the His157 acts as a proton donor during early dioxygen intermediate formation. This imidazolate intermediate state was found to be stable following DFT calculations. The copper coordinating N-terminal amino group His1 remains in a doubly protonated ND_2_ state following copper reduction and molecular oxygen activation. We probed the flexibility of His157 in our structure and the corresponding His161 in *Mt*PMO3* using molecular dynamics (MD) simulations to further explore their function during initial oxygen activation. His161 in *Mt*PMO3* displayed mobility between an inward and outward conformation, with His157 in our structure displaying similar mobility but to a lesser extent. Mining minima free energy calculations further indicate His157 flexibility in solution which is absent in the crystal structure due to binding-face to binding-face crystal packing. Furthermore, the neutron and X-ray crystal structures of the resting state *Nc*LPMO9D at low pH show that His157 remains singly protonated when exposed to acidic conditions. The additive effect of limited mobility and active site occlusion due to crystal packing have therefore permitted trapping of a hydroperoxo intermediate and designation of the second-shell His157 as the proton donor.

## Results and discussion

### Crystallographic features of the *Nc*LPMO9D neutron structures

**Fig. 1:**
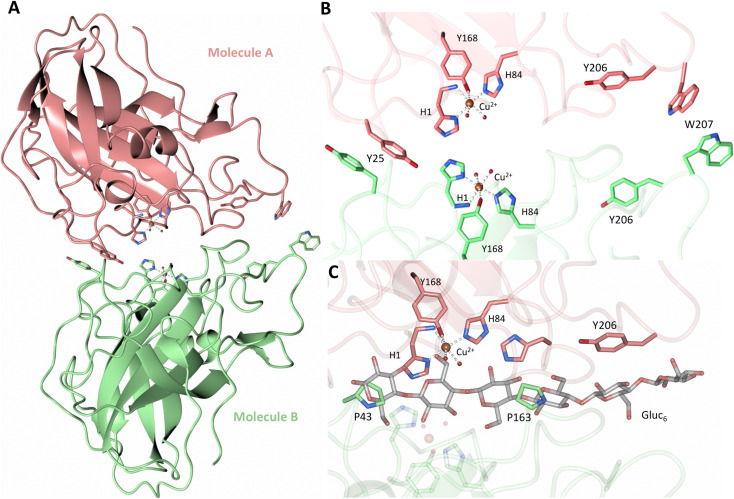
Crystal packing of molecule A and molecule B in the *Nc*LPMO9D crystals. (A) Cartoon of molecule A (coral) and molecule B (light green) of the planar active site surfaces packed facing each other. (B) Crystal symmetry in molecule A and B is broken by alternate conformations of Tyr 25, Tyr206 and Trp207. (C) Alignment of molecule B of *Nc*LPMO9D with the Gluc_6_ bound *Ls*LPMO9A (PDB 5ACI) in grey to illustrate superposition of proline residues with glucose pyranose rings.

**Fig. 2:**
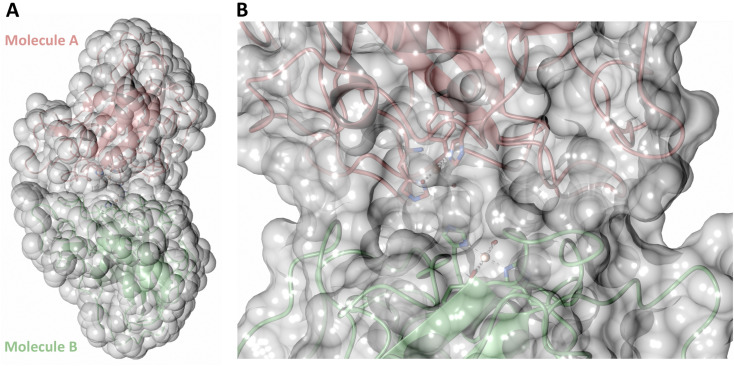
Solvent accessible surface in the crystal packing of molecule A and molecule B of the asymmetric unit of *Nc*LPMO9D. Surface displayed with a probe radius of 1.5 Å.

Both the ascorbate-reduced and low pH neutron crystals of *Nc*LPMO9D belonged to the monoclinic *P*2_1_ space group. The crystal structures include two protein molecules per asymmetric unit (molecule A and molecule B) related by non-crystallographic symmetry (NCS). Analysis of the crystallographic contacts shows that the two NCS related *Nc*LPMO9D molecules pack with their planar polysaccharide binding interfaces against each other in a binding-face to binding-face configuration also seen in PDB structures 4EIR, 5TKG, 5TKH and 5TKI (Fig. 1A).^15,43,44^ While present in a dimeric conformation in the crystal structure, LPMOs including *Nc*LPMO9D function as single-domain monomeric proteins in solution, where they associate with planar crystalline cellulose as confirmed by early LPMO structural studies,^8,15,45^ and single-molecule observations of LPMO using atomic force microscopy (AFM).^46^ Within this binding-face to binding-face packing architecture, the two coordinated coppers are ∼12 Å apart, precluding direct interaction. Binding interface residues Tyr25, Tyr206 and Trp207 adopt distinct conformations in molecule A and B, contributing to different microenvironments near the Cu^2+^ active site (Fig. 1B). Structural alignment of our *Nc*LPMO9D molecule A or molecule B with the oligosaccharide-bound form of *Ls*LPMO9A (PDB 5ACI),^47^ indicates that the substrate pyranose rings of the *Ls*LPMO9A structure superimpose with the proline rings Pro43 and Pro163 for both molecule A or molecule B of *Nc*LPMO9D (Fig. 1C). The alignment further indicates that Tyr206 of molecule A is positioned to interact with substrate *via* its phenol ring. This suggests that the crystallographic environment of the active site in molecule A more closely mimics surface residue interaction with the pyranose ring of the glucose units and creates an occluded active site, possibly analogous to the bulk solvent-protected environment created when the planar active site of LPMO binds to its crystalline linear polysaccharide substrate. Such crystal packing in which active site residues mimic carbohydrate substrate pyranose rings has also been observed in the crystal packing of *Nc*LPMO-3 (PDB 4EIS).^15^ Representation of the protein surface additionally illustrates the active site occlusion due to crystal packing (Fig. 2).

### Ascorbate-reduced copper active site

**Fig. 3:**
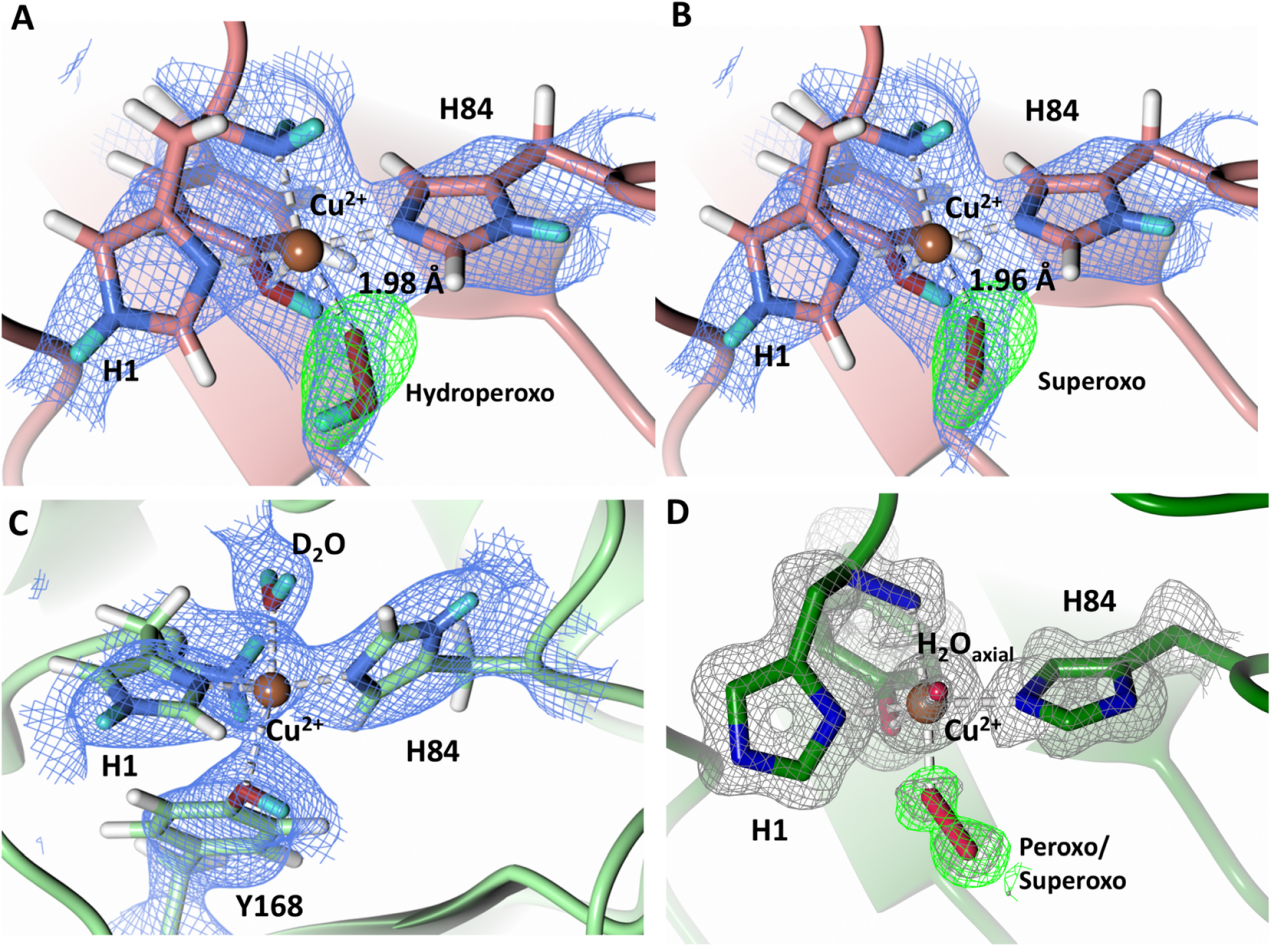
Neutron diffraction structure of the ascorbate reduced *Nc*LPMO9D crystal (PDB 7T5D). (A) Active site of molecule A (coral) with a hydroperoxo species bound at the equatorial position. (B) Active site of molecule A (coral) with a superoxo species bound at the equatorial position. (C) Active site of molecule B (light green) with a water bound at the axial position. (D) Equatorially coordinated activated dioxygen superoxo/peroxo in X-ray diffraction structure of ascorbate reduced *Nc*LPMO9D (green) by O'Dell *et al.*^43^ (PDB 5TKH). (A–C) Neutron scattering length density 2*F*_o_–*F*_c_ maps displayed in blue at a 1.0 *σ* cut-off. Neutron scattering length density *F*_o_–*F*_c_ maps displayed in green at a 2.5 *σ* cut-off. H atoms displayed in white and D atoms displayed in turquoise. (D) Electron density 2*F*_o_–*F*_c_ maps displayed in grey at a 1.0 σ cut-off. Electron density *F*_o_–*F*_c_ maps displayed in green at a 3.0 *σ* cut-off.

To probe the protonation states of key catalytic residues and intermediates in the *Nc*LPMO9D active site, a neutron diffraction dataset to 2.4 Å resolution was collected at 100 K on a crystal of *Nc*LPMO9D reduced with ascorbate at pD 6.0 (pH 5.6) in the presence of atmospheric oxygen. Structure refinement was performed solely against neutron data to circumvent potential artefacts induced by X-ray radiation damage. The axial position is vacant, supporting displacement of both the equatorial and axial water molecules upon Cu^2+^ reduction and dioxygen activation as demonstrated by Kjaergaard *et al.* using extended X-ray absorption fine structure (EXAFS), X-ray Absorption Near Edge Spectroscopy (XANES) and density functional theory (DFT) calculations.^16^ The neutron scattering length density maps at the active site of molecule A indicate the presence of a fully occupied equatorially coordinated dioxygen species in a mixed state of a two-atom superoxo species and a three-atom hydroperoxo species with occupancies of 0.30 and 0.70, respectively (Fig. 3A and B). The intermediates have *η*_1_ end-on geometry at a Cu–O1 distance of ∼1.96 Å and ∼1.98 Å for the superoxo and hydroperoxo species, respectively. The superoxo species is modelled with an O–O bond length of 1.28 Å while the hydroperoxo species is modelled with a bond length of 1.46 Å. The Cu–O1–O2 angles are ∼147° and 151° for the superoxo and hydroperoxo species respectively. A Glu30 residue from molecule B is modeled 2.06 Å and 2.08 Å away from the O2 of the superoxo and hydroperoxo species, respectively, however it displays a disordered conformation with limited neutron scattering length density, as has been observed for *Nc*LPMO9D structures with the same crystal packing.^15,43,44^ The modeling of an activated dioxygen species is in good accordance with our previous high resolution X-ray structures which indicated the presence of a peroxo species with a 1.44 ± 0.06 Å bond length with a Cu–O1–O2 angle of 140.52° and 1.90 Å from the Cu following ascorbate reduction (Fig. 3D).^43^ Activated dioxygen species have been modelled in further LPMO structures including *Nc*LPMO9D and *Nc*PMO-3 from Li *et al., Jd*LPMO10A from Bacik *et al.* as well as in an artificial copper protein from Mann *et al.* (Table 1).^15,20,33,43,48^ Most recently, an EPR/electron–nuclear double resonance (ENDOR) spectroscopy study by Davydov *et al.* of copper coordination complexes captured a Cu(i)-superoxo species that undergoes internal electron transfer to form a Cu(ii)-peroxo species which is protonated to form the Cu(ii)-hydroperoxo species.^49^ These species were captured in end-on copper coordination corresponding to the equatorial end-on coordination observed for the hydroperoxo species in the structure presented here. In our previous high-resolution X-ray structure, discrimination between a putative superoxo and peroxo species was based on bond length determination due to the lack of visibility of hydrogen atoms. In addition, the occupancy of the activated dioxygen species only refined to 0.59 and an axial water remained present with an occupancy of 0.48. We reason that photoreduction due to X-ray beam exposure resulted in an intermediate with reduced occupancy. The *Nc*LPMO9D active site presented here is absent of radiation damage since neutron protein crystallography is a non-destructive technique permitting capture of a mixed occupancy of a superoxo and hydroperoxo species.^37^ The observed hydroperoxo species may represent an early dioxygen intermediate in the LPMO catalytic mechanism, or it may be an intermediate in LPMO-catalyzed H_2_O_2_ formation, since LPMOs are known to produce H_2_O_2_ in the absence of substrate.^50^ Caldararu *et al.* have further shown it is more energetically favorable for H_2_O_2_ formation to proceed by dissociation of H_2_O_2_ from the copper active site than HO_2_.^33^ This higher dissociation energy of HO_2_ is supported by the observed binding stability of the hydroperoxo species in our structure.

**Table 1:** Structurally modelled dioxygen species in LPMOs

LPMO	PDB	Dioxygen species	Position	Cu–O (Å)	O1–O2 (Å)	Cu–O–O (°)
*Nc*LPMO9D	4EIR – chain A	Superoxide – end-on	Axial	2.96	1.16	147.75
	4EIR – chain B	Superoxide – end-on	Axial	2.92	1.15	142.11
*Nc*PMO-3	4EIS	Peroxo – end-on	Axial	3.44	1.49	117.16
*Jd*LPMO10A	5VG0 – Chain A	Peroxo – side-on	Equatorial	O1 – 2.14, O2 – 1.84	1.48	O1 – 57.67, O2 – 79.39
*Jd*LPMO10A	5VG0 – Chain A	Peroxo – end-on	Equatorial	1.83	1.46	109.52
*Jd*LPMO10A	5VG1 – Chain B	Peroxo – end-on	Equatorial	2.49	1.45	115.32
	5VG0/5VG1 joint quantum refinement (ComQum-X) – Chain A	Superoxide – end-on	Equatorial	2.31	1.25	108.6
	5VG0/5VG1 joint quantum refinement (ComQum-X) – Chain B	Superoxide – end-on	Equatorial	2.27	1.25	113.6
*Nc*LPMO9D	5TKH – Chain A	Peroxo – end-on	Equatorial	1.90	1.44	140.52
Artificial copper protein	6ANX	Hydroperoxo	Intermediate	1.94	1.52	137.60
*Nc*LPMO9D (current neutron structure)	7T5D – Chain A	Superoxo	Equatorial	1.96	1.28	147
	7T5D – Chain A	Hydroperoxo	Equatorial	1.98	1.46	151

The molecule B copper active site contains a water molecule coordinated in the axial position with a partial occupancy of 0.65 and a Cu–O distance of ∼2.2 Å while the equatorial position is vacant (Fig. 3C). This geometry, while not chemically relevant, has been observed in partially photo-reduced LPMO structures,^6^ suggesting that the active site of molecule B underwent only partial reduction during the ascorbate soak. We attribute the differences between molecule A and molecule B to the conformations of the Tyr residues at the binding interfaces.

### Protonation state of His157

**Fig. 4:**
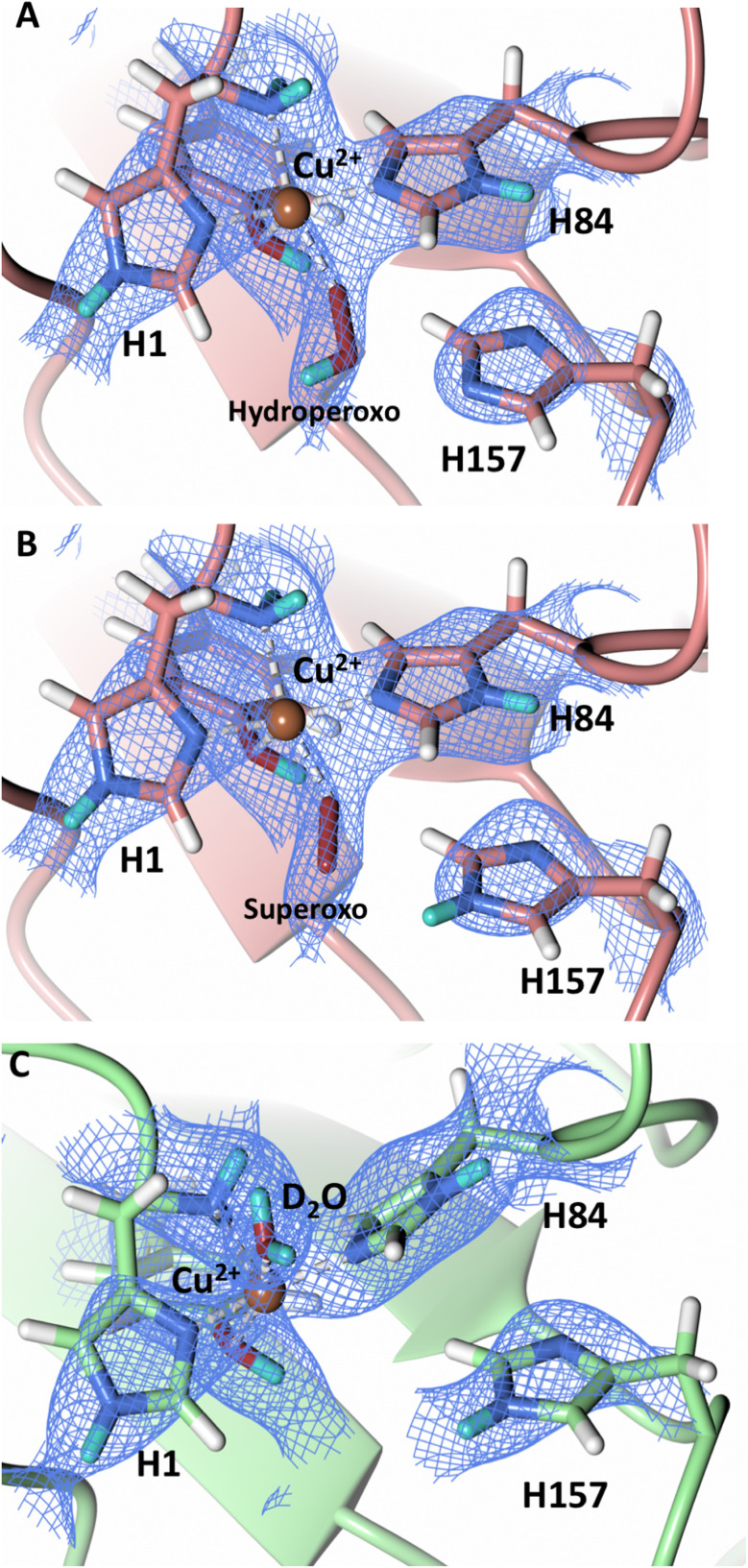
Mixed protonation state His157 in ascorbate reduced *Nc*LPMO9D (PDB 7T5D). (A) The molecule A (coral) second shell His157 is deprotonated with an equatorial hydroperoxo species coordinated to Cu^2+^. (B) The molecule A second shell His157 is singly N_ε_-protonated with an equatorial superoxo species coordinated to Cu^2+^. (C) The molecule B (light green) second shell His157 is singly N_ε_-protonated with an axial water coordinated to Cu^2+^. Neutron scattering length density 2*F*_o_–*F*_c_ maps displayed in blue at a 1.0 *σ* cut-off. H atoms displayed in white and D atoms displayed in turquoise.

Testing of different protonation states and visual examination of the 2*F*_o_–*F*_c_ and *F*_o_–*F*_c_ omit neutron scattering length density maps of the molecule A secondary coordination shell His157 suggest that this residue is best modelled by the neutral N_ε_-protonated form and the imidazolate form with occupancies of 0.30 and 0.70, respectively (Fig. 4A and B). Analysis of the molecule B neutron scattering length density maps indicate that His157 is singly N_ε_-protonated at full occupancy (Fig. 4C). The N_ε_ nitrogen of His157 in molecule A is observed at a distance of ∼3.0 Å from the distal oxygen O2 of the equatorially bound superoxo species and hydroperoxo species. These findings indicate that His157 plays a role in protonation of the activated dioxygen species observed in the barricaded active site presented here. While an imidazolate species may be an unusual catalytic intermediate, it has been determined that the p*K*_a_ of a deprotonated His is 14, while the peroxo group is very basic with a p*K*_a_ of up to 24 (as found in hydroperoxide dicopper complex), supporting the donor role of a neutral His proposed herein.^51,52^ Activity and structural studies of *Mt*PMO3* as well as QM/MM calculations of *Ls*LPMO9A have implicated this conserved histidine residue in proton transfer to the copper-bound superoxide during LPMO catalysis (His161 and His147 in *Mt*PMO3* and *Ls*LPMO9A, respectively).^31,32^ QM/MM studies have also indicated that the Glu201 residue near the copper active site in *Jd*LPMO10A is capable of donating a proton to a bound superoxo to form a hydroperoxo species.^33^ Computational studies have suggested that ascorbate may function as a proton donor to the superoxo species;^35^ however, the active site occlusion due to crystal packing in our structure does not permit ascorbate to access the copper active site, and there was no evidence in the neutron scattering length density maps for an ascorbate molecule near the active site. Following formation of the superoxide in *Nc*LPMO9D, the species we observe may result from protonation by His157 to form the Cu(ii)-hydroperoxyl species [CuOOH]^2+^, the Cu(iii)-hydroperoxo [CuOOH]^2+^, or upon further reduction a Cu(ii)-hydroperoxo [CuOOH]^+^, *i.e.*Superoxo: [Cu]^+^ + O_2_ → [Cu–O_2_]^+^ with Cu(ii) centerHydroperoxyl radical/Hydroperoxo: [Cu–O_2_]^+^ + H^+^ → [Cu–OOH]^2+^ with Cu(ii)/Cu(iii) centerHydroperoxo: [Cu–O_2_]^+^ + H^+^ + e^−^ → [Cu–OOH]^+^ with Cu(ii) center

All these species have been posited as viable reaction intermediates in computational and small molecule studies.^20,23,49,53–55^

#### Density functional theory (DFT) calculations

While the neutron data supports modeling of a two and three atom dioxygen species at the active site, it does not, however, permit discrimination between the hydroperoxyl or hydroperoxo species just discussed nor does it resolve the redox state of the copper ion. We performed DFT calculations on active site ‘cutout’ models to further assess the proposed (based on our neutron data and previous work) intermediates in the copper active site of molecule A. The aim of the DFT calculations was to qualitatively investigate the stability of different intermediate states with respect to proton dissociation/transfer and rule out any that were found to be unstable. To this end, local minimum energy searches were performed by DFT geometry optimization of the ‘cutout’ active site models, which comprised the copper center plus the two or three atom dioxygen species, and the surrounding residues His1, His84, His157, Gln166, and Tyr168. Appropriate protonation states, along with total charge and multiplicities, were set providing the following models: Cu(ii)-superoxo species with singly protonated His157, Cu(ii)-hydroperoxyl radical with imidazolate His157, Cu(ii)-hydroperoxo with imidazolate His157, and Cu(i)-hydroperoxo with imidazolate His157 – see Table 2. (Note that we investigated the second reduction to form the Cu(i)-hydroperoxo species as it can potentially be derived from ascorbate by long-range intramolecular electron transfer to the active site, and it has been proposed to occur *via* LPMO aromatic residues localized near the copper active site.^14,15,56,57^) Further DFT calculation details including the basis set *etc.* are provided in the Methods section.

**Table 2:** Details of the DFT models

Model	DFT active site residues	Starting proton position	System net charge	Multiplicity	Final His157 state
Superoxo, His157 NE2-protonated	His1, His84, His157, Gln166, Tyr168	His157 NE2	+1	Triplet	His157 singly NE2-protonated
Hydroperoxyl radical, His157 doubly deprotonated	His1, His84, His157, Gln166, Tyr168	O–O–H	+1	Triplet	Double deprotonated His157
Hydroperoxo, His157 doubly deprotonated	His1, His84, His157, Gln166, Tyr168	O–O–H	0	Doublet	Double deprotonated His157
Hydroperoxo, His157 doubly deprotonated	His1, His84, His157, Gln166, Tyr168	O–O–H	−1	Singlet	Double deprotonated His157

The DFT calculation results are summarized in Table 2. All the active-site models considered showed energy and energy-gradient convergence to a stationary point on the potential energy surface with the proton still located at its initial position and so are considered stable and none are ruled out. More details of the energy convergence profiles are provided in the ESI.† We note that the current DFT calculations/‘cutout’ models do not provide a means of directly comparing the relative energies of the proposed intermediates due to the different combinations of total charge and spin states across the four models. Nor can the possible Cu(iii)-hydroperoxo with imidazolate His157 intermediate be adequately examined, due to probable multi-determinant character.^58^ Accurate calculated energy differences between all the intermediates will require a multi-determinant QM treatment due to propensity of copper centers to exhibit multiple near degenerate electronic states,^58,59^ as well as a more complex structural model *e.g.* inclusion of an electron donor molecule.^23^ This will be addressed in planned future calculations using QM/MM with a multi-determinant wavefunction QM method.

### Active site protonation following initial dioxygen activation

**Fig. 5:**
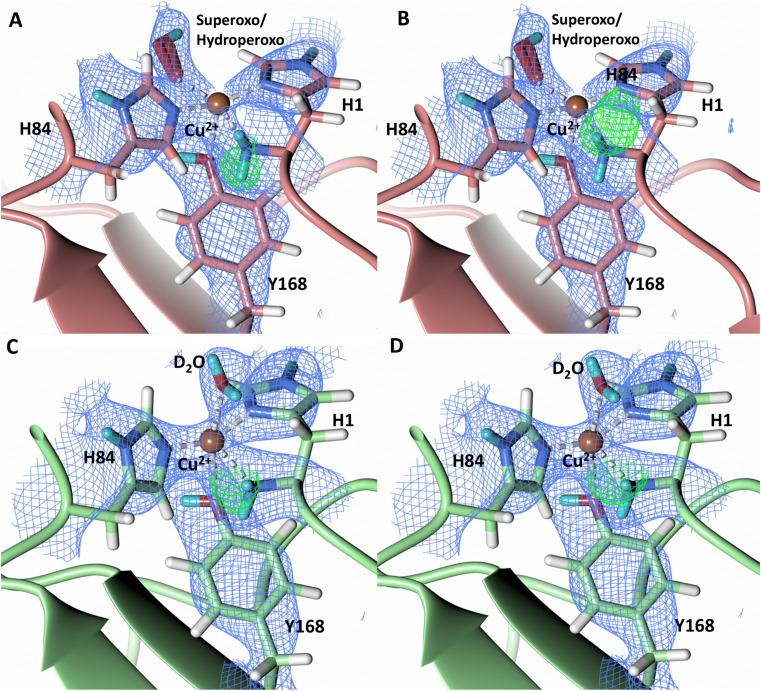
Protonation state of the N-terminal His157 amino group in the ascorbate reduced *Nc*LPMO9D crystal (PDB 7T5D). (A) Molecule A (coral) omit map displaying D2 proton and (B) D3 proton of the N-terminal nitrogen. (C) Molecule B (light green) omit map displaying D2 proton and (D) D3 proton of the N-terminal nitrogen. Neutron scattering length density 2*F*_o_–*F*_c_ maps displayed in blue at a 1.0 *σ* cut-off. Neutron scattering length density *F*_o_–*F*_c_ maps displayed in green at a 3.0 *σ* cut-off. H atoms displayed in white and D atoms displayed in turquoise.

The N-terminal amino group of the histidine brace has been postulated to play a role in intermediate formation and stabilization during LPMO catalysis.^60^ A previous neutron protein crystallography study of unreduced *Jd*LPMO10A, a bacterial LPMO, suggested that the amino terminal is present as a mixture of the -ND^−^ and -ND_2_ protonation states.^48^ The *Jd*LPMO10A neutron structure was, however, later revisited and a joint X-ray–neutron quantum refinement concluded that the amino terminal is present solely in the -ND_2_ state.^33^ We therefore sought to determine the protonation state of the N-terminal amino group upon *Nc*LPMO9D reduction by ascorbate in the presence of atmospheric O_2_. The neutron scattering length density map indicates that both molecule A and molecule B contain the amino terminal in the -ND_2_ protonation state. *F*_o_–*F*_c_ omit difference maps calculated confirm the ND_2_ state in both molecule A and molecule B (Fig. 5A–D). The role of possible deprotonation of the N-terminal histidine amino group came to the forefront when it was proposed to promote reactive intermediate formation and stabilization based on findings from a small molecule copper complex.^61^ Structural and spectroscopic studies of a substrate-bound *Ls*LPMO9A suggested that the two protons of the N-terminus are exposed to different chemical environments, with one being involved in a hydrogen bond network, which potentially promotes N-terminal deprotonation during LPMO catalysis.^47^ Our neutron protein crystallography structures show that the N-terminal amine is not involved in protonation upon initial Cu^2+^ reduction and molecular dioxygen activation. However, a mechanistic role for the N-terminal amine in other contexts cannot be conclusively ruled out by our experiments.

**Fig. 6:**
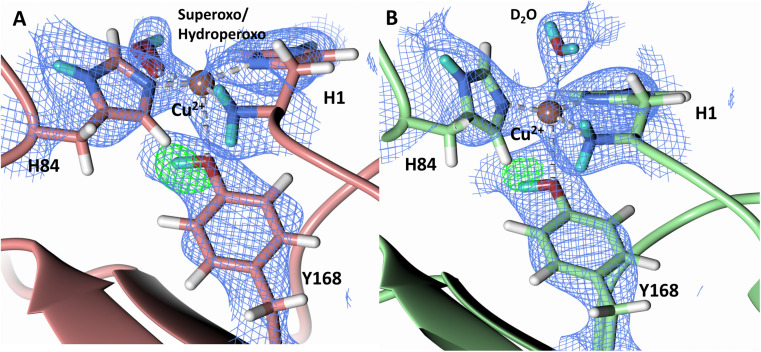
Protonation state of the Tyr168 in the ascorbate reduced *Nc*LPMO9D crystal (PDB 7T5D). (A) Molecule A (coral) omit map displaying Oη protonation and (B) Molecule B (light green) omit map displaying Oη protonation. Neutron scattering length density *F*_o_–*F*_c_ maps displayed in green at a 3.0 *σ* cut-off. H atoms displayed in white and D atoms displayed in turquoise.

A study by Paradisi *et al.* on *Ls*AA9 spectroscopically detected a radical Cu^2+^-tyrosyl complex in which the axial Tyr164 was present as a deprotonated radical following reaction with H_2_O_2_ following ascorbate reduction.^62^ This tyrosyl radical intermediate formed irreversibly and was inactive toward substrate, indicating that it may play a role in active site protection during uncoupled turnover. A further spectroscopic study on *Ta*LPMO9A by Singh *et al.* isolated a Cu^2+^-tyrosyl radical intermediate following ascorbate reduction and addition of H_2_O_2_.^63^ The long-lived Tyr175 radical intermediate was spectroscopically distinct from the tyrosyl radical observed in *Ls*AA9 and was proposed to play a role in active site protection from oxidation or be involved in substrate oxidation. To address the observation of the two radical species, McEvoy *et al.* performed QM/MM to model the feasibility of both deprotonated tyrosyl radical species.^64^ The intermediate observed by Singh *et al.* was proposed to be to a *cis*-[Tyr-CuOH]^+^ formed by hydrogen transfer from the tyrosine hydroxy to an oxyl species in the equatorial plane. The intermediate observed by Paradisi *et al.* was characterized as a *trans*-[Tyr-CuOH]^+^ which can be formed through internal hydrogen transfer from a water molecule axially coordinated to the copper. Calculations of reactivity of these tyrosyl radical species toward the substrate however indicated limited reactivity, further supporting a protective role for tyrosine to prevent oxidative damage. Given these observations of a tyrosine acting as a proton donor to form a deprotonated phenoxyl species, we further examined the protonation state of Ty168 in the copper axial position of *Nc*LPMO9D. *F*_o_–*F*_c_ omit difference maps in both molecule A and B indicate Tyr168 remains in the protonated form (Fig. 6), excluding it as a proton donor at the early stages of dioxygen activation.

### Histidine protonation at acidic conditions

**Fig. 7:**
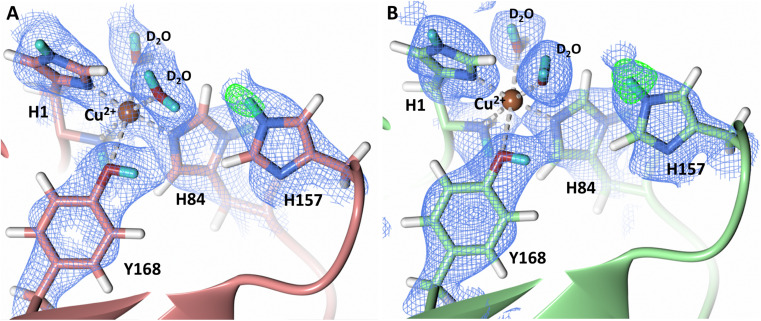
Protonation state of second-shell His157 at acidic conditions (PDB 7T5E). (A) Molecule A (coral) His157 N_ε_-protonation. (B) Molecule B (light green) His157 N_ε_-protonation. Neutron scattering length density 2*F*_o_–*F*_c_ maps displayed in blue at a 1.0 *σ* cut-off. Neutron scattering length density *F*_o_–*F*_c_ maps displayed in green at a 3.0 *σ* cut-off. H atoms displayed in white and D atoms displayed in turquoise.

During LPMO catalysis, a second shell histidine has been proposed to be involved in protonation of active site intermediates with QM/MM studies by Hedegård and Ryde on *Ls*AA9A suggesting that His147 in a doubly protonated state can act as a proton donor.^31^ Additionally, Span *et al.* have proposed that His161 in *Mt*PMO3 plays a role as a proton donor based on structural, mutagenesis and spectroscopic studies as well as activity assays.^32^ LPMOs are expected to catalyze oxygen insertion under acidic conditions since they function synergistically alongside cellulases which show optimum activity at pH 3.5–5.5.^65–67^ Given the surprising role of a neutral His157 as a proton donor to the activated dioxygen species in our structure, we sought to probe the protonation state of His157 when exposed to acidic buffer conditions. We collected a 2.14 Å room-temperature neutron diffraction dataset under low pH conditions (pH 4.4/pD 4.8) achieved by vapor exchange of the crystal with acidified buffer. Analysis of the neutron scattering length density maps indicate that the His157 residue remains singly protonated at the N_ε_-position at this pH, which is supported by *F*_o_–*F*_c_ omit difference maps in both molecule A and B (Fig. 7). To further support our acidic pH structure obtained by vapor exchange, a 1.5 Å X-ray dataset was collected on a crystal directly soaked in acidic buffer. The position of His157 at pH 4.4 superimposes to its position at pH 6.0. In this position, N_δ_-protonation would sterically clash with the backbone amide proton, further supporting the single N_ε_-protonation state observed in our neutron structure (Fig. S6 and S7†) which coincides with unrestrained refinement studies of the protonation state of second-shell His residues.^68^ The barricaded active site observed in crystallo and electropositive environment of the copper potentially play a role in maintaining the observed single histidine protonation state and prevent formation of protonation pathways to the active site. In both the neutron and X-ray low pH structures, the copper remains coordinated in the histidine brace, contrary to the disordered His78 observed in *Ls*LPMO9A at pH 3.5 (PDB 5N04).^69^ The low pH structure of *Ls*LPMO9A was obtained using high NaCl concentrations, however, resulting in His78 displacement by a chloride anion. The absence of NaCl in our crystallization conditions precludes coordinating histidine residue displacement by a chloride anion.

### Conformational flexibility of His157

**Fig. 8:**
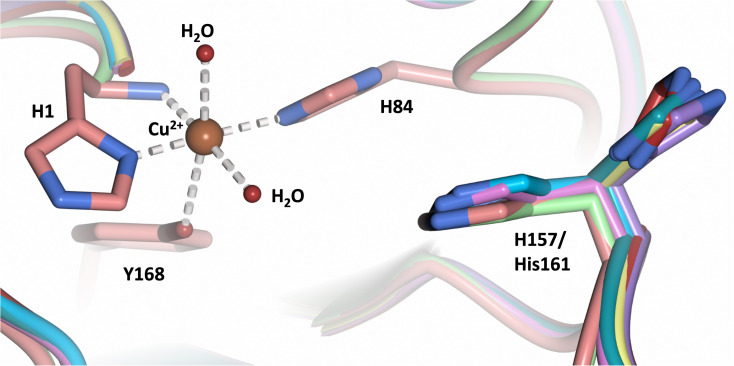
Alignment of a second shell residue His157 in the “inward” and “outward” conformation. Alignment of molecule A *Mt*PMO3* His161 (PDB 5UFV) molecule A (teal), molecule B (yellow), molecule C (pink), molecule D (purple), molecule E (turquoise) and molecule F (red) with *Nc*LPMO9D His157 (PDB 7T5C) from molecule A (coral) and molecule B (green).

Analysis of the crystal structure of *Mt*PMO3* deposited by Span *et al.* indicates that the second shell His161, corresponding to *Nc*LPMO9D His157, is present in two conformations: an “inward” copper-facing conformation with a with a Cu–N_ε_ distance of ∼5 Å, and an “outward” copper-distant conformation with a Cu–N_ε_ distance of ∼10 Å (Fig. 8).^32^ The experimentally observed existence of both “inward” and “outward” conformations indicates mobility of the conserved second shell histidine, at least when the copper active site is solvent exposed. It may also suggest a mechanism whereby the “outward” second shell histidine can “collect” a proton from solvent (becoming doubly protonated) and, in turn, the “inward” conformation donates a proton in the active site.^31,32^ In this work we have established that in the dimeric packing of *Nc*LPMO9D in the crystal, unusually, neutral His157 donates a proton to active site intermediates, and, furthermore, that it cannot become doubly protonated even under acidic conditions. We therefore conjectured (see previous section) that in the *Nc*LPMO9D dimer, His157 is blocked (or barricaded) from “collecting” a proton from solvent. It does, however, seem possible, that in the unhindered monomer form of *Nc*LPMO9D, a flexible His157 conformation dependent proton transport mechanism would be available.

To examine computationally the possibility that *Nc*LPMO9D His157 can exhibit “inward” and “outward” conformations when in monomeric form, and whether it is likely that dimer crystal packing occludes such flexibility, we used two different approaches: MD simulations and VM2 mining minima free energy calculations – see Methods section. The goal of the MD calculations was to establish whether inward/outward conversion is possible on the microsecond timescale. The mining minima calculations, unlike MD, can easily traverse high energy barriers during conformational searches. Therefore, it was employed to establish, regardless of kinetic barrier/timescale, whether inward/outward conformations exist or not in the monomer and dimer forms, and if so, what their relative energies are. It was also used to estimate an energy barrier to interconversion in the monomer.

#### Molecular dynamics (MD) simulations

The flexibility of His157 (or equivalent) in monomeric LPMO was investigated using microsecond timescale MD simulations. Three different 1.0 μs MD simulations were performed: two of these were based on the neutron diffraction structure reported here, *Nc*LPMO9D, and the third was based on a related LPMO structure (*Mt*PMO3*, PDB ID = 5UFV). The MD simulations were performed using explicit solvent and counter-ions using AMBER's ff14SB force-field (more details are available in the Methods section). Weak positional restraints were applied to Cu to keep it in the active site.

**Fig. 9:**
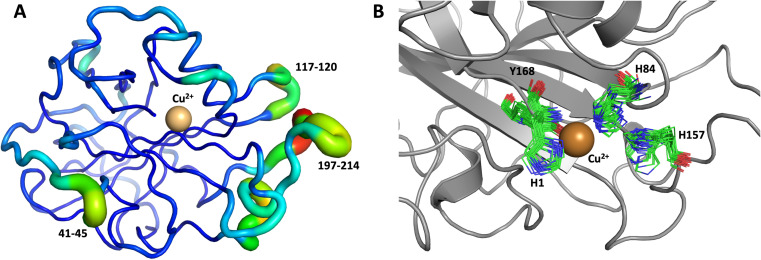
Conformational flexibility of LPMO and active-site residues. Results from 1 μs MD simulation, starting from the reported neutron structure. (A) Conformational flexibility of the entire protein calculated based on root mean square fluctuations of slowest 10 modes (RMSF_10_). Regions shown as thin blue tubes are rigid while colored regions with thicker green/yellow/red tubes show highest flexibility (regions labeled show largest flexibility). (B) Active site residues (only 20 frames spaced at regular interval of 50 ns are shown).

The first 1.0 μs MD simulation, starting from the neutron structure reported here, showed a stable “inward” His157 conformation, staying in this original “inward” conformation during the entire MD trajectory. We note that although restraints were applied to keep Cu in the active site, the interacting residue conformations seen in the simulation were not adversely affected and are consistent with their positions in the neutron structure. The most flexible regions of the protein are 41–45, 117–120 and 197–214 (Fig. 9A). Representative conformations of the active site are shown in (Fig. 9B) and indicate little movement in that region.

**Fig. 10:**
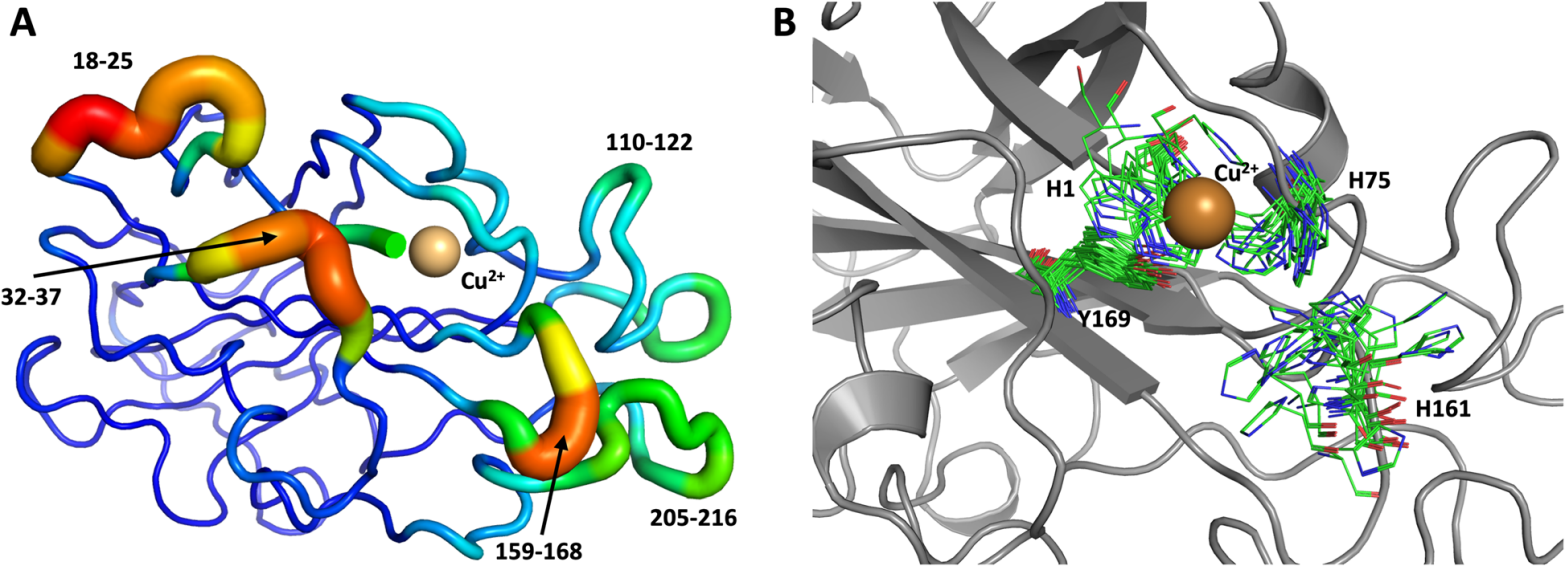
Conformational flexibility of a related LPMO and active-site residues (MtPMO3* PDB 5UFV) results from 1 μs MD simulation. (A) Conformational flexibility of based on RMSF_10_. (B) Active site residues. His161 (equivalent to His157) is highly flexible and transition back and forth between the inward and outward conformations during the 1 μs MD simulation (see Fig. 9 legend for more details).

To obtain further insight, another 1.0 μs MD simulation, based on the related LPMO *Mt*PMO3* (PDB ID = 5UFV, monomer A), was performed. This MD simulation indicated that His161 (at the equivalent position to His157 in our neutron structure) is able to freely interconvert between the “inward” and “outward” conformations at the microsecond timescale (Fig. 10). Notably, the active site shows significantly more movement, and more than 10 transitions between inward and outward conformations were observed over the course of the whole simulation (Fig. 10B). Therefore, we hypothesized that His157 in our structure is possibly trapped in a conformation that makes it difficult to explore outward conformations at the microsecond timescale, *i.e.*, there is a larger kinetic barrier to transition than for 5UFV. Importantly, for the MD simulation based on 5UFV, restraints between Cu and the surrounding residues were equivalent to those used in the first simulation, which excludes the possibility that the lack of interconversion between the inward and outward conformation in MD based on our structure is due to the use of distance restraints. Overall, we observed that *Mt*PMO3* (5UFV) is, according to the simulation, a lot more flexible than our neutron diffraction structure, with the regions 18–25, 32–37, 110–122, 159–168 and 205–216 showing high flexibility (Fig. 10A). Given that the forcefield and water model are identical, and the only difference is the protein sequence, we are confident that the *Mt*PMO3* second coordination shell residues display more overall flexibility than *Nc*LPMO9D at the microsecond timescale. This difference in flexibility is consistent with the interconversion behavior of His157/161 as well. (See ESI† for representative conformations of the active site).

**Fig. 11:**
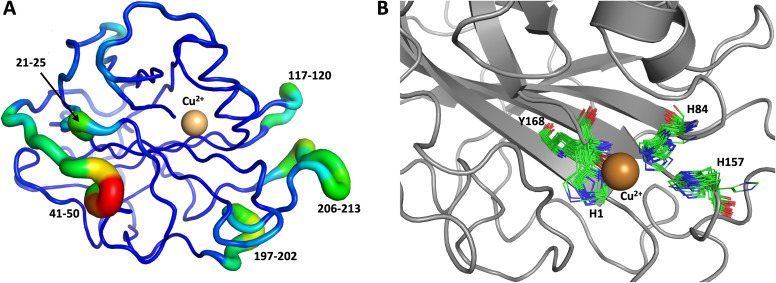
Conformational flexibility of LPMO and active-site residues (starting from His157 in outward conformation). Results from 1 μs MD simulation. (A) Conformational flexibility of based on RMSF_10_. (B) Active site residues. His157 is able to explore different conformations and transition into the inward conformation during the 1 μs MD simulation. See Fig. 9 legend for more details.

We performed another 1.0 μs MD simulation based on our neutron scattering LPMO structure but with an outward His157 conformation, in order to characterize the flexibility of His157 when starting from the outward conformation. The starting outward conformation was generated using the VM2 mining minima software, as described in detail below. This system is identical to the first MD simulation discussed above (same topology file with the same number of atoms) except for the starting conformation. In this simulation, His157 was able to interconvert to the inward conformation at the microsecond timescale only twice (Fig. 11), confirming our hypothesis of some dynamic interconversion of His157 (Fig. 11B) albeit at a lower rate than that observed for *Mt*PMO3*. Once fully in the inward conformation, His157 stayed locked in that position, suggesting the presence of an energy barrier to interconversion that limits occurrence on the 1.0 μs timescale at least. It is interesting that the two related LPMOs show a difference in the rates of His157 (and equivalent His161) conformation interconversion. This interconversion rate could have a functional role, as it may affect the sampling of certain conformational events required in the catalytic cycle, as has been observed in several other enzyme systems.^70^

#### Mining minima (VM2) free energy calculations

To further probe the extent of His157 flexibility and the associated energetics in our own structure, we carried out 2nd-generation mining minima (VM2) free energy calculations,^71–74^ and compared the predicted thermodynamically accessible *Nc*LPMO9D His157 conformations found when the active site is solvent exposed to those found when the active site is in the crystal structure with binding-face to binding-face packing. The VM2 mining minima method is well suited to this task because it is designed to overcome large energy barriers during conformational searching – see Methods section and references therein. Previous applications of VM2 include calculation of inhibitor binding free energies for HIV-1 protease, phosphodiesterase 10a, MAP kinase p38, Breast-Cancer-Gene 1 (BRCA1) C-Terminal (BRCT), cyclin-dependent kinase 8 and cyclin C.^71–73,75^

**Fig. 12:**
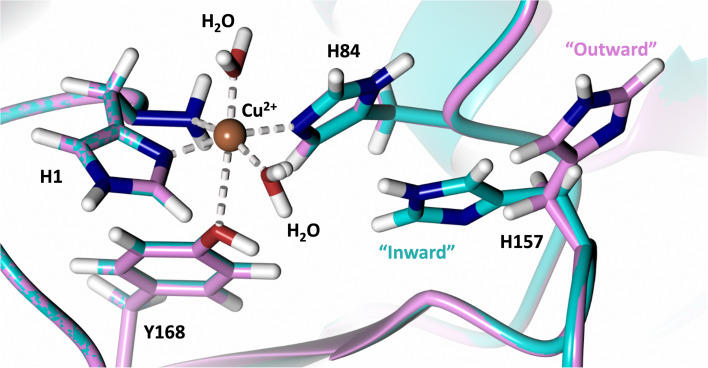
Second shell His157 conformations from VM2 mining minima free energy calculations. For *Nc*LPMO9D with its active site exposed to solvent His157 adopts an “inward” (cyan) and “outward” (pink) conformation. The inward and outward conformations are separated by a 0.11 kcal mol^−1^. H atoms displayed in white.

When applied to *Nc*LPMO9D with its active site exposed to solvent (*i.e.* the monomeric structure), the VM2 method, which performs extensive molecular conformational searching and provides a total free energy *via* a Boltzmann weighted ensemble of the resultant low energy conformers, predicts that both “inward” and “outward” His157 confirmations are populated (a representative structure from these calculations with His157 in the outward conformation was used as the starting model of the MD simulations previously described). Focusing only on the two lowest energy conformers found by VM2 (Fig. 12), the first conformer is “inward” and the second conformer is “outward” with a free energy difference of only 0.11 kcal mol^−1^ between them. Furthermore, summing the probabilities of each type of conformation populated, so that 99.0% of the total population is included, His157 has a predicted 3 : 1 “inward” to “outward” probability distribution. In contrast, when applied to the *Nc*LPMO9D dimer, as per the crystal structure (Fig. 1A), VM2 finds only conformations where His157 remains fully “inward”. This is despite VM2's ability to drive molecular conformations over large energy barriers, as well as a free energy window set at 10 kcal mol^−1^ for retaining minima found by the conformational search. This result strongly supports preclusion of movement of His157 to an “outward” conformation due to the barricaded active site present in the crystal structure. An estimate of the inward/outward conversion barrier in the monomer was obtained by generation of an interpolated reaction path using the Morph Conformations, with corkscrew interpolation, capability in UCSF Chimera^59,76^ between the lowest free energy “inward” and “outward” conformations found by VM2. For each reaction path structure, a constrained conformational search was carried out, providing an energy path for the conversion process. These calculations suggest that the barrier is ∼11.0 kcal mol^−1^ from the inward to outward conformation, and ∼12.1 kcal mol^−1^ from the outward to the inward conformation. More details are available in the ESI – see Table S6 and Fig. S1.†

We conclude, then, that the steric obstruction resulting from crystal packing of the dimer has significantly limited the conformational flexibility of His157 and suggest that the likely mobility of this residue in the monomer, which has a solvent exposed active site, may facilitate its protonation and serve a functional role. Taken together with the crystal structure active site occlusion, these factors may explain the single N_ε_-protonation of His157 observed even at acidic conditions. However, the single protonation of His157 solely at the N_ε_ position in our crystal structures does not rule out formation of the doubly protonated second shell histidine residue under similar acidic *operando* conditions where the active site would not be barricaded by crystal packing.

## Conclusion

The neutron structure presented here provides a full-atom snapshot of the early catalytic intermediates in the LPMO reaction pathway. Our results provide the first direct observation of a cryo-trapped protonated copper–dioxygen species at the LPMO active site. The unique occluded active site environment found in the crystal structure presented here has resulted in stabilization of an activated dioxygen species at the *Nc*LPMO9D active site with joint occupancy as a superoxo intermediate and a hydroperoxo intermediate. A second-shell histidine was further observed in a single N_ε_-protonation state and in the imidazolate form, leading us to postulate that it plays a role in the early stages of dioxygen intermediate protonation to form an end-on coordinated hydroperoxo species, as observed in small molecule copper complexes.^49^ The unique barricaded environment provided by the binding-face to binding-face crystal packing in our structure facilitated trapping of the superoxo/hydroperoxo active site intermediate and identification of His157 as the proton donor. While it is not generally established which reactive intermediate is involved in HAA, the observed hydroperoxo species may perform substrate hydrogen abstraction or undergo reduction and protonation to form a potential Cu oxyl species responsible for substrate hydrogen abstraction in the LPMO reaction cycle. Given the absence of substrate in the current structure the peroxo species may, however, constitute an intermediate in the formation of H_2_O_2_ in a non-productive LPMO catalytic pathway. Although unusual, a neutral histidine functioning as a general acid has been mechanistically proposed for the His95 residue in triose phosphate isomerase and the His98 residue in methylglyoxal synthase.^77–79^ It remains to be determined whether the role of the second shell His157 in protonation of the activated dioxygen species prevails *operando* when *Nc*LPMO9D is solvent-exposed and intermittently binds substrate. The computational studies presented here further describe novel flexibility of LPMO second shell His residues. The flexibility of *Mt*PMO3* His161, as well as that observed for *Nc*LPMO9D His157 to a lesser extent, may serve a functional role in catalysis that must be further explored.

## Experimental and computational methods section

A detailed account of the experimental procedure, data collection and refinement strategy has been described.^80^ Provided here are the experimental details in brief as well as descriptions of the computational methods employed.

### Protein expression and crystallization

*Neurospora crassa* LPMO9D (*Nc*PMO-2) was heterologously expressed in the *Pichia pastoris* SuperMan_5_ strain and purified and crystallized as described by O'Dell *et al.* previously^43,44^ Crystal quality was assessed on beamline CG4-D, the IMAGINE instrument at the High Flux Isotope Reactor at Oak Ridge National Laboratory.^81^

### Crystal ascorbate soak and data collection

To reduce the copper active site, a large crystal was harvested and placed in a deuterated buffer containing 100 mM HEPES pD 6.0 (pH 5.6), 25% PEG 3350 and 100 mM ascorbic acid pD 6.0 (pH 5.6) at room temperature. The crystal was incubated for two hours after which it was flash-frozen in liquid nitrogen using the deuterated buffer supplemented with 25% glycerol as cryoprotectant. Time-of-flight neutron diffraction data were collected on beamline 11B, the Macromolecular Neutron Diffractometer (MaNDi) at the Spallation Neutron Source (SNS) at Oak Ridge National Laboratory.^82^

### Crystal low pH vapor exchange data collection

To obtain low pH conditions in the LPMO crystal, a large crystal was harvested and mounted in a quartz capillary to which a deuterated low pH buffer was added to facilitate vapor exchange in three stages.^83,84^ The first vapor exchange was performed in 22% PEG 3350 and 100 mM sodium acetate pD 5.6 (pH 5.2) for three days, followed by 22% PEG 3350 and 100 mM sodium acetate pD 5.2 (pH 4.8) for six days and 22% PEG 3350 and 100 mM sodium acetate pD 4.8 (pH 4.4) for 16 days. Time-of-flight neutron diffraction data were collected on beamline 11B, the Macromolecular Neutron Diffractometer (MaNDi) at the Spallation Neutron Source (SNS) at Oak Ridge National Laboratory.^82^ Following this, an X-ray dataset was collected on the same crystal at 298 K using a copper rotating-anode home source.

### Crystal low pH direct soak and data collection

LPMO crystals were directly exposed to acidic conditions by soaking crystals in low pH buffer. A crystal was transferred to a buffer composed of 22% PEG 3350, 100 mM and 100 mM sodium acetate with sequential incubation at pH 5.6, pH 5.2 and finally pH 4.8 for ten minutes each. Crystals were subsequently flash-frozen in liquid nitrogen with the pH 4.8 acidic buffer supplemented with 25% glycerol used as a cryoprotectant. Data were collected at 100 K using a copper rotating-anode home source.

### Model refinement

X-ray data were indexed, integrated and scaled with CrysAlisPRO and the CCP4 suite.^85,86^ Neutron diffraction data were reduced using the Mantid data analysis and visualization package.^87^ The time-of-flight diffraction data were integrated using three-dimensional profile fitting of the Bragg peaks.^88^ Structure solution and refinement of the X-ray diffraction data of the low pH structures as well the neutron diffraction data of the ascorbate-soaked structure were performed by utilizing the PHENIX software suite with manual model building to fit density maps performed in Coot.^89–91^ Dioxygen species were modelled using restrained bond length refinements.

### Electronic structure (density functional theory) calculations

The active site models used for DFT calculation were derived by extracting coordinates from the resting state X-ray structure (PDB 5TKG, O'Dell *et al.*^43^) including residues His1, His84, His157, Gln166 and Tyr168 along with the active site copper(ii) ion, pre-bound molecular O2 and the axial and equatorial water molecules. To reduce the total number of atoms in these models, the main chain of residues His84, His157, Gln166 and Tyr168 were truncated at C_β_ which was modeled as a methyl group. DFT calculations were performed with Gaussian 09 using the B3LYP functional with the 6-31G** basis set applied to all atoms. The model was implicitly solvated using the polarizable continuum model as implemented in Gaussian 09 with a dielectric constant of 4.24 (scrf keyword solvent = DiethylEther). For geometry optimization, the coordinates of one heavy atom per residue (as performed previously in DFT calculation of *Nc*LPMO9D in O'Dell *et al.*^43^) were constrained to their starting values to maintain the relative conformation imposed by the protein backbone.

### MD simulations

Molecular dynamics (MD) simulations were performed for LPMO in explicit water solvent, using a protocol developed in our lab.^92^ Model preparation and simulations were performed using the AMBER v16 suite of programs for biomolecular simulations.^93^ AMBER's ff14SB force-fields were used for all simulations.^94^ MD simulations were performed using NVIDIA graphical processing units (GPUs) and AMBER's pmemd.cuda simulation engine using our lab protocols published previously.^95,96^

A total of 4 separate simulations were performed for LPMO in the monomeric form. The monomer form was used to avoid crystal packing artifacts. The first MD simulation was based on the structure determined in this study with His157 in the inward conformation, a second MD simulation was started with His157 in outward conformation obtained from VM2 calculations – see below. The third MD simulation was based on a related LPMO structure with PDB code 5UFW (first protomer only). To rule out any difference in results between the monomeric and dimeric forms, a fourth MD simulation was performed with LPMO in dimeric form based on the structure reported here. (The results of the dimeric complexes were similar to the monomeric form and were excluded from further analysis).

The missing hydrogen atoms were added by AMBER's tleap program. After processing the coordinates of the protein and substrate, all systems were neutralized by addition of counter-ions and the resulting system were solvated in a rectangular box of SPC/E water, with a 10 Å minimum distance between the protein and the edge of the periodic box. The prepared systems were equilibrated using a protocol described previously.^96^ To keep Cu in the active-site for each protomer, 6 distance restraints (5.0 kcal mol^−1^ A^−2^) were applied between the following atoms: Cu-His1_N_, Cu-His1_ND1_, Cu-His84_NE1_, Cu-168Tyr_OH_, and Cu with the two crystallographic water oxygen atoms. For 5UFW based system: Cu-His1_N_, Cu-His1_ND1_, Cu-His75_NE1_, Cu-169Tyr_OH_, and Cu with the two crystallographic water oxygen atoms.

The equilibrated systems were then used to run 1.0 μs of production MD under constant energy conditions (NVE ensemble) and the 6 distance restraints per protomer. The use of the NVE ensemble is preferred as it offers better computational stability and performance.^97^ The production simulations were performed at a temperature of 300 K. As the NVE ensemble was used for production runs, these values correspond to the initial temperature at start of simulations. A temperature adjusting thermostat was not used in the simulations; over the course of 1.0 μs simulations the temperature fluctuated close to 300 K with RMS fluctuations between 2 and 4 K, which is typical for well equilibrated systems. A total of 1000 conformational snapshots (stored every 1000 ps) were collected for each system and were used for analysis. Structures were visualized using PyMOL.

#### RMSF_10_ calculations

For dynamical analysis, backbone (C_α_) and all-atom flexibility of simulation trajectories was determined from the root mean square fluctuation (RMSF), computed by aggregating the magnitude of displacement eigenmodes computed using the quasi-harmonic analysis (QHA) in the ptraj analysis module in AMBER. As described previously,^98,99^ only the top 10 QHA modes (RMSF_10_) were used in the analysis to focus on the principal dynamics or long time-scale functionally relevant fluctuations in the proteins. 1000 conformational snapshots collected from MD were used for this analysis.

### VM2 calculations

VM2 is an implementation of the second-generation Mining Minima (M2) method originally developed by Gilson *et al.*^100–105^ It is a free energy calculation method, which combines extensive molecular conformational searching^71,106^ with a rigorous statistical mechanics approach.^100^ Molecular configuration integrals over all space are closely approximated in a tractable manner by summation of local configuration integrals associated with the low energy minima of the system.^107,108^ VM2's search algorithm can drive molecular conformations over high-energy barriers, *via* a mode-distort-minimize procedure, to find relevant minima even when they structurally diverse. As such, VM2 is well suited for exploration of thermally accessible conformations of His157 in monomeric and dimeric NcLPMO9D, respectively, as carried out in this study.

We applied molecular mechanics (MM) based VM2, taking the AMBER ff14SB parameter files (prmtop *etc.*) generated by the setup for MD simulations (see above) and converting them to formats readable by the VM2 software. It should be noted that for computational efficiency, VM2 includes solvation effects through continuum models (see below for details), so only the water molecules coordinating directly with active site copper centers were explicitly included. To further manage computational expense, and to focus on the region of interest, not all protein atoms were included in the VM2 calculation. Rather, a ‘cutout’ approach was taken, where all residues containing an atom within 14 Å of the His157 alpha carbon (CA) were present in the energy model, and of these all atoms within 12 Å of His157 CA were set as mobile. For the monomer, this resulted in 904 atoms present in the calculation, with 476 of these mobile; for the dimer, this resulted in 1256 atoms present, with 600 of these mobile. To maintain the basic arrangement of the active site copper first-shell coordination residues (histidine brace, tyrosine, and two water molecules) during aggressive conformational searching, a flat-bottomed energy well restraint potential was applied to their coordinating atoms, as well as the central copper atom itself.

VM2 is an iterative method,^71^ with each iteration comprising a conformational search, with removal of any repeat conformers produced, calculation of the configuration integrals at 300 K for the resulting set of new conformers,^107,108^ addition of this set of conformers to the pool already established, and then determination of the total free energy by summation of the local configuration integrals of all conformers (minima) currently in the pool. Once the conformational search no longer finds new low energy conformers the total free energy stops changing between iterations. The calculations in this study were deemed converged when the absolute value of the free energy change between iterations was less than 0.01 kcal mol^−1^. The conformational search carried out during each VM2 iteration included 1200 mode-distort-minimize procedures. Molecular distortions were from atom displacements along single-modes produced by diagonalization of the Hessian matrix in torsional space and, in addition, atom displacements based on random combinations of pairs of these modes.^71,106^ For energy-derivative involved steps, *e.g.* the mode-distort-minimize procedure and the Hessian (energy 2nd-derivative) calculation, required for configurational entropy terms, solvation energy was included using the generalized Born (GB) continuum model.^109,110^ The Poisson–Boltzmann Surface Area (PBSA) method^111^ was applied to provide a more accurate single-point solvation energy correction at GB determined geometries. On completion, VM2 calculations output the total free energy, and in addition the relative free energy of each individual conformer along with its Boltzmann distribution probability. Also outputted are PDB and mol2 formatted files containing all protein conformers produced within 10 kcal mol^−1^ of the lowest energy conformer, up to a maximum of 1000, allowing for structural visualization and analysis.

## Data availability

Crystallographic data for *Nc*LPMO9D at low pH (X-ray structure), ascorbate soaked (neutron structure), low pH vapor exchange (joint X-ray/neutron structure) have been deposited at the Protein Data Bank under PDB IDs 7T5C, 7T5D and 7T5E, respectively, and can be obtained from https://doi.org/10.2210/pdb7t5c/pdb, https://doi.org/10.2210/pdb7t5d/pdb and https://doi.org/10.2210/pdb7t5e/pdb. Molecular dynamics (MD) trajectory data for this paper are available at the Data Archiving and Networked Services (DANS) at https://doi.org/10.17026/dans-zkr-pyrc. Further datasets supporting this article have been uploaded as part of the ESI.†

## Author contributions

GCS, WBO and FM conceived the project. WBO produced and crystallized the protein. GS and FM collected the neutron and X-ray diffraction data. GCS refined the structures. SPW and PKA performed the DFT calculations, free energy calculations and MD simulations. GCS wrote the original draft, and all authors edited and reviewed the paper.

## Conflicts of interest

S. P. W. declares an equity interest in VeraChem LLC, which develops the VM2 free energy software package. The remaining authors declare no conflict of interest.

## Supplementary Material

SC-013-D2SC05031E-s001

SC-013-D2SC05031E-s002

## References

[cit1] Meier K. K., Jones S. M., Kaper T., Hansson H., Koetsier M. J., Karkehabadi S., Solomon E. I., Sandgren M., Kelemen B. (2018). Chem. Rev..

[cit2] Levasseur A., Drula E., Lombard V., Coutinho P. M., Henrissat B. (2013). Biotechnol. Biofuels.

[cit3] Quinlan R. J., Sweeney M. D., Lo Leggio L., Otten H., Poulsen J. C. N., Johansen K. S., Krogh K. B. R. M., Jørgensen C. I., Tovborg M., Anthonsen A., Tryfona T., Walter C. P., Dupree P., Xu F., Davies G. J., Walton P. H. (2011). Proc. Natl. Acad. Sci. U. S. A..

[cit4] Gregory R. C., Hemsworth G. R., Turkenburg J. P., Hart S. J., Walton P. H., Davies G. J. (2016). Dalton Trans..

[cit5] Span E. A., Marletta M. A. (2015). Curr. Opin. Struct. Biol..

[cit6] Gudmundsson M., Kim S., Wu M., Ishida T., Momeni M. H., Vaaje-Kolstad G., Lundberg D., Royant A., Ståhlberg J., Eijsink V. G. H., Beckham G. T., Sandgren M. (2014). J. Biol. Chem..

[cit7] Vaaje-Kolstad G., Westereng B., Horn S. J., Liu Z., Zhai H., Sørlie M., Eijsink V. G. H. (2010). Science.

[cit8] Harris P. V., Welner D., McFarland K. C., Re E., Navarro Poulsen J. C., Brown K., Salbo R., Ding H., Vlasenko E., Merino S., Xu F., Cherry J., Larsen S., Lo Leggio L. (2010). Biochemistry.

[cit9] Phillips C. M., Beeson W. T., Cate J. H., Marletta M. A. (2011). ACS Chem. Biol..

[cit10] Bissaro B., Røhr Å. K., Müller G., Chylenski P., Skaugen M., Forsberg Z., Horn S. J., Vaaje-Kolstad G., Eijsink V. G. H. (2017). Nat. Chem. Biol..

[cit11] Filandr F., Man P., Halada P., Chang H., Ludwig R., Kracher D. (2020). Biotechnol. Biofuels.

[cit12] Stepnov A. A., Forsberg Z., Sørlie M., Nguyen G. S., Wentzel A., Røhr Å. K., Eijsink V. G. H. (2021). Biotechnol. Biofuels.

[cit13] Beeson W. T., Phillips C. M., Cate J. H. D., Marletta M. A. (2012). J. Am. Chem. Soc..

[cit14] Beeson W. T., Vu V. V., Span E. A., Phillips C. M., Marletta M. A. (2015). Annu. Rev. Biochem..

[cit15] Li X., Beeson W. T., Phillips C. M., Marletta M. A., Cate J. H. D. (2012). Structure.

[cit16] Kjaergaard C. H., Qayyum M. F., Wong S. D., Xu F., Hemsworth G. R., Walton D. J., Young N. A., Davies G. J., Walton P. H., Johansen K. S., Hodgson K. O., Hedman B., Solomon E. I. (2014). Proc. Natl. Acad. Sci. U. S. A..

[cit17] Hangasky J. A., Detomasi T. C., Marletta M. A. (2019). Trends Chem..

[cit18] Chylenski P., Bissaro B., Sørlie M., Røhr Å. K., Várnai A., Horn S. J., Eijsink V. G. H. (2019). ACS Catal..

[cit19] Peterson R. L., Himes R. A., Kotani H., Suenobu T., Tian L., Siegler M. A., Solomon E. I., Fukuzumi S., Karlin K. D. (2011). J. Am. Chem. Soc..

[cit20] Mann S. I., Heinisch T., Ward T. R., Borovik A. S. (2017). J. Am. Chem. Soc..

[cit21] Trammell R., See Y. Y., Herrmann A. T., Xie N., Díaz D. E., Siegler M. A., Baran P. S., Garcia-Bosch I. (2017). J. Org. Chem..

[cit22] Garcia-Bosch I., Siegler M. A. (2016). Angew. Chem., Int. Ed..

[cit23] Bertini L., Breglia R., Lambrughi M., Fantucci P., De Gioia L., Borsari M., Sola M., Bortolotti C. A., Bruschi M. (2018). Inorg. Chem..

[cit24] Donoghue P. J., Tehranchi J., Cramer C. J., Sarangi R., Solomon E. I., Tolman W. B. (2011). J. Am. Chem. Soc..

[cit25] Dhar D., Yee G. M., Spaeth A. D., Boyce D. W., Zhang H., Dereli B., Cramer C. J., Tolman W. B. (2016). J. Am. Chem. Soc..

[cit26] Schröder D., Holthausen M. C., Schwarz H. (2004). J. Phys. Chem. B.

[cit27] Yoshizawa K., Kihara N., Kamachi T., Shiota Y. (2006). Inorg. Chem..

[cit28] Dietl N., Van Der Linde C., Schlangen M., Beyer M. K., Schwarz H. (2011). Angew. Chem., Int. Ed..

[cit29] Kim S., Ståhlberg J., Sandgren M., Paton R. S., Beckham G. T. (2014). Proc. Natl. Acad. Sci. U. S. A..

[cit30] Hedegård E. D., Ryde U. (2017). J. Biol. Inorg Chem..

[cit31] Hedegård E. D., Ryde U. (2018). Chem. Sci..

[cit32] Span E. A., Suess D. L. M., Deller M. C., Britt R. D., Marletta M. A. (2017). ACS Chem. Biol..

[cit33] Caldararu O., Oksanen E., Ryde U., Hedegård E. D. (2019). Chem. Sci..

[cit34] Hedegård E. D., Ryde U. (2018). Chem. Sci..

[cit35] Wang B., Walton P. H., Rovira C. (2019). ACS Catal..

[cit36] Schröder G. C., O'Dell W. B., Myles D. A. A., Kovalevsky A., Meilleur F. (2018). Acta Crystallogr., Sect. D: Struct. Biol..

[cit37] O'Dell W. B., Bodenheimer A. M., Meilleur F. (2016). Arch. Biochem. Biophys..

[cit38] Schröder G. C., Meilleur F. (2021). Acta Crystallogr., Sect. D: Struct. Biol..

[cit39] Moody P. C. E., Raven E. L. (2018). Acc. Chem. Res..

[cit40] Kwon H. , Schrader T. E., Ostermann A., Blakeley M. P., Raven E. L. and Moody P. C. E., in Neutron Crystallography in Structural Biology, Elsevier Inc., 1st edn, 2020, vol. 634, pp. 379–389 10.1016/bs.mie.2020.01.010 32093841

[cit41] Casadei C. M., Gumiero A., Metcalfe C. L., Murphy E. J., Basran J., Concilio M. G., Teixeira S. C. M., Schrader T. E., Fielding A. J., Ostermann A., Blakeley M. P., Raven E. L., Moody P. C. E. (2014). Science.

[cit42] Kwon H., Basran J., Casadei C. M., Fielding A. J., Schrader T. E., Ostermann A., Devos J. M., Aller P., Blakeley M. P., Moody P. C. E., Raven E. L. (2016). Nat. Commun..

[cit43] O'Dell W. B., Agarwal P. K., Meilleur F. (2017). Angew. Chem., Int. Ed..

[cit44] O'Dell W. B., Swartz P. D., Weiss K. L., Meilleur F. (2017). Acta Crystallogr., Sect. F: Struct. Biol. Commun..

[cit45] Karkehabadi S., Hansson H., Kim S., Piens K., Mitchinson C., Sandgren M. (2008). J. Mol. Biol..

[cit46] Eibinger M., Sattelkow J., Ganner T., Plank H., Nidetzky B. (2017). Nat. Commun..

[cit47] Frandsen K. E. H., Simmons T. J., Dupree P., Poulsen J. C. N., Hemsworth G. R., Ciano L., Johnston E. M., Tovborg M., Johansen K. S., Von Freiesleben P., Marmuse L., Fort S., Cottaz S., Driguez H., Henrissat B., Lenfant N., Tuna F., Baldansuren A., Davies G. J., Lo Leggio L., Walton P. H. (2016). Nat. Chem. Biol..

[cit48] Bacik J. P., Mekasha S., Forsberg Z., Kovalevsky A. Y., Vaaje-Kolstad G., Eijsink V. G. H., Nix J. C., Coates L., Cuneo M. J., Unkefer C. J., Chen J. C. H. (2017). Biochemistry.

[cit49] Davydov R., Herzog A. E., Jodts R. J., Karlin K. D., Hoffman B. M. (2022). J. Am. Chem. Soc..

[cit50] Isaksen T., Westereng B., Aachmann F. L., Agger J. W., Kracher D., Kittl R., Ludwig R., Haltrich D., Eijsink V. G. H., Horn S. J. (2014). J. Biol. Chem..

[cit51] Barth A. (2000). Prog. Biophys. Mol. Biol..

[cit52] Quist D. A., Ehudin M. A., Schaefer A. W., Schneider G. L., Solomon E. I., Karlin K. D. (2019). J. Am. Chem. Soc..

[cit53] Neisen B. D., Gagnon N. L., Dhar D., Spaeth A. D., Tolman W. B. (2017). J. Am. Chem. Soc..

[cit54] Kim B., Jeong D., Ohta T., Cho J. (2019). Commun. Chem..

[cit55] Elwell C. E., Gagnon N. L., Neisen B. D., Dhar D., Spaeth A. D., Yee G. M., Tolman W. B. (2017). Chem. Rev..

[cit56] Westereng B., Cannella D., Wittrup Agger J., Jørgensen H., Larsen Andersen M., Eijsink V. G. H., Felby C. (2015). Sci. Rep..

[cit57] Courtade G., Wimmer R., Røhr Å. K., Preims M., Felice A. K. G., Dimarogona M., Vaaje-Kolstad G., Sørlie M., Sandgren M., Ludwig R., Eijsink V. G. H., Aachmann F. L. (2016). Proc. Natl. Acad. Sci. U. S. A..

[cit58] Larsson E. D., Dong G., Veryazov V., Ryde U., Hedegård E. D. (2020). Dalton Trans..

[cit59] Roos B. O., Taylor P. R., Sigbahn P. E. M. (1980). Chem. Phys..

[cit60] Ciano L., Davies G. J., Tolman W. B., Walton P. H. (2018). Nat. Catal..

[cit61] Dhar D., Tolman W. B. (2015). J. Am. Chem. Soc..

[cit62] Paradisi A., Johnston E. M., Tovborg M., Nicoll C. R., Ciano L., Dowle A., Mcmaster J., Hancock Y., Davies G. J., Walton P. H. (2019). J. Am. Chem. Soc..

[cit63] Singh R. K., Blossom B. M., Russo D. A., Singh R., Weihe H., Andersen N. H., Tiwari M. K., Jensen P. E., Felby C., Bjerrum M. J. (2020). Chem.–Eur. J..

[cit64] McEvoy A., Creutzberg J., Singh R. K., Bjerrum M. J., Hedegård E. D. (2021). Chem. Sci..

[cit65] Schülein M. (1997). J. Biotechnol..

[cit66] Boer H., Koivula A. (2003). Eur. J. Biochem..

[cit67] Cragg S. M., Beckham G. T., Bruce N. C., Bugg T. D. H., Distel D. L., Dupree P., Etxabe A. G., Goodell B. S., Jellison J., McGeehan J. E., McQueen-Mason S. J., Schnorr K., Walton P. H., Watts J. E. M., Zimmer M. (2015). Curr. Opin. Chem. Biol..

[cit68] Banerjee S., Muderspach S. J., Tandrup T., Frandsen K. E. H., Singh R. K., Ipsen J. Ø., Hernández-Rollán C., Nørholm M. H. H., Bjerrum M. J., Johansen K. S., Lo Leggio L. (2022). *Biomolecules*.

[cit69] Frandsen K. E. H., Poulsen J. C. N., Tandrup T., Lo Leggio L. (2017). Carbohydr. Res..

[cit70] Ramanathan A., Savol A., Burger V., Chennubhotla C. S., Agarwal P. K. (2014). Acc. Chem. Res..

[cit71] Chen W., Gilson M. K., Webb S. P., Potter M. J. (2010). J. Chem. Theory Comput..

[cit72] Huang Y. M. M., Chen W., Potter M. J., Chang C. E. A. (2012). Biophys. J..

[cit73] You W., Huang Y. m. M., Kizhake S., Natarajan A., Chang C. en A. (2016). PLoS Comput. Biol..

[cit74] Xu P., Sattasathuchana T., Guidez E., Webb S. P., Montgomery K., Yasini H., Pedreira I. F. M., Gordon M. S. (2021). J. Chem. Phys..

[cit75] Chen W., Ren X., Chang C. en A. (2019). ChemMedChem.

[cit76] Pettersen E. F., Goddard T. D., Huang C. C., Couch G. S., Greenblatt D. M., Meng E. C., Ferrin T. E. (2004). J. Comput. Chem..

[cit77] Lodi P. J., Knowles J. R. (1991). Biochemistry.

[cit78] Lennartz C., Schäfer A., Terstegen F., Thiel W. (2002). J. Phys. Chem. B.

[cit79] Marks G. T., Susler M., Harrison D. H. T. (2004). Biochemistry.

[cit80] Schröder G. C., O'Dell W. B., Swartz P. D., Meilleur F. (2021). Acta Crystallogr., Sect. F: Struct. Biol. Commun..

[cit81] Meilleur F. , Kovalevsky A. and Myles D. A. A., in Methods in enzymology, Elsevier Inc., 1st edn, 2020, vol. 634, pp. 69–85 10.1016/bs.mie.2019.11.016 32093843

[cit82] Coates L., Stoica A. D., Hoffmann C., Richards J., Cooper R. (2010). J. Appl. Crystallogr..

[cit83] Wan Q., Parks J. M., Hanson B. L., Fisher S. Z., Ostermann A., Schrader T. E., Graham D. E., Coates L., Langan P., Kovalevsky A. (2015). Proc. Natl. Acad. Sci. U. S. A..

[cit84] Gerlits O., Wymore T., Das A., Shen C. H., Parks J. M., Smith J. C., Weiss K. L., Keen D. A., Blakeley M. P., Louis J. M., Langan P., Weber I. T., Kovalevsky A. (2016). Angew. Chem., Int. Ed..

[cit85] Kubicek C. P. , Fungi and Lignocellulosic Biomass, Wiley-Blackwell, Oxford, UK, 2012

[cit86] Winn M. D., Ballard C. C., Cowtan K. D., Dodson E. J., Emsley P., Evans P. R., Keegan R. M., Krissinel E. B., Leslie A. G. W., McCoy A., McNicholas S. J., Murshudov G. N., Pannu N. S., Potterton E. A., Powell H. R., Read R. J., Vagin A., Wilson K. S. (2011). Acta Crystallogr., Sect. D: Biol. Crystallogr..

[cit87] Arnold O., Bilheux J. C., Borreguero J. M., Buts A., Campbell S. I., Chapon L., Doucet M., Draper N., Ferraz Leal R., Gigg M. A., Lynch V. E., Markvardsen A., Mikkelson D. J., Mikkelson R. L., Miller R., Palmen K., Parker P., Passos G., Perring T. G., Peterson P. F., Ren S., Reuter M. A., Savici A. T., Taylor J. W., Taylor R. J., Tolchenov R., Zhou W., Zikovsky J. (2014). Nucl. Instrum. Methods Phys. Res., Sect. A.

[cit88] Sullivan B., Archibald R., Langan P. S., Dobbek H., Bommer M., McFeeters R. L., Coates L., Wang X., Gallmeier F., Carpenter J. M., Lynch V., Langan P. (2018). Acta Crystallogr., Sect. D: Struct. Biol..

[cit89] Adams P. D., Afonine P. V., Bunkóczi G., Chen V. B., Davis I. W., Echols N., Headd J. J., Hung L.-W., Kapral G. J., Grosse-Kunstleve R. W., McCoy A. J., Moriarty N. W., Oeffner R., Read R. J., Richardson D. C., Richardson J. S., Terwilliger T. C., Zwart P. H. (2010). Acta Crystallogr., Sect. D: Biol. Crystallogr..

[cit90] Emsley P., Lohkamp B., Scott W. G., Cowtan K. (2010). Acta Crystallogr., Sect. D: Biol. Crystallogr..

[cit91] Afonine P. V., Grosse-Kunstleve R. W., Echols N., Headd J. J., Moriarty N. W., Mustyakimov M., Terwilliger T. C., Urzhumtsev A., Zwart P. H., Adams P. D. (2012). Acta Crystallogr., Sect. D: Biol. Crystallogr..

[cit92] Narayanan C., Bernard D. N., Bafna K., Gagné D., Chennubhotla C. S., Doucet N., Agarwal P. K. (2018). Structure.

[cit93] Case D. A. , Berryman J. T., Betz R. M., Cerutti D. S., Cheatham T. E., Darden T. A., Duke R. E., Giese T. J., Gohlke H., Goetz A. W., Homeyer N., Izadi S., Janowski P., Kaus J., Kovalenko A., Lee T. S., LeGrand S., Li P., Luchko T., Luo R., Madej B., Merz K. M., Monard G., Needham P., Nguyen H., Nguyen H. T., Omelyan I., Onufriev A., Roe D. R., Roitberg A., Salomon-Ferrer R., Simmerling C. L., Smith W., Swails J., Walker R. C., Wang J., Wolf R. M., Wu X., York D. M. and Kollman P. A., AMBER 15, University of California, San Francisco, 2015

[cit94] Maier J. A., Martinez C., Kasavajhala K., Wickstrom L., Hauser K. E., Simmerling C. (2015). J. Chem. Theory Comput..

[cit95] Ramanathan A., Agarwal P. K., Kurnikova M., Langmead C. J. (2010). J. Comput. Biol..

[cit96] Duff M. R., Borreguero J. M., Cuneo M. J., Ramanathan A., He J., Kamath G., Chennubhotla S. C., Meilleur F., Howell E. E., Herwig K. W., Myles D. A. A., Agarwal P. K. (2018). Biochemistry.

[cit97] Shukla S., Bafna K., Gullett C., Myles D. A. A., Agarwal P. K., Cuneo M. J. (2018). Biochemistry.

[cit98] Ramanathan A., Agarwal P. K. (2009). J. Phys. Chem. B.

[cit99] Bafna K., Narayanan C., Chakra Chennubhotla S., Doucet N., Agarwal P. K. (2019). PLoS One.

[cit100] Gilson M. K., Given J. A., Bush B. L., McCammon J. A. (1997). Biophys. J..

[cit101] Head M. S., Given J. A., Gilson M. K. (1997). J. Phys. Chem. A.

[cit102] David L., Luo R., Gilson M. K. (2001). J. Comput.-Aided Mol. Des..

[cit103] Kairys V., Gilson M. K. (2002). J. Comput. Chem..

[cit104] Chang C. E., Gilson M. K. (2004). J. Am. Chem. Soc..

[cit105] Chen W., Chang C. E., Gilson M. K. (2004). Biophys. J..

[cit106] Chang C. E., Gilson M. K. (2003). J. Comput. Chem..

[cit107] Chang C. E., Potter M. J., Gilson M. K. (2003). J. Phys. Chem. B.

[cit108] Potter M. J., Gilson M. K. (2002). J. Phys. Chem. A.

[cit109] Qiu D., Shenkin P. S., Hollinger F. P., Still W. C. (1997). J. Phys. Chem. A.

[cit110] Still W. C., Tempczyk A., Hawley R. C., Hendrickson T. (1990). J. Am. Chem. Soc..

[cit111] Luo R., David L., Gilson M. K. (2002). J. Comput. Chem..

