# Supplementary material for: Capture of activated dioxygen intermediates at the copper-active site of a lytic polysaccharide monooxygenase
Source: Chem Sci. 2022 Nov 2;13(45):13303–20. doi: 10.1039/d2sc05031e (PMC9683017; doi:10.1039/d2sc05031e)
Supplement: SC-013-D2SC05031E-s001 [file SC-013-D2SC05031E-s001.pdf]

Electronic Supplementary Material (ESI) for Chemical Science.
This journal is © The Royal Society of Chemistry 2022

Supplementary Information

Capture of activated dioxygen intermediates at the copper-active site of a lytic polysaccharide monooxygenase

Gabriela C. Schröder[1,2], William B. O'Dell[1,2,3], Simon P. Webb[4], Pratul K. Agarwal[5]* and Flora Meilleur[1,2]*

[1]Department of Molecular and Structural Biochemistry, North Carolina State University, Raleigh, NC 27695, USA
[2]Neutron Scattering Division, Oak Ridge National Laboratory, Oak Ridge, TN 37831, USA
[3]Current affiliations: Biomolecular Measurement Division, National Institute of Standards and Technology, Gaithersburg, MD 20899; Biomolecular Labeling Laboratory, Institute for Bioscience and Biotechnology Research, Rockville, MD 20850
[4]VeraChem LLC, 12850 Middlebrook Rd. Ste 205, Germantown, MD 20874-5244, USA
[5]Department of Physiological Sciences and High-Performance Computing Center, Oklahoma State University, Stillwater, OK 74078, USA

Contents

1. **Supplementary Tables**
   Table S1. X-ray and neutron crystallographic statistics
   Table S2. X-ray and neutron model refinement statistics
   Table S3. Copper–ligand distances of the low pH vapor exchange joint-refined neutron structure
   Table S4. Copper–ligand distances of the low pH direct soak X-ray structure
   Table S5. Copper–ligand distances of the ascorbate-treated neutron structure
   Table S6. VM2 calculated energies for a pathway from His157 inward to outward conformation
2. **Supplementary Figures**
   Figure S1. Plot of VM2 generated energy path for His157 inward/outward conversion
   Figure S2. DFT active site model of superoxo species bound with His157 NE2-protonated.
   Figure S3. DFT active site model of hydroperoxyl species bound with His157 deprotonated.
   Figure S4. DFT active site model of hydroperoxo species bound after 1 electron reduction with His157 deprotonated.
   Figure S5. DFT active site model of hydroperoxo species bound after 2 electron reduction with His157 deprotonated.
   Figure S6. X-ray structure of LPMO9D His157 Molecule A after direct exposure to acidic conditions.
   Figure S7. X-ray structure of LPMO9D His157 Molecule B after direct exposure to acidic conditions.

1. Supplementary Tables

Table S1. X-ray and neutron crystallographic data statistics.

**Description**	Low pH vapor	Low pH vapor	Direct low pH	Ascorbate
**Incident Radiation**	X-ray	Neutron	X-ray	Neutron
**Temperature (K)**	298	298	100	100
**Wavelength (Å)**	1.54	2.0-4.0	1.54	2.0-4.0
**Resolution range (Å)**	12.65 - 1.9 (1.97 - 1.9)	14.65 - 2.14 (2.22 - 2.14)	12.61 - 1.5 (1.55- 1.5)	14.79 - 2.4 (2.49 - 2.4)
**Space group**	$P\ 1\ 2_1\ 1$	$P\ 1\ 2_1\ 1$	$P\ 1\ 2_1\ 1$	$P\ 1\ 2_1\ 1$
**Unit cell (a, b, c) (Å) (α, β, γ) (°)**	68.30 42.27 70.41 90 98.47 90	68.30 42.27 70.41 90 98.47 90	67.67 42.21 69.72 90 98.90 90	67.73 42.18 69.76 90 99 90
**Total reflections**	190116 (11672)	75277 (6053)	512918 (27801)	45082 (2985)
**Unique reflections**	30950 (2859)	18963 (1655)	62507 (6159)	14168 (1300)
**Multiplicity**	6.1 (4.1)	4.0 (3.3)	8.2 (4.5)	3.2 (2.3)
**Molecules per ASU**	2	2	2	2
**Completeness (%)**	97.56 (91.75)	84.65 (74.75)	99.78 (99.97)	91.16 (84.86)
**Mean intensity/sigma(intensity)**	15.24 (4.40)	10.01 (3.02)	62.56 (7.14)	7.15 (2.28)
**Wilson B-factor (Å^2)**	17.17	34.32	11.79	31.17
**R_{merge}**	0.1152 (0.299)	0.1532 (0.328)	0.142 (0.2775)	0.1851 (0.2737)
**R_{meas}**	0.1258 (0.3443)	0.1751 (0.3824)	0.1495 (0.3133)	0.2148 (0.3327)
**$CC_{1/2}$**	0.991 (0.88)	0.97 (0.363)	0.992 (0.94)	0.956 (0.297)

Table S2. X-ray and neutron model refinement statistics.

**Description**	Low pH vapor (Joint X-ray)	Low pH vapor (Joint neutron)	Direct low pH (X-ray)	Ascorbate (Neutron)
**Reflections used in refinement**	30948 (2859)	18770 (1655)	62504 (6158)	14167 (1300)
**Reflections used for R-free**	1557 (144)	954 (113)	3110 (332)	710 (60)
R_{work}	0.1276 (0.1649)	0.1446 (0.2167)	0.1556 (0.1784)	0.2282 (0.3010)
R_{free}	0.1822 (0.2606)	0.2137 (0.3044)	0.1822 (0.2291)	0.3092 (0.3595)
**Number of non-hydrogen atoms**	3851		4327	3688
Macromolecules	3382		3481	3212
Ligands	80		69	62
Solvent	389		777	414
Protein residues	446		446	446
**RMS(bonds)**	0.097		0.008	0.095
**RMS(angles)**	1.38		1.15	0.75
**Ramachandran favored (%)**	97.06		96.61	93.44
**Ramachandran allowed (%)**	2.49		2.94	5.88
**Ramachandran outliers (%)**	0.45		0.45	0.68
**Rotamer outliers (%)**	4.50		1.52	1.27
**Clash score**	10.15		3.68	12.13
**Average B-factor**	23.34		16.52	35.37
Macromolecules	21.56		13.76	35.33
Carbohydrate	47.71		31.81	42.91
Copper	18.51		10.77	34.15
Superoxo				34.04
Hydroperoxo				36.50
Solvent	38.86		27.28	35.82

Table S3. Copper–ligand distances and selected atomic displacement factors in the active site of the low pH vapor exchange joint-refined neutron structure.

	**Molecule A**	**Molecule B**
**Distance (Å)**		
Cu–His1-N_{δ}	1.99	1.99
Cu–His1-N_{amino}	2.13	2.03
Cu–His84-N_{ε}	2.00	2.04
Cu–Tyr168-OH	2.67	2.66
Cu–$H_2O_{equatorial}$	2.08	2.03
Cu–H_2O_{axial}	2.34	2.43

Table S4. Copper–ligand distances and selected atomic displacement factors in the active site of the low pH direct soak X-ray structure.

	Molecule A	Molecule B
**Distance (Å)**		
Cu–His1-N_δ	1.98	1.99
Cu–His1-N_{amino}	2.09	2.13
Cu–His84-N_ε	2.02	2.01
Cu–Tyr168-OH	2.68	2.65
Cu–$H_2O_{equatorial}$	2.12	2.11
Cu–H_2O_{axial}	2.42	2.43

Table S5. Copper–ligand distances and selected atomic displacement factors in the active site of the ascorbate-treated neutron structure.

	**Molecule A**	**Molecule B**
**Distance (Å)**		
Cu–His1-N_{δ}	1.97	1.89
Cu–His1-N_{amino}	2.20	2.22
Cu–His84-N_{ε}	1.99	1.90
Cu–Tyr168-OH	2.67	2.64
Cu–$H_2O_{equatorial}$		2.24
Cu–$O1_{hydroperoxo}$	1.98	
O1–$O2_{hydroperoxo}$	1.46	
Cu–$O1_{superoxo}$	1.96	
O1–$O2_{superoxo}$	1.28	

Table S6. VM2 calculated energies (U+W) and relative energies (Δ(U+W)) for a pathway from His157 inward to outward conformation

Transition reaction coordinate	U+W (kcal/mol)	Δ(U+W)
1 (inward)	-1090.31	0.00
2	-1090.30	0.01
3	-1089.88	0.43
4	-1088.23	2.08
5	-1087.65	2.65
6	-1085.91	4.40
7	-1084.72	5.59
8	-1082.92	7.39
9	-1081.27	9.04
10	-1080.12	10.19
11	-1082.34	7.97
12	-1079.35	10.96
13	-1080.37	9.94
14	-1081.67	8.64
15	-1083.91	6.40
16	-1083.58	6.73
17	-1085.32	4.99
18	-1086.47	3.84
19	-1087.15	3.16
20	-1089.43	0.88
21 (outward)	-1091.44	-1.13

U = gas phase energy (AMBER's *ff14SB*), W = solvation energy (GB geometry optimizations with PBSA single-point energy corrections)
His157 CA, CB, CG and CD2 were fixed while conformational searching was applied to all other VM2 'live' atoms.

Methodology for generation of His157 "inward" to "outward" energy path.

Initial morphing conformations. The Morph Conformations capability in UCSF Chimera,[1,2] with rigid corkscrew interpolation, was used to generate an initial set of conformations that morph between the lowest free energy "inward" and "outward" NcLPMO9D monomer conformations produced by VM2. (See the section: *Mining minima (VM2) free energy calculations*, Figure 9, for VM2 NcLPMO9D calculation details.) A total of 21 NcLPMO9D monomer conformers were generated, including conformer 1 corresponding to "inward" His157 and conformer 21 corresponding to "outward" His157.

Relaxed energy path using VM2. Each of the initial morphing conformations, generated via rigid interpolation, was then used to initiate a constrained VM2 conformational search, where the His157 CA, CB, CG and CD2 atoms were fixed in space, but all other atoms in the VM2 live set were allowed to move, generating a reaction path that allowed for relaxation of the NcLPMO9D structure during the His157 inward/outward flip. The lowest energy, i.e., the gas phase plus solvation energy (U+W), conformation found for each of the 21 constrained VM2 conformational searches was taken as a structure on the reaction path. The energies (U+W) and relative energies along this reaction path are reported in Table S1. The details of the forcefield and solvation treatment, as well as protein 'cutout' model, were identical to those used for the original VM2 free energy calculations reported in the main paper. (Note that we do not report VM2 free energies for the reaction path, as currently only U+W is available in VM2 calculations when there are live atoms fixed in space.)

Supplementary Figures

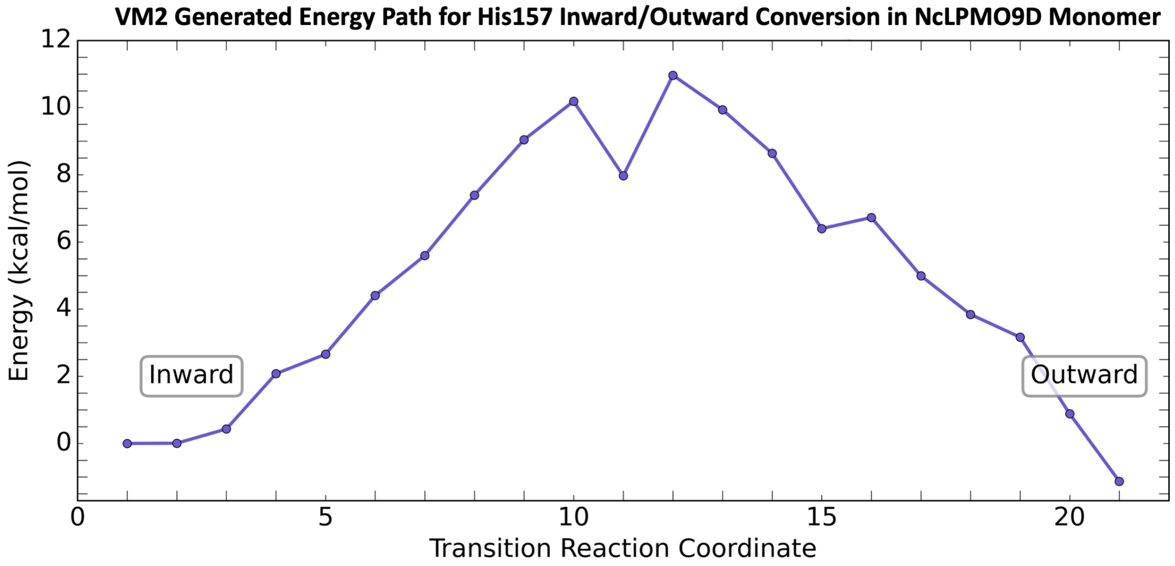

Figure S1. VM2 calculated relative energies, Δ(U+W), for reaction coordinate pathway from His157 inward to outward conformation. See Table S6 and description of methodology for transition reaction coordinate and path energy generation above.

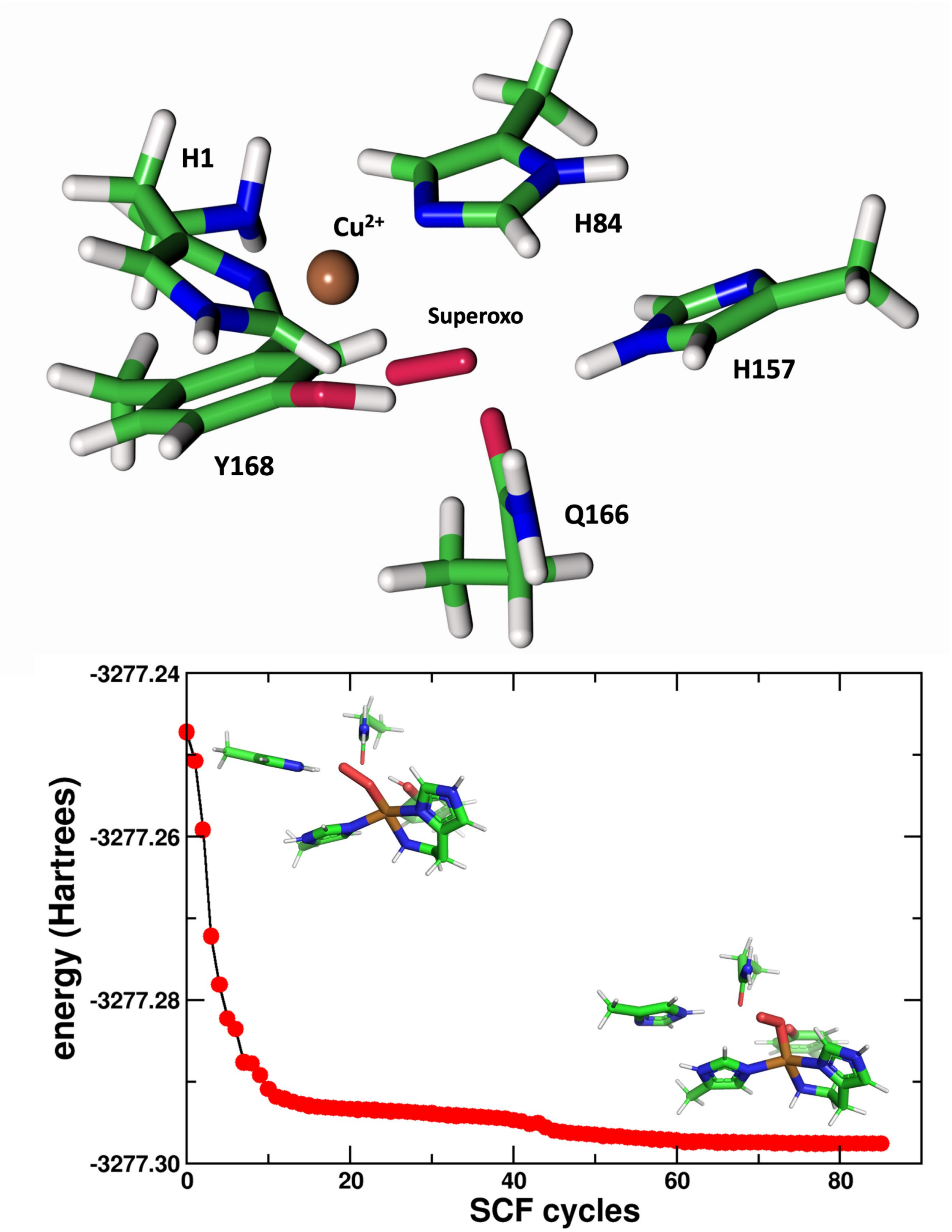

Figure S2. DFT active site model and convergence behavior of superoxo species bound with His157 NE2-protonated. The starting and ending structures are shown as insets.

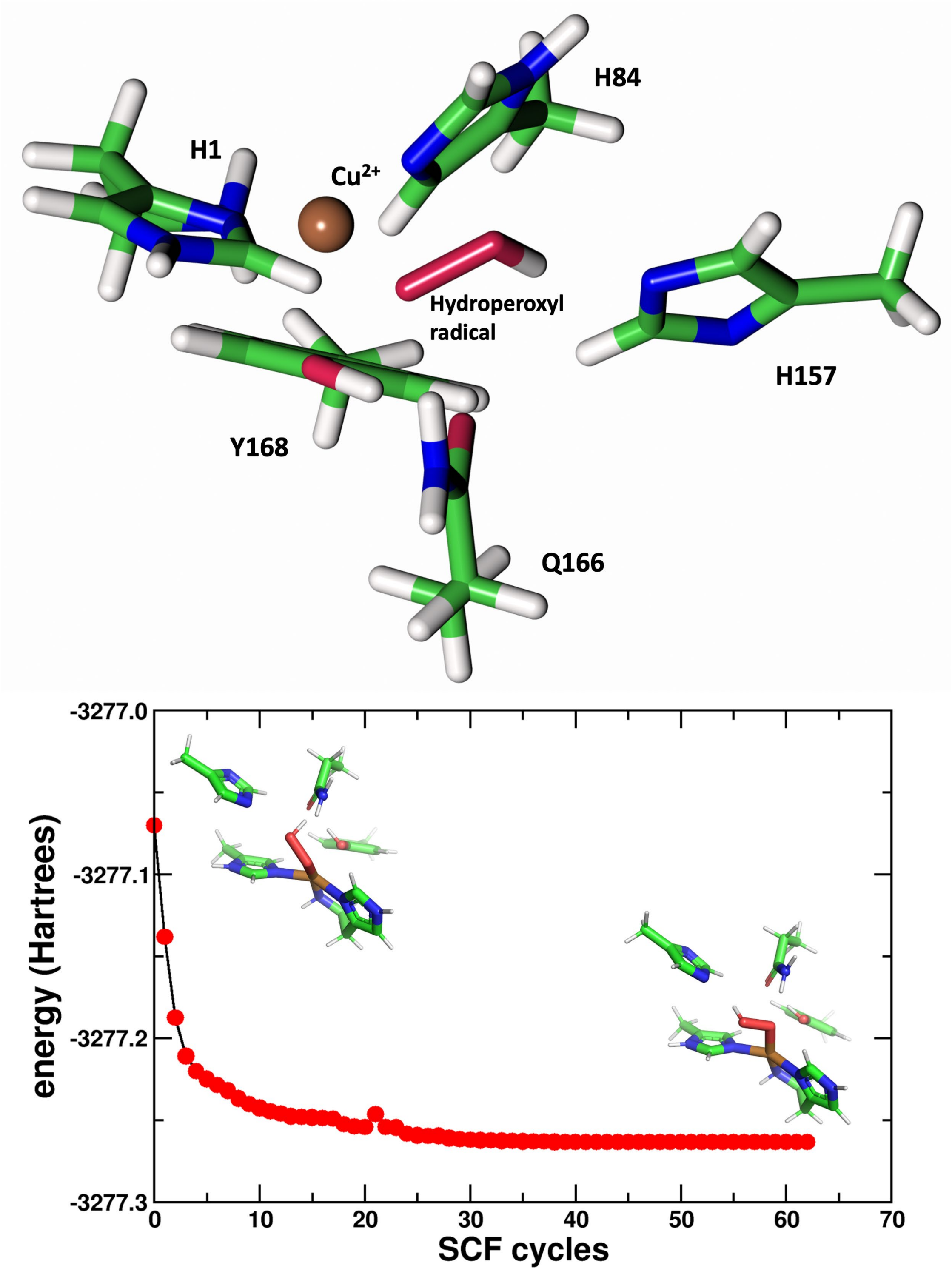

Figure S3. DFT active site model and convergence behavior of hydroperoxyl species bound with His157 deprotonated. The starting and ending structures are shown as insets.

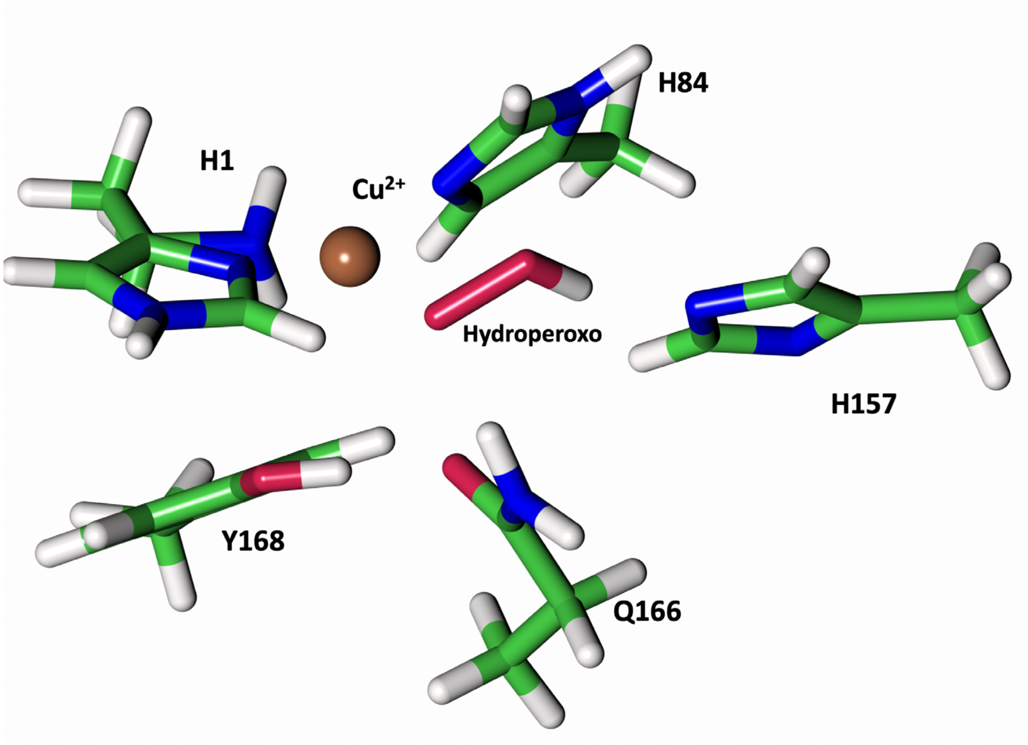

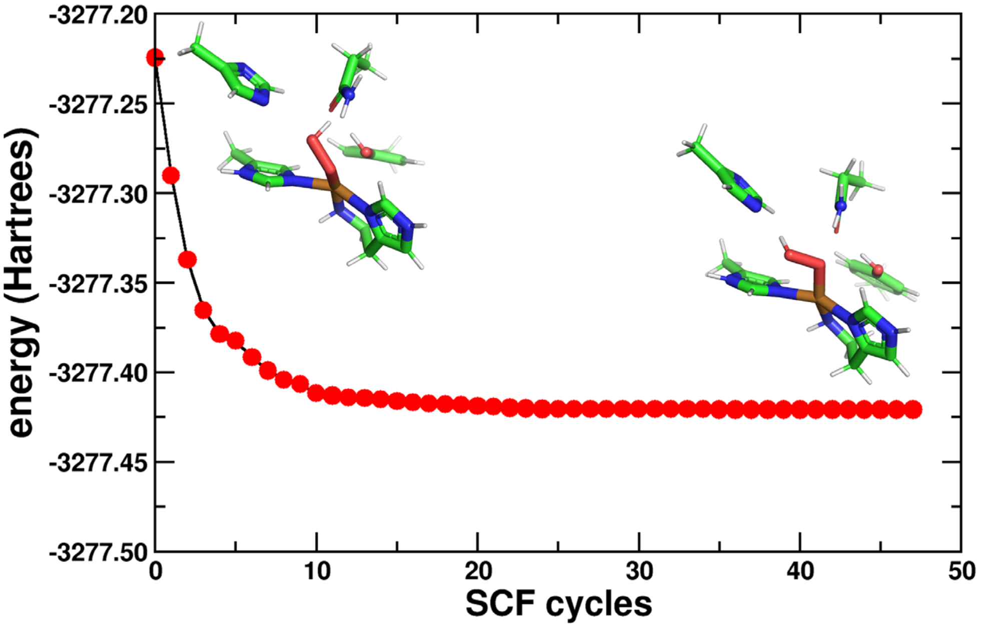

Figure S4. DFT active site model and convergence of hydroperoxo species bound after 1 electron reduction with His157 deprotonated. The starting and ending structures are shown as insets.

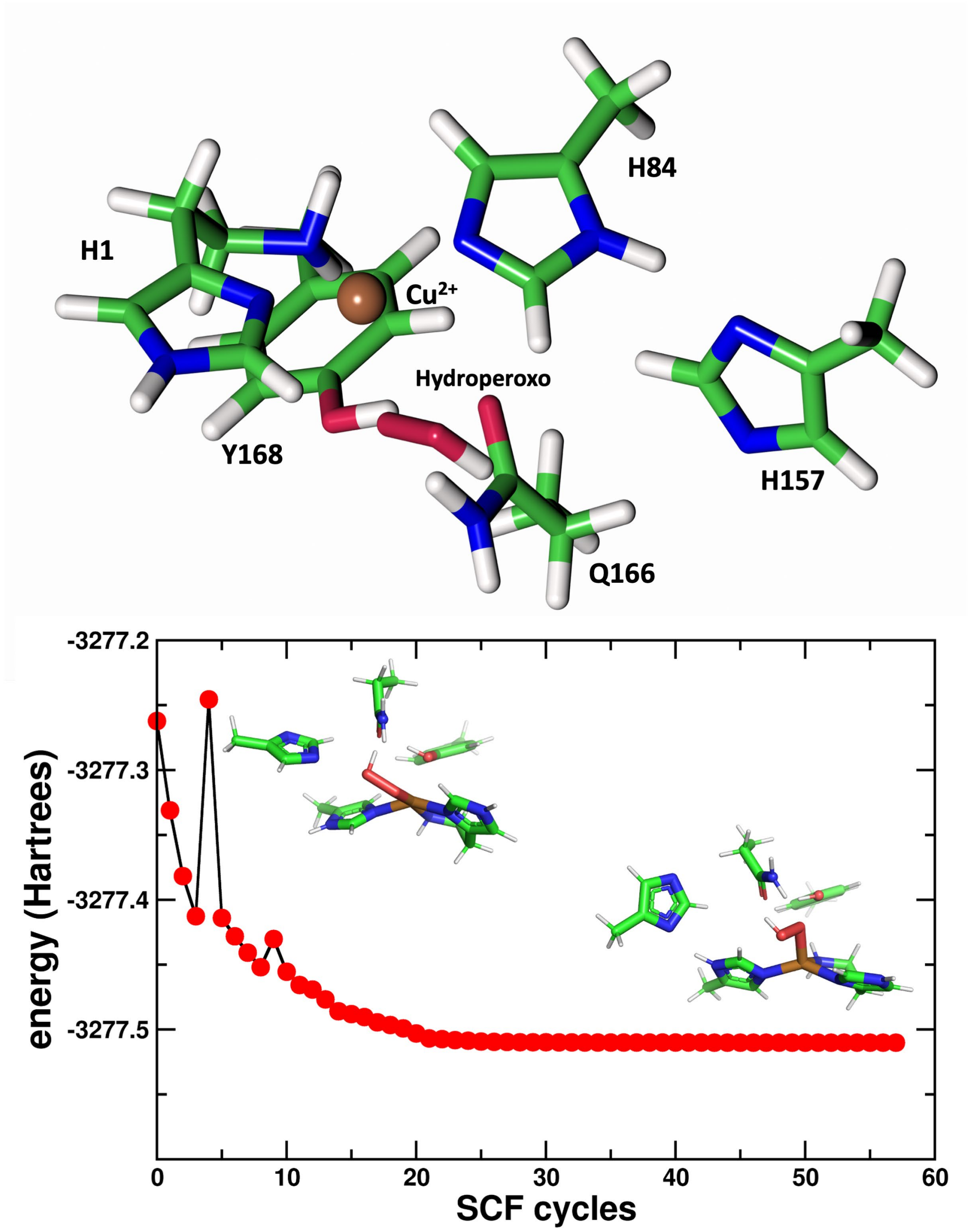

Figure S5. DFT active site model and convergence behavior of hydroperoxo species bound after 2 electron reduction with His157 deprotonated. The starting and ending structures are shown as insets.

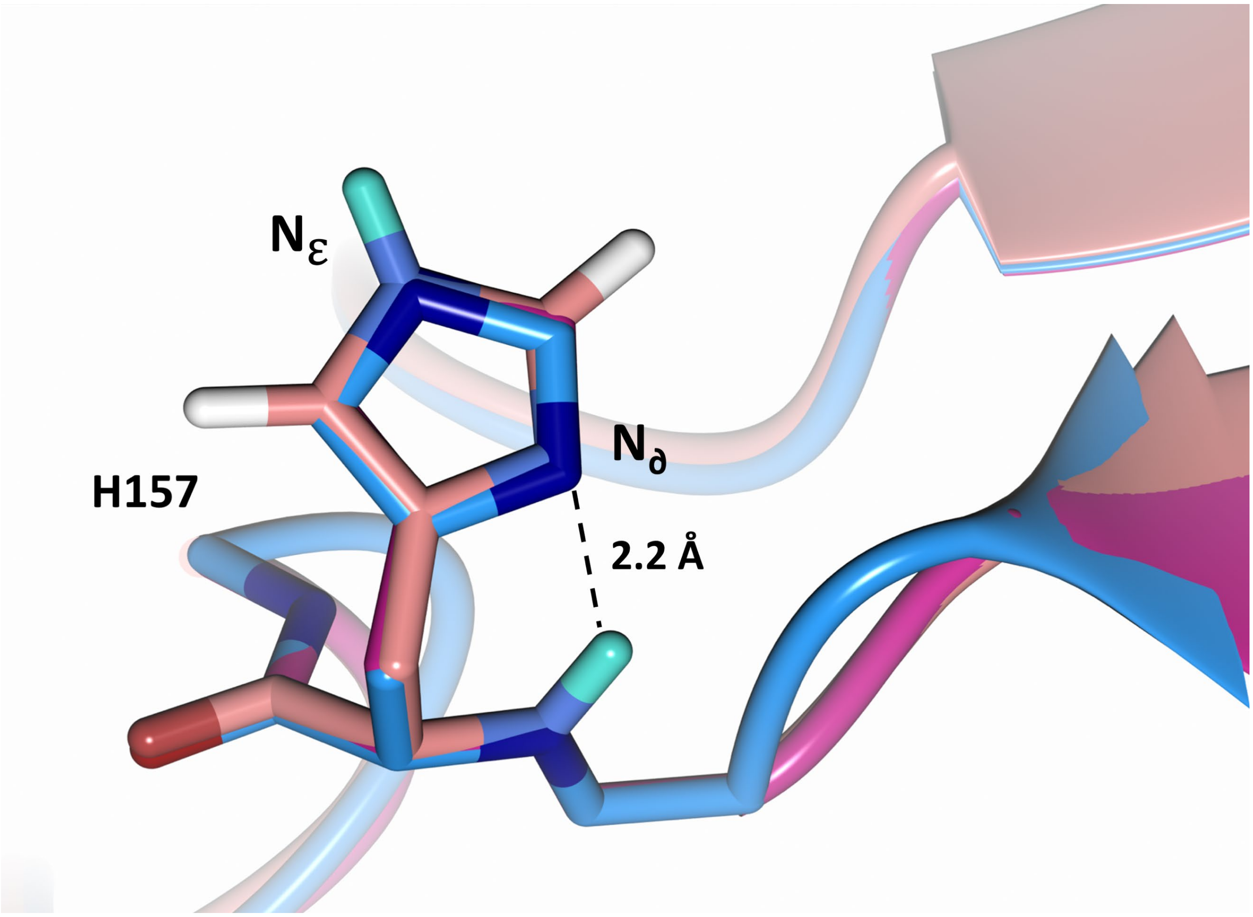

Figure S6. X-ray structure of LPMO9D His157 Molecule A after direct exposure to acidic conditions. Structural alignment of LPMO9D Molecule A neutron diffraction data collected at pH 4.4/pD 4.8 using vapor exchange (coral) with an X-ray diffraction dataset of Molecule A following a direct pH 4.4/pD 4.8 soak (blue) and X-ray diffraction of the resting state of LPMO9D Molecule B (PDB 5TKG) at pH 6.0 (magenta). H atoms displayed in white and D atoms displayed in turquoise.

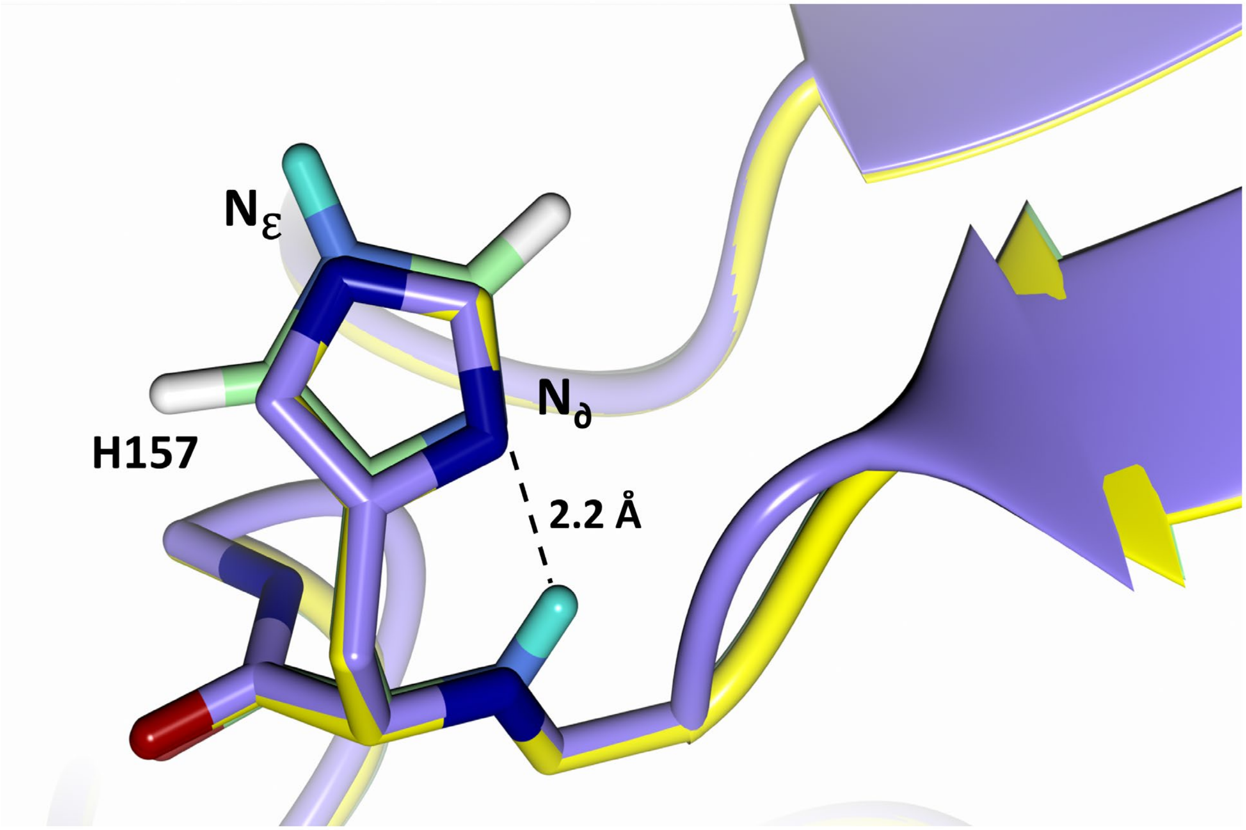

Figure S7. X-ray structure of LPMO9D His157 Molecule B after direct exposure to acidic conditions. Structural alignment of LPMO9D Molecule B neutron diffraction data collected at pH 4.4/pD 4.8 using vapor exchange (light green) with an X-ray diffraction dataset of Molecule B following a direct pH 4.4/pD 4.8 soak (purple) and X-ray diffraction of the resting state of LPMO9D Molecule B (PDB 5TKG) at pH 6.0 (yellow). H atoms displayed in white and D atoms displayed in turquoise.

References

1. Roos, B. O., Taylor, P. R. & Sigbahn, P. E. M. A complete active space SCF method (CASSCF) using a density matrix formulated super-CI approach. *Chem. Phys.* **48**, 157–173 (1980).
2. Pettersen, E. F. *et al.* UCSF Chimera - A visualization system for exploratory research and analysis. *J. Comput. Chem.* **25**, 1605–1612 (2004).
