# Supplementary material for: Capture of activated dioxygen intermediates at the copper-active site of a lytic polysaccharide monooxygenase
Source: Chem Sci. 2022 Nov 2;13(45):13303–20. doi: 10.1039/d2sc05031e (PMC9683017; doi:10.1039/d2sc05031e)
Supplement: SC-013-D2SC05031E-s002 [file SC-013-D2SC05031E-s002.pdf]

Electronic Supplementary Material (ESI) for Chemical Science.
This journal is © The Royal Society of Chemistry 2022

Preliminary Full wwPDB Geometry-Only Validation Report (i)

PRELIMINARY VALIDATION REPORT

Dec 8, 2021 – 01:58 PM EST

Deposition ID : D_1000261513

This is a Preliminary Full wwPDB Geometry-Only Validation Report.

This report is produced by the wwPDB Deposition System during initial deposition but before annotation of the structure.

We welcome your comments at *validation@mail.wwpdb.org*
A user guide is available at
https://www.wwpdb.org/validation/2017/XrayValidationReportHelp
with specific help available everywhere you see the (i) symbol.

The following versions of software and data (see references (i)) were used in the production of this report:

MolProbity	:	4.02b-467
Mogul	:	1.8.5 (274361), CSD as541be (2020)
Percentile statistics	:	20191225.v01 (using entries in the PDB archive December 25th 2019)
Ideal geometry (proteins)	:	Engh & Huber (2001)
Ideal geometry (DNA, RNA)	:	Parkinson et al. (1996)
Validation Pipeline (wwPDB-VP)	:	2.24

1 Overall quality at a glance (i)

The following experimental techniques were used to determine the structure:
NEUTRON DIFFRACTION

The reported resolution of this entry is 2.40 Å.

Percentile scores (ranging between 0-100) for global validation metrics of the entry are shown in the following graphic. The table shows the number of entries on which the scores are based.

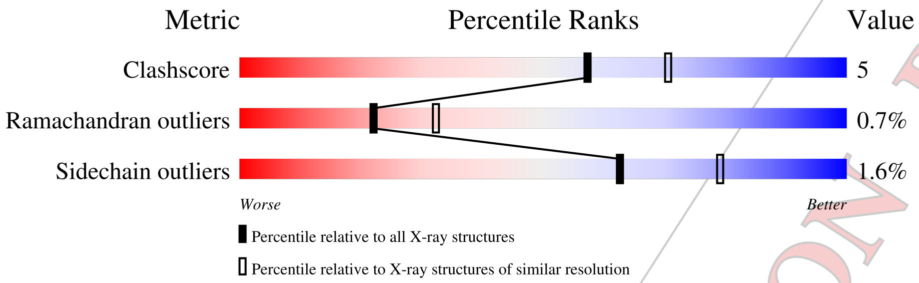

Metric	Whole archive (#Entries)	Similar resolution (#Entries, resolution range(Å))
Clashscore	141614	4398 (2.40-2.40)
Ramachandran outliers	138981	4318 (2.40-2.40)
Sidechain outliers	138945	4319 (2.40-2.40)

The table below summarises the geometric issues observed across the polymeric chains and their fit to the electron density. The red, orange, yellow and green segments of the lower bar indicate the fraction of residues that contain outliers for >=3, 2, 1 and 0 types of geometric quality criteria respectively. A grey segment represents the fraction of residues that are not modelled. The numeric value for each fraction is indicated below the corresponding segment, with a dot representing fractions <=5%

Note EDS was not executed.

Mol	Chain	Length	Quality of chain
1	A	223	93% 7%
1	B	223	96% •
2	C	2	100%
2	D	2	100%

The following table lists non-polymeric compounds, carbohydrate monomers and non-standard residues in protein, DNA, RNA chains that are outliers for geometric or electron-density-fit criteria:

PRELIMINARY VALIDATION REPORT

Mol	Type	Chain	Res	Chirality	Geometry	Clashes	Electron density
5	PEO	A	302[A]	-	-	X	-

PRELIMINARY VALIDATION REPORT

2 Entry composition (i)

There are 6 unique types of molecules in this entry. The entry contains 7793 atoms, of which 2643 are hydrogens and 1462 are deuteriums.

In the tables below, the ZeroOcc column contains the number of atoms modelled with zero occupancy, the AltConf column contains the number of residues with at least one atom in alternate conformation and the Trace column contains the number of residues modelled with at most 2 atoms.

- Molecule 1 is a protein called Lytic polysaccharide monooxygenase.

Mol	Chain	Residues	Atoms	ZeroOcc	AltConf	Trace
1	A	223	Total C D H N O S<br>3224 1018 308 1303 279 310 6	19	144	0
1	B	223	Total C D H N O S<br>3209 1006 313 1297 277 310 6	13	159	0

- Molecule 2 is an oligosaccharide called 2-acetamido-2-deoxy-beta-D-glucopyranose-(1-4)-2-acetamido-2-deoxy-beta-D-glucopyranose.

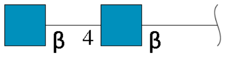

Mol	Chain	Residues	Atoms	ZeroOcc	AltConf	Trace
2	C	2	Total C D H N O<br>55 16 7 20 2 10	0	1	0
2	D	2	Total C D H N O<br>56 16 5 23 2 10	0	1	0

- Molecule 3 is COPPER (II) ION (three-letter code: CU) (formula: Cu).

Mol	Chain	Residues	Atoms	ZeroOcc	AltConf
3	A	1	Total Cu<br>1 1	0	0
3	B	1	Total Cu<br>1 1	0	0

- Molecule 4 is HYDROGEN PEROXIDE (three-letter code: PEO) (formula: H_2O_2).

PRELIMINARY VALIDATION REPORT

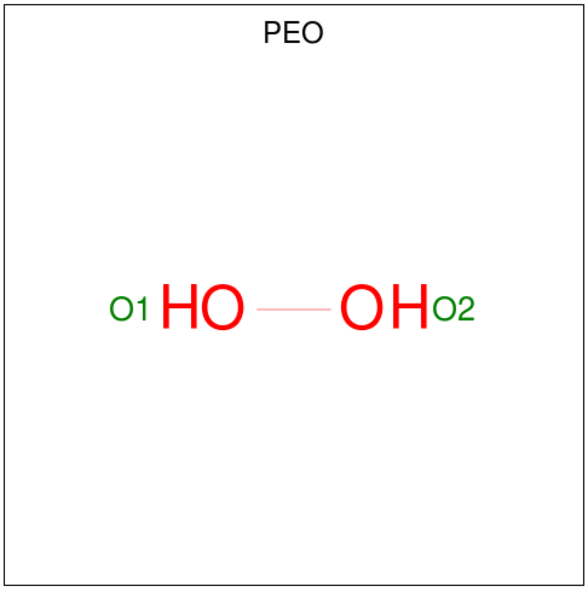

Mol	Chain	Residues	Atoms			ZeroOcc	AltConf
4	A	1	Total 3	D 1	O 2	0	1

- Molecule 5 is OXYGEN MOLECULE (three-letter code: PEO) (formula: H_2O_2).

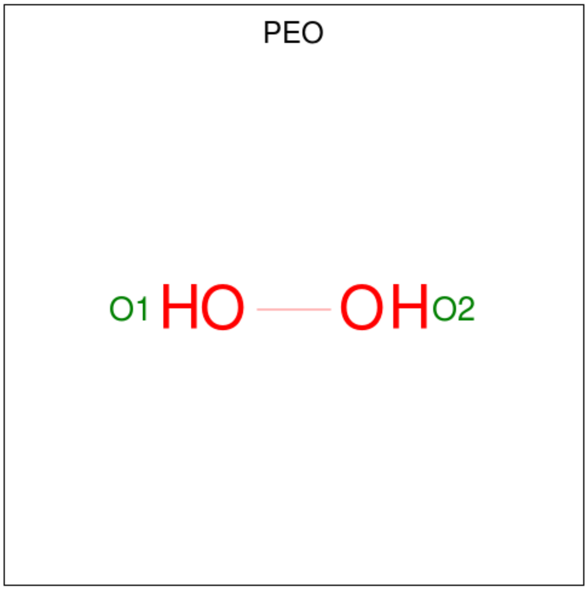

Mol	Chain	Residues	Atoms		ZeroOcc	AltConf
5	A	1	Total 2	O 2	0	1

- Molecule 6 is water.

PRELIMINARY VALIDATION REPORT

Mol	Chain	Residues	Atoms			ZeroOcc	AltConf
6	A	219	Total<br>660	D<br>440	O<br>220	0	1
6	B	194	Total<br>582	D<br>388	O<br>194	0	0

PRELIMINARY VALIDATION REPORT

3 Residue-property plots (i)

These plots are drawn for all protein, RNA, DNA and oligosaccharide chains in the entry. The first graphic for a chain summarises the proportions of the various outlier classes displayed in the second graphic. The second graphic shows the sequence view annotated by issues in geometry. Residues are color-coded according to the number of geometric quality criteria for which they contain at least one outlier: green = 0, yellow = 1, orange = 2 and red = 3 or more. Stretches of 2 or more consecutive residues without any outlier are shown as a green connector. Residues present in the sample, but not in the model, are shown in grey.

Note EDS was not executed.

- Molecule 1: Lytic polysaccharide monooxygenase

Chain A:

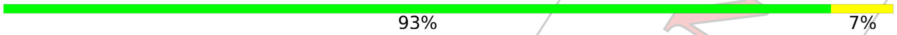

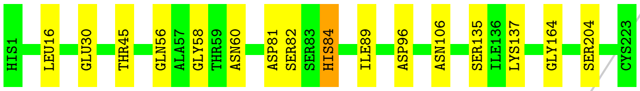

- Molecule 1: Lytic polysaccharide monooxygenase

Chain B:

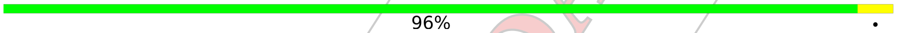

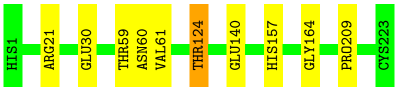

- Molecule 2: 2-acetamido-2-deoxy-beta-D-glucopyranose-(1-4)-2-acetamido-2-deoxy-beta-D-gluc opyranose

Chain C:

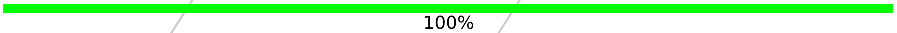

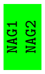

- Molecule 2: 2-acetamido-2-deoxy-beta-D-glucopyranose-(1-4)-2-acetamido-2-deoxy-beta-D-gluc opyranose

Chain D:

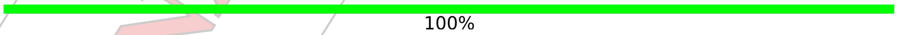

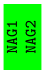

PRELIMINARY VALIDATION REPORT

4 Model quality (i)

4.1 Standard geometry (i)

Bond lengths and bond angles in the following residue types are not validated in this section: NAG, PEO, OXY, CU

The Z score for a bond length (or angle) is the number of standard deviations the observed value is removed from the expected value. A bond length (or angle) with $|Z| > 5$ is considered an outlier worth inspection. RMSZ is the root-mean-square of all Z scores of the bond lengths (or angles).

Mol	Chain	Bond lengths		Bond angles	
		RMSZ	#$\|Z\|$ >5	RMSZ	#$\|Z\|$ >5
1	A	0.31	0/2468	0.50	0/3372
1	B	0.35	0/2501	0.59	6/3415 (0.2%)
All	All	0.33	0/4969	0.55	6/6787 (0.1%)

Chiral center outliers are detected by calculating the chiral volume of a chiral center and verifying if the center is modelled as a planar moiety or with the opposite hand.A planarity outlier is detected by checking planarity of atoms in a peptide group, atoms in a mainchain group or atoms of a sidechain that are expected to be planar.

Mol	Chain	#Chirality outliers	#Planarity outliers
1	A	0	2
1	B	0	1
All	All	0	3

There are no bond length outliers.

All (6) bond angle outliers are listed below:

Mol	Chain	Res	Type	Atoms	Z	Observed(^{o})	Ideal(^{o})
1	B	21[A]	ARG	NE-CZ-NH2	-6.21	117.19	120.30
1	B	21[B]	ARG	NE-CZ-NH2	-6.21	117.19	120.30
1	B	21[A]	ARG	NE-CZ-NH1	5.99	123.29	120.30
1	B	21[B]	ARG	NE-CZ-NH1	5.99	123.29	120.30
1	B	59[A]	THR	O-C-N	-5.53	113.85	122.70
1	B	59[B]	THR	O-C-N	-5.53	113.85	122.70

There are no chirality outliers.

All (3) planarity outliers are listed below:

PRELIMINARY VALIDATION REPORT

Mol	Chain	Res	Type	Group
1	A	58[A]	GLY	Mainchain
1	A	58[B]	GLY	Mainchain
1	B	60[B]	ASN	Mainchain

4.2 Too-close contacts (i)

In the following table, the Non-H and H(model) columns list the number of non-hydrogen atoms and hydrogen atoms in the chain respectively. The H(added) column lists the number of hydrogen atoms added and optimized by MolProbity. The Clashes column lists the number of clashes within the asymmetric unit, whereas Symm-Clashes lists symmetry-related clashes.

Mol	Chain	Non-H	H(model)	H(added)	Clashes	Symm-Clashes
1	A	1921	1303	502	10	0
1	B	1912	1297	371	6	0
2	C	35	20	13	0	0
2	D	33	23	12	0	0
3	A	1	0	0	0	0
3	B	1	0	0	0	0
4	A	3	0	0	0	0
5	A	2	0	0	2	0
6	A	660	0	0	5	0
6	B	582	0	0	7	0
All	All	5150	2643	898	25	0

The all-atom clashscore is defined as the number of clashes found per 1000 atoms (including hydrogen atoms). The all-atom clashscore for this structure is 5.

All (25) close contacts within the same asymmetric unit are listed below, sorted by their clash magnitude.

Atom-1	Atom-2	Interatomic distance (Å)	Clash overlap (Å)
1:A:204:SER:O	6:A:579:HOH:O	2.02	0.76
1:A:16:LEU:HD21	6:A:490:HOH:O	1.82	0.74
5:A:302[A]:PEO:O2	1:B:30:GLU:OE1	2.08	0.71
1:A:30:GLU:OE1	6:A:536:HOH:O	2.12	0.67
1:A:84:HIS:CE1	5:A:302[A]:PEO:O1	2.62	0.51
1:A:81:ASP:OD1	1:A:82:SER:N	2.48	0.46
1:A:60:ASN:ND2	1:A:135:SER:OG	2.49	0.45
1:B:61:VAL:O	1:B:61:VAL:HG23	2.15	0.41

There are no symmetry-related clashes.

PRELIMINARY VALIDATION REPORT

4.3 Torsion angles (i)

4.3.1 Protein backbone (i)

In the following table, the Percentiles column shows the percent Ramachandran outliers of the chain as a percentile score with respect to all X-ray entries followed by that with respect to entries of similar resolution.

The Analysed column shows the number of residues for which the backbone conformation was analysed, and the total number of residues.

Mol	Chain	Analysed	Favoured	Allowed	Outliers	Percentiles	
1	A	325/223 (146%)	304 (94%)	19 (6%)	2 (1%)	25	36
1	B	330/223 (148%)	305 (92%)	23 (7%)	2 (1%)	25	36
All	All	655/446 (147%)	609 (93%)	42 (6%)	4 (1%)	22	36

All (4) Ramachandran outliers are listed below:

Mol	Chain	Res	Type
1	B	164[B]	GLY
1	A	164[A]	GLY
1	A	164[B]	GLY
1	B	209	PRO

4.3.2 Protein sidechains (i)

In the following table, the Percentiles column shows the percent sidechain outliers of the chain as a percentile score with respect to all X-ray entries followed by that with respect to entries of similar resolution.

The Analysed column shows the number of residues for which the sidechain conformation was analysed, and the total number of residues.

Mol	Chain	Analysed	Rotameric	Outliers	Percentiles	
1	A	242/178 (136%)	236 (98%)	6 (2%)	47	67
1	B	242/178 (136%)	239 (99%)	3 (1%)	71	85
All	All	484/356 (136%)	475 (98%)	9 (2%)	62	75

All (9) residues with a non-rotameric sidechain are listed below:

Mol	Chain	Res	Type
1	A	56[A]	GLN
1	A	56[B]	GLN

Continued on next page...

PRELIMINARY VALIDATION REPORT

Continued from previous page...

Mol	Chain	Res	Type
1	A	56[C]	GLN
1	A	56[D]	GLN
1	A	84	HIS
1	A	89[A]	ILE
1	B	124[A]	THR
1	B	124[B]	THR
1	B	140[B]	GLU

Sometimes sidechains can be flipped to improve hydrogen bonding and reduce clashes. There are no such sidechains identified.

4.3.3 RNA (i)

There are no RNA molecules in this entry.

4.4 Non-standard residues in protein, DNA, RNA chains (i)

There are no non-standard protein/DNA/RNA residues in this entry.

4.5 Carbohydrates (i)

5 monosaccharides are modelled in this entry.

In the following table, the Counts columns list the number of bonds (or angles) for which Mogul statistics could be retrieved, the number of bonds (or angles) that are observed in the model and the number of bonds (or angles) that are defined in the Chemical Component Dictionary. The Link column lists molecule types, if any, to which the group is linked. The Z score for a bond length (or angle) is the number of standard deviations the observed value is removed from the expected value. A bond length (or angle) with $|Z| > 2$ is considered an outlier worth inspection. RMSZ is the root-mean-square of all Z scores of the bond lengths (or angles).

Mol	Type	Chain	Res	Link	Bond lengths			Bond angles		
					Counts	RMSZ	$\#\|Z\| > 2$	Counts	RMSZ	$\#\|Z\| > 2$
2	NAG	C	2	2	14,14,15	0.21	0	17,19,21	0.40	0
2	NAG	D	1	2,1	14,14,15	0.27	0	17,19,21	0.43	0

In the following table, the Chirals column lists the number of chiral outliers, the number of chiral centers analysed, the number of these observed in the model and the number defined in the Chemical Component Dictionary. Similar counts are reported in the Torsion and Rings columns. '-' means no outliers of that kind were identified.

PRELIMINARY VALIDATION REPORT

Mol	Type	Chain	Res	Link	Chirals	Torsions	Rings
2	NAG	C	2	2	-	0/6/23/26	0/1/1/1
2	NAG	D	1	2,1	-	1/6/23/26	0/1/1/1

There are no bond length outliers.

There are no bond angle outliers.

There are no chirality outliers.

All (1) torsion outliers are listed below:

Mol	Chain	Res	Type	Atoms
2	D	1	NAG	O5-C5-C6-O6

There are no ring outliers.

No monomer is involved in short contacts.

The following is a two-dimensional graphical depiction of Mogul quality analysis of bond lengths, bond angles, torsion angles, and ring geometry for oligosaccharide.

PRELIMINARY VALIDATION REPORT

Oligosaccharide Chain C

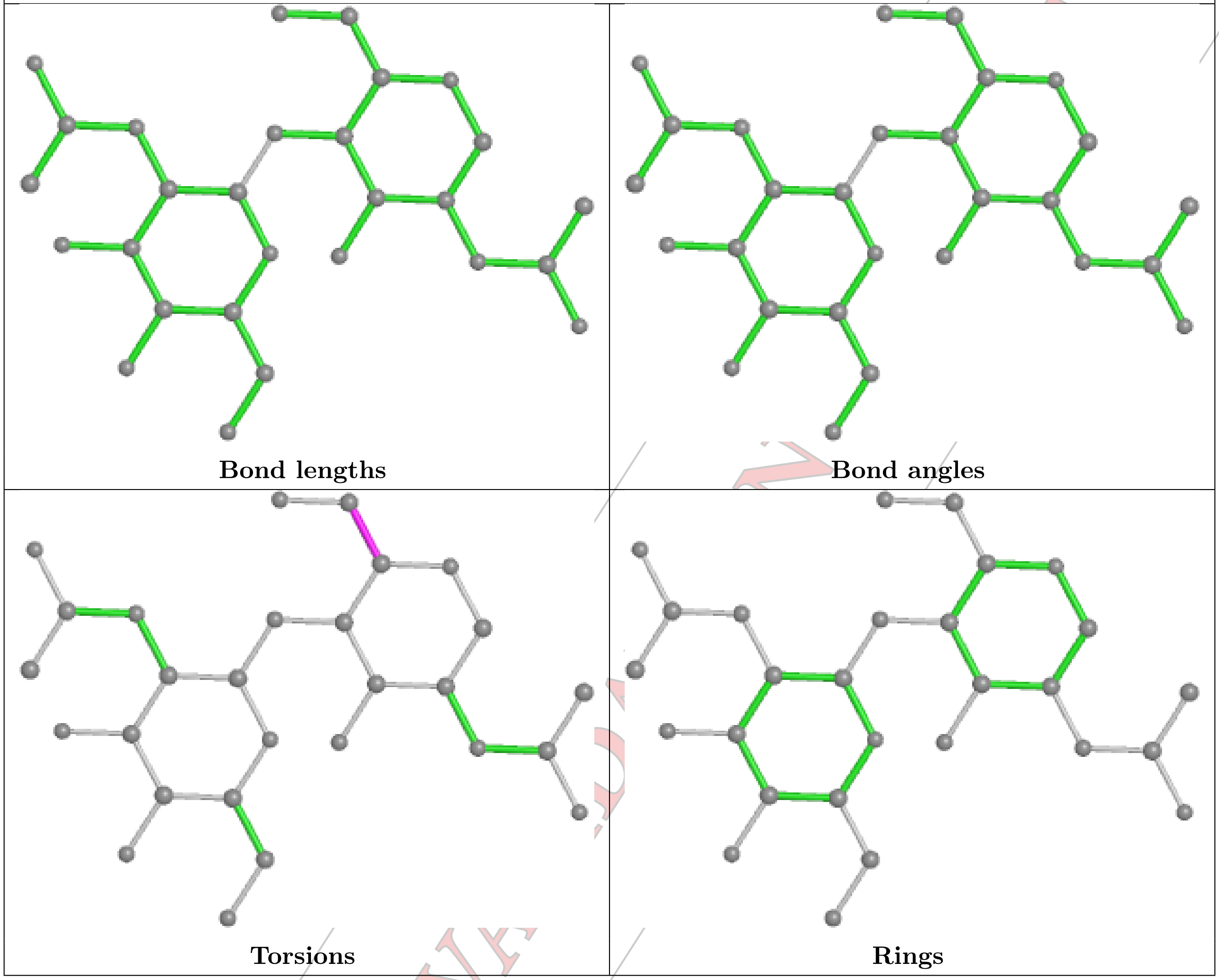

PRELIMINARY

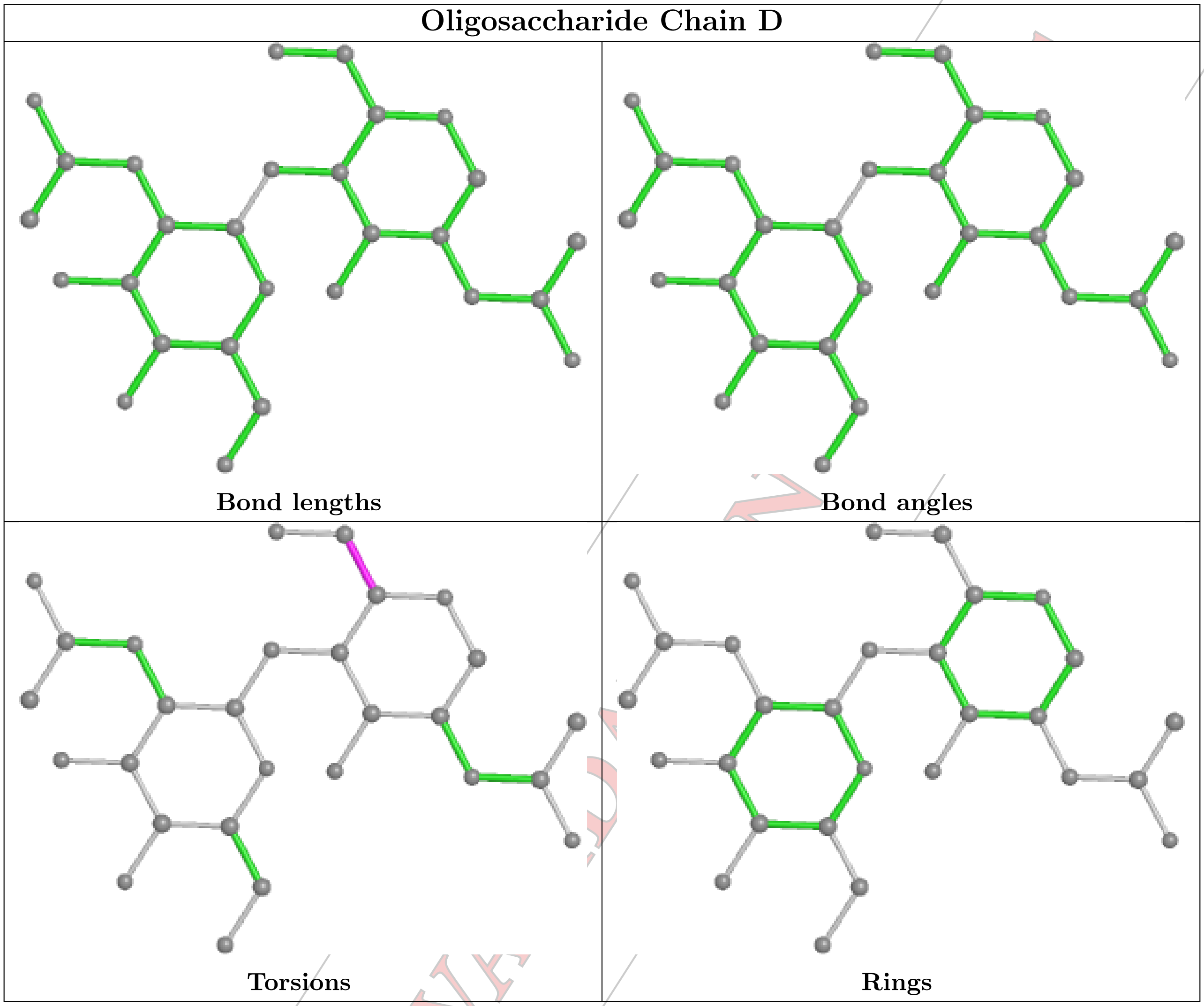

4.6 Ligand geometry (i)

Of 4 ligands modelled in this entry, 2 are monoatomic - leaving 2 for Mogul analysis.

In the following table, the Counts columns list the number of bonds (or angles) for which Mogul statistics could be retrieved, the number of bonds (or angles) that are observed in the model and the number of bonds (or angles) that are defined in the Chemical Component Dictionary. The Link column lists molecule types, if any, to which the group is linked. The Z score for a bond length (or angle) is the number of standard deviations the observed value is removed from the expected value. A bond length (or angle) with $|Z| > 2$ is considered an outlier worth inspection. RMSZ is the root-mean-square of all Z scores of the bond lengths (or angles).

Mol	Type	Chain	Res	Link	Bond lengths			Bond angles		
					Counts	RMSZ	$\#\|Z\| > 2$	Counts	RMSZ	$\#\|Z\| > 2$
5	PEO	A	302[A]	3	1,1,1	0.40	0	-		
5	OXY	A	302[B]	3	1,1,1	0.26	0	-		

There are no bond length outliers.

There are no bond angle outliers.

There are no chirality outliers.

There are no torsion outliers.

There are no ring outliers.

1 monomer is involved in 2 short contacts:

Mol	Chain	Res	Type	Clashes	Symm-Clashes
5	A	302[A]	PEO	2	0

4.7 Other polymers (i)

There are no such residues in this entry.

4.8 Polymer linkage issues (i)

There are no chain breaks in this entry.

PRELIMINARY VALIDATION REPORT

WORLDWIDE PDB PROTEIN DATA BANK

Preliminary Full wwPDB X-ray Structure Validation Report (i)

Dec 8, 2021 – 02:27 PM EST

Deposition ID : D_1000261514

This is a Preliminary Full wwPDB X-ray Structure Validation Report.

This report is produced by the wwPDB Deposition System during initial deposition but before annotation of the structure.

We welcome your comments at *validation@mail.wwpdb.org*
A user guide is available at
https://www.wwpdb.org/validation/2017/XrayValidationReportHelp
with specific help available everywhere you see the (i) symbol.

The following versions of software and data (see references (i)) were used in the production of this report:

MolProbity	:	4.02b-467
Mogul	:	1.8.5 (274361), CSD as541be (2020)
Xtriage (Phenix)	:	1.13
EDS	:	FAILED
Percentile statistics	:	20191225.v01 (using entries in the PDB archive December 25th 2019)
Ideal geometry (proteins)	:	Engh & Huber (2001)
Ideal geometry (DNA, RNA)	:	Parkinson et al. (1996)
Validation Pipeline (wwPDB-VP)	:	2.24

PRELIMINARY VALIDATION REPORT

1 Overall quality at a glance (i)

The following experimental techniques were used to determine the structure:
X-RAY DIFFRACTION, NEUTRON DIFFRACTION

The reported resolution of this entry is 1.90 Å.

Percentile scores (ranging between 0-100) for global validation metrics of the entry are shown in the following graphic. The table shows the number of entries on which the scores are based.

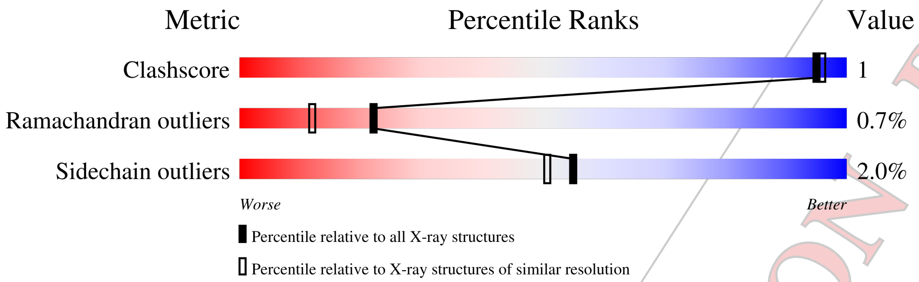

Metric	Whole archive (#Entries)	Similar resolution (#Entries, resolution range(Å))
Clashscore	141614	6847 (1.90-1.90)
Ramachandran outliers	138981	6760 (1.90-1.90)
Sidechain outliers	138945	6760 (1.90-1.90)

The table below summarises the geometric issues observed across the polymeric chains and their fit to the electron density. The red, orange, yellow and green segments of the lower bar indicate the fraction of residues that contain outliers for >=3, 2, 1 and 0 types of geometric quality criteria respectively. A grey segment represents the fraction of residues that are not modelled. The numeric value for each fraction is indicated below the corresponding segment, with a dot representing fractions <=5%

Note EDS failed to run properly.

Mol	Chain	Length	Quality of chain
1	A	223	95% 5%
1	B	223	98% •
2	C	3	67% 33%
2	D	3	67% 33%

PRELIMINARY VALIDATION REPORT

2 Entry composition (i)

There are 4 unique types of molecules in this entry. The entry contains 8385 atoms, of which 3062 are hydrogens and 1472 are deuteriums.

In the tables below, the ZeroOcc column contains the number of atoms modelled with zero occupancy, the AltConf column contains the number of residues with at least one atom in alternate conformation and the Trace column contains the number of residues modelled with at most 2 atoms.

- Molecule 1 is a protein.

Mol	Chain	Residues	Atoms							ZeroOcc	AltConf	Trace
			Total	C	D	H	N	O	S			
1	A	223	3502	1058	335	1489	279	332	9	0	197	0
1	B	223	3559	1077	339	1516	282	335	10	0	197	0

- Molecule 2 is an oligosaccharide called alpha-D-mannopyranose-(1-4)-2-acetamido-2-deoxy-beta-D-glucopyranose-(1-4)-2-acetamido-2-deoxy-beta-D-glucopyranose.

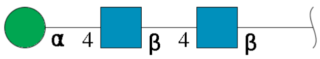

Mol	Chain	Residues	Atoms						ZeroOcc	AltConf	Trace
			Total	C	D	H	N	O			
2	C	3	77	22	10	28	2	15	0	2	0
2	D	3	78	22	10	29	2	15	0	2	0

- Molecule 3 is COPPER (II) ION (three-letter code: CU) (formula: Cu).

Mol	Chain	Residues	Atoms		ZeroOcc	AltConf
			Total	Cu		
3	A	1	1	1	0	0
3	B	1	1	1	0	0

- Molecule 4 is water.

Mol	Chain	Residues	Atoms			ZeroOcc	AltConf
			Total	D	O		
4	A	183	552	368	184	0	1

Continued on next page...

PRELIMINARY VALIDATION REPORT

Continued from previous page...

Mol	Chain	Residues	Atoms			ZeroOcc	AltConf
			Total	D	O		
4	B	205	615	410	205	0	0

PRELIMINARY VALIDATION REPORT

3 Residue-property plots (i)

These plots are drawn for all protein, RNA, DNA and oligosaccharide chains in the entry. The first graphic for a chain summarises the proportions of the various outlier classes displayed in the second graphic. The second graphic shows the sequence view annotated by issues in geometry. Residues are color-coded according to the number of geometric quality criteria for which they contain at least one outlier: green = 0, yellow = 1, orange = 2 and red = 3 or more. Stretches of 2 or more consecutive residues without any outlier are shown as a green connector. Residues present in the sample, but not in the model, are shown in grey.

Note EDS failed to run properly.

- Molecule 1:

Chain A:

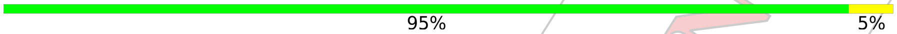

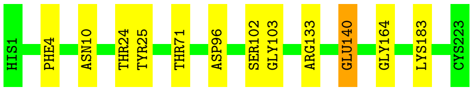

- Molecule 1:

Chain B:

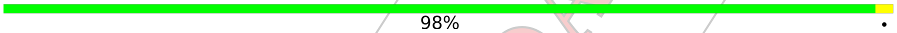

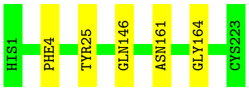

- Molecule 2: alpha-D-mannopyranose-(1-4)-2-acetamido-2-deoxy-beta-D-glucopyranose-(1-4)-2-acetamido-2-deoxy-beta-D-glucopyranose

Chain C:

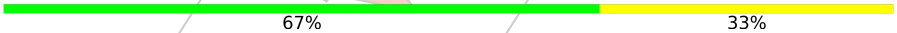

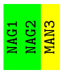

- Molecule 2: alpha-D-mannopyranose-(1-4)-2-acetamido-2-deoxy-beta-D-glucopyranose-(1-4)-2-acetamido-2-deoxy-beta-D-glucopyranose

Chain D:

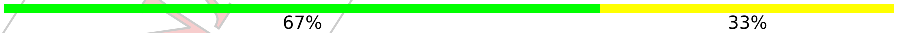

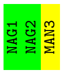

PRELIMINARY VALIDATION REPORT

4 Data and refinement statistics (i)

EDS failed to run properly - this section is therefore incomplete.

Property	Value	Source
Space group	P 1 21 1	Depositor
Cell constants<br>a, b, c, α, β, γ	68.30Å 42.27Å 70.41Å<br>90.00° 98.47° 90.00°	Depositor
Resolution (Å)	12.66 – 1.90	Depositor
% Data completeness<br>(in resolution range)	98.0 (12.66-1.90)	Depositor
R_{merge}	(Not available)	Depositor
R_{sym}	(Not available)	Depositor
$< I/\sigma(I) >$ [1]	4.02 (at 1.90Å)	Xtriage
Refinement program	PHENIX 1.14_3260	Depositor
R, R_{free}	0.128 , 0.182	Depositor
Wilson B-factor (Å^2)	16.5	Xtriage
Anisotropy	0.598	Xtriage
L-test for twinning[2]	$< \|L\| >$ = 0.49, $< L^2 >$ = 0.33	Xtriage
Estimated twinning fraction	0.017 for l,-k,h	Xtriage
Total number of atoms	8385	wwPDB-VP
Average B, all atoms (Å^2)	23.0	wwPDB-VP

Xtriage's analysis on translational NCS is as follows: *The largest off-origin peak in the Patterson function is 9.52% of the height of the origin peak. No significant pseudotranslation is detected.*

[1] Intensities estimated from amplitudes.

[2] Theoretical values of $< |L| >$, $< L^2 >$ for acentric reflections are 0.5, 0.333 respectively for untwinned datasets, and 0.375, 0.2 for perfectly twinned datasets.

PRELIMINARY VALIDATION REPORT

5 Model quality ⓘ

5.1 Standard geometry ⓘ

Bond lengths and bond angles in the following residue types are not validated in this section: CU, NAG, MAN

The Z score for a bond length (or angle) is the number of standard deviations the observed value is removed from the expected value. A bond length (or angle) with $|Z| > 5$ is considered an outlier worth inspection. RMSZ is the root-mean-square of all Z scores of the bond lengths (or angles).

Mol	Chain	Bond lengths		Bond angles	
		RMSZ	#$\|Z\|$ >5	RMSZ	#$\|Z\|$ >5
1	A	0.67	0/2807	0.72	2/3832 (0.1%)
1	B	0.67	0/2778	0.79	0/3789
All	All	0.67	0/5585	0.75	2/7621 (0.0%)

Chiral center outliers are detected by calculating the chiral volume of a chiral center and verifying if the center is modelled as a planar moiety or with the opposite hand.A planarity outlier is detected by checking planarity of atoms in a peptide group, atoms in a mainchain group or atoms of a sidechain that are expected to be planar.

Mol	Chain	#Chirality outliers	#Planarity outliers
1	A	0	1

There are no bond length outliers.

All (2) bond angle outliers are listed below:

Mol	Chain	Res	Type	Atoms	Z	Observed(^{o})	Ideal(^{o})
1	A	133[A]	ARG	NE-CZ-NH2	-5.69	117.46	120.30
1	A	133[B]	ARG	NE-CZ-NH2	-5.69	117.46	120.30

There are no chirality outliers.

All (1) planarity outliers are listed below:

Mol	Chain	Res	Type	Group
1	A	102[B]	SER	Peptide

5.2 Too-close contacts ⓘ

In the following table, the Non-H and H(model) columns list the number of non-hydrogen atoms and hydrogen atoms in the chain respectively. The H(added) column lists the number of hydrogen

PRELIMINARY VALIDATION REPORT

atoms added and optimized by MolProbity. The Clashes column lists the number of clashes within the asymmetric unit, whereas Symm-Clashes lists symmetry-related clashes.

Mol	Chain	Non-H	H(model)	H(added)	Clashes	Symm-Clashes
1	A	2013	1489	292	4	0
1	B	2043	1516	382	3	0
2	C	49	28	10	0	0
2	D	49	29	10	0	0
3	A	1	0	0	0	0
3	B	1	0	0	0	0
4	A	552	0	0	3	0
4	B	615	0	0	4	0
All	All	5323	3062	694	8	0

The all-atom clashscore is defined as the number of clashes found per 1000 atoms (including hydrogen atoms). The all-atom clashscore for this structure is 1.

All (8) close contacts within the same asymmetric unit are listed below, sorted by their clash magnitude.

Atom-1	Atom-2	Interatomic distance (Å)	Clash overlap (Å)
1:B:25[B]:TYR:CZ	4:B:420:HOH:O	1.71	1.34
1:B:25[B]:TYR:CE1	4:B:420:HOH:O	1.76	1.10
1:A:71:THR:O	4:A:517:HOH:O	2.19	0.60
1:A:140[A]:GLU:OE1	4:A:575:HOH:O	2.23	0.51
1:A:96:ASP:HB3	4:A:581:HOH:O	2.10	0.45
1:B:161[B]:ASN:ND2	4:B:531:HOH:O	2.52	0.41

There are no symmetry-related clashes.

5.3 Torsion angles (i)

5.3.1 Protein backbone (i)

In the following table, the Percentiles column shows the percent Ramachandran outliers of the chain as a percentile score with respect to all X-ray entries followed by that with respect to entries of similar resolution.

The Analysed column shows the number of residues for which the backbone conformation was analysed, and the total number of residues.

Mol	Chain	Analysed	Favoured	Allowed	Outliers	Percentiles
1	A	369/223 (166%)	356 (96%)	10 (3%)	3 (1%)	19 9

Continued on next page...

PRELIMINARY VALIDATION REPORT

Continued from previous page...

Mol	Chain	Analysed	Favoured	Allowed	Outliers	Percentiles	
1	B	365/223 (164%)	354 (97%)	9 (2%)	2 (0%)	29	18
All	All	734/446 (165%)	710 (97%)	19 (3%)	5 (1%)	22	12

All (5) Ramachandran outliers are listed below:

Mol	Chain	Res	Type
1	A	103[A]	GLY
1	A	103[B]	GLY
1	A	164[B]	GLY
1	B	164[A]	GLY
1	B	164[B]	GLY

5.3.2 Protein sidechains (i)

In the following table, the Percentiles column shows the percent sidechain outliers of the chain as a percentile score with respect to all X-ray entries followed by that with respect to entries of similar resolution.

The Analysed column shows the number of residues for which the sidechain conformation was analysed, and the total number of residues.

Mol	Chain	Analysed	Rotameric	Outliers	Percentiles	
1	A	294/178 (165%)	285 (97%)	9 (3%)	40	32
1	B	298/178 (167%)	294 (99%)	4 (1%)	69	68
All	All	592/356 (166%)	579 (98%)	13 (2%)	55	47

All (13) residues with a non-rotameric sidechain are listed below:

Mol	Chain	Res	Type
1	A	4[A]	PHE
1	A	4[B]	PHE
1	A	10[A]	ASN
1	A	10[B]	ASN
1	A	24	THR
1	A	140[A]	GLU
1	A	140[B]	GLU
1	A	183[A]	LYS
1	A	183[B]	LYS
1	B	4[A]	PHE
1	B	4[B]	PHE

Continued on next page...

PRELIMINARY VALIDATION REPORT

Continued from previous page...

Mol	Chain	Res	Type
1	B	146[A]	GLN
1	B	146[B]	GLN

Sometimes sidechains can be flipped to improve hydrogen bonding and reduce clashes. There are no such sidechains identified.

5.3.3 RNA (i)

There are no RNA molecules in this entry.

5.4 Non-standard residues in protein, DNA, RNA chains (i)

There are no non-standard protein/DNA/RNA residues in this entry.

5.5 Carbohydrates (i)

10 monosaccharides are modelled in this entry.

In the following table, the Counts columns list the number of bonds (or angles) for which Mogul statistics could be retrieved, the number of bonds (or angles) that are observed in the model and the number of bonds (or angles) that are defined in the Chemical Component Dictionary. The Link column lists molecule types, if any, to which the group is linked. The Z score for a bond length (or angle) is the number of standard deviations the observed value is removed from the expected value. A bond length (or angle) with $|Z| > 2$ is considered an outlier worth inspection. RMSZ is the root-mean-square of all Z scores of the bond lengths (or angles).

Mol	Type	Chain	Res	Link	Bond lengths			Bond angles		
					Counts	RMSZ	$\#\|Z\| > 2$	Counts	RMSZ	$\#\|Z\| > 2$
2	MAN	C	3	2	11,11,12	1.74	3 (27%)	15,15,17	1.30	2 (13%)
2	MAN	D	3	2	11,11,12	0.84	0	15,15,17	3.68	6 (40%)

In the following table, the Chirals column lists the number of chiral outliers, the number of chiral centers analysed, the number of these observed in the model and the number defined in the Chemical Component Dictionary. Similar counts are reported in the Torsion and Rings columns. '-' means no outliers of that kind were identified.

Mol	Type	Chain	Res	Link	Chirals	Torsions	Rings
2	MAN	C	3	2	-	2/2/19/22	0/1/1/1
2	MAN	D	3	2	-	2/2/19/22	0/1/1/1

PRELIMINARY VALIDATION REPORT

All (3) bond length outliers are listed below:

Mol	Chain	Res	Type	Atoms	Z	Observed(Å)	Ideal(Å)
2	C	3	MAN	C2-C3	2.93	1.56	1.52
2	C	3	MAN	C4-C3	2.52	1.58	1.52
2	C	3	MAN	C4-C5	2.36	1.58	1.53

All (8) bond angle outliers are listed below:

Mol	Chain	Res	Type	Atoms	Z	Observed(°)	Ideal(°)
2	D	3	MAN	O2-C2-C1	-7.56	93.69	109.15
2	D	3	MAN	C1-O5-C5	7.55	122.42	112.19
2	D	3	MAN	O5-C1-C2	6.60	120.97	110.77
2	D	3	MAN	C1-C2-C3	4.98	115.79	109.67
2	C	3	MAN	C1-O5-C5	3.44	116.86	112.19
2	D	3	MAN	O5-C5-C6	3.25	112.30	107.20
2	C	3	MAN	O5-C1-C2	2.37	114.42	110.77
2	D	3	MAN	O2-C2-C3	2.05	114.24	110.14

There are no chirality outliers.

All (4) torsion outliers are listed below:

Mol	Chain	Res	Type	Atoms
2	D	3	MAN	O5-C5-C6-O6
2	C	3	MAN	O5-C5-C6-O6
2	D	3	MAN	C4-C5-C6-O6
2	C	3	MAN	C4-C5-C6-O6

There are no ring outliers.

No monomer is involved in short contacts.

The following is a two-dimensional graphical depiction of Mogul quality analysis of bond lengths, bond angles, torsion angles, and ring geometry for oligosaccharide.

PRELIMINARY VALIDATION REPORT

Oligosaccharide Chain C

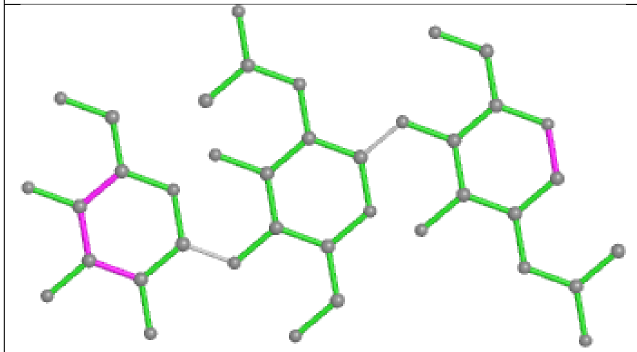

Bond lengths

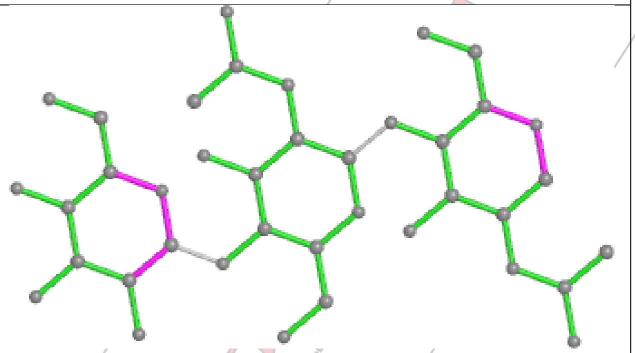

Bond angles

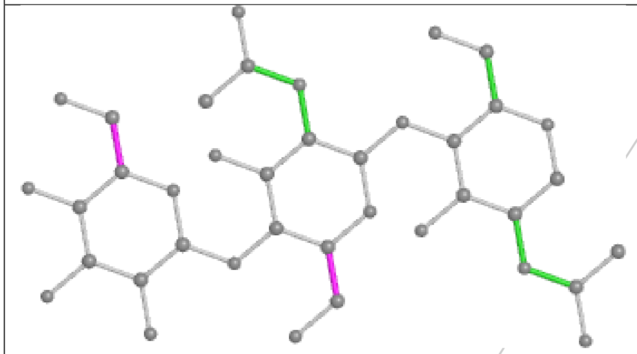

Torsions

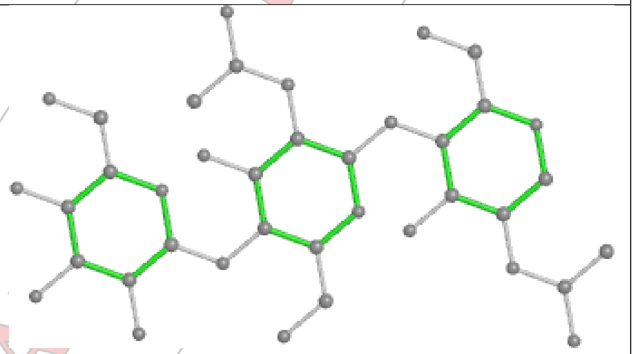

Rings

Oligosaccharide Chain D

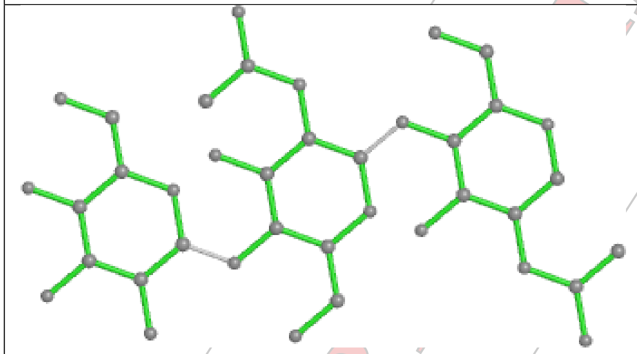

Bond lengths

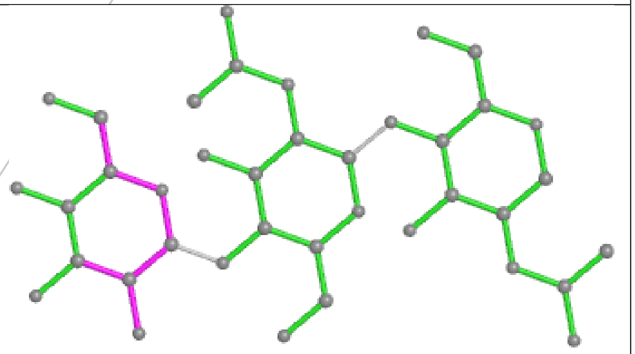

Bond angles

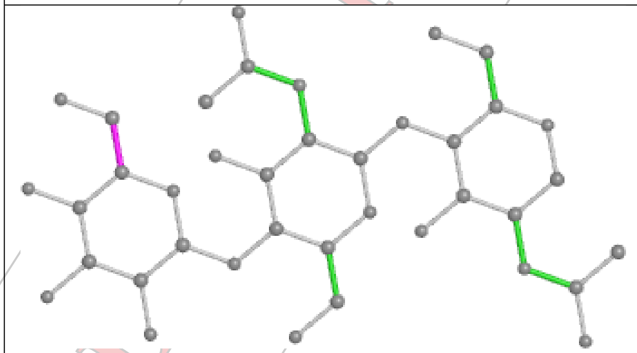

Torsions

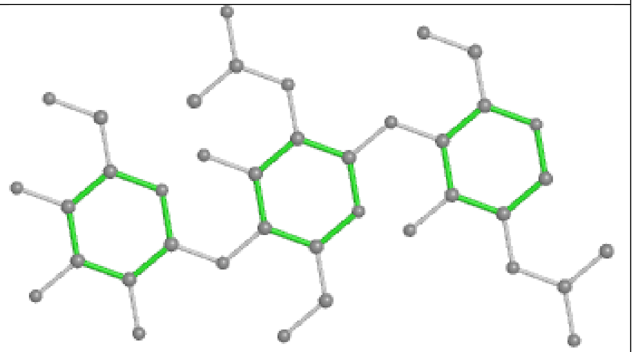

Rings

5.6 Ligand geometry (i)

Of 2 ligands modelled in this entry, 2 are monoatomic - leaving 0 for Mogul analysis.

There are no bond length outliers.

There are no bond angle outliers.

There are no chirality outliers.

There are no torsion outliers.

There are no ring outliers.

No monomer is involved in short contacts.

5.7 Other polymers (i)

There are no such residues in this entry.

5.8 Polymer linkage issues (i)

There are no chain breaks in this entry.

PRELIMINARY VALIDATION REPORT

6 Fit of model and data (i)

6.1 Protein, DNA and RNA chains (i)

EDS failed to run properly - this section is therefore empty.

6.2 Non-standard residues in protein, DNA, RNA chains (i)

EDS failed to run properly - this section is therefore empty.

6.3 Carbohydrates (i)

EDS failed to run properly - this section is therefore empty.

6.4 Ligands (i)

EDS failed to run properly - this section is therefore empty.

6.5 Other polymers (i)

EDS failed to run properly - this section is therefore empty.

PRELIMINARY VALIDATION REPORT

Preliminary Full wwPDB X-ray Structure Validation Report ⓘ

Dec 2, 2021 – 04:10 PM EST

Deposition ID : D_1000261512

This is a Preliminary Full wwPDB X-ray Structure Validation Report.

This report is produced by the wwPDB Deposition System during initial deposition but before annotation of the structure.

We welcome your comments at *validation@mail.wwpdb.org*
A user guide is available at
https://www.wwpdb.org/validation/2017/XrayValidationReportHelp
with specific help available everywhere you see the ⓘ symbol.

PRELIMINARY VALIDATION REPORT

The following versions of software and data (see references ⓘ) were used in the production of this report:

MolProbity	:	4.02b-467
Mogul	:	1.8.5 (274361), CSD as541be (2020)
Xtriage (Phenix)	:	1.13
EDS	:	2.24
Percentile statistics	:	20191225.v01 (using entries in the PDB archive December 25th 2019)
Refmac	:	5.8.0158
CCP4	:	7.0.044 (Gargrove)
Ideal geometry (proteins)	:	Engh & Huber (2001)
Ideal geometry (DNA, RNA)	:	Parkinson et al. (1996)
Validation Pipeline (wwPDB-VP)	:	2.24

1 Overall quality at a glance (i)

The following experimental techniques were used to determine the structure:
X-RAY DIFFRACTION

The reported resolution of this entry is 1.50 Å.

Percentile scores (ranging between 0-100) for global validation metrics of the entry are shown in the following graphic. The table shows the number of entries on which the scores are based.

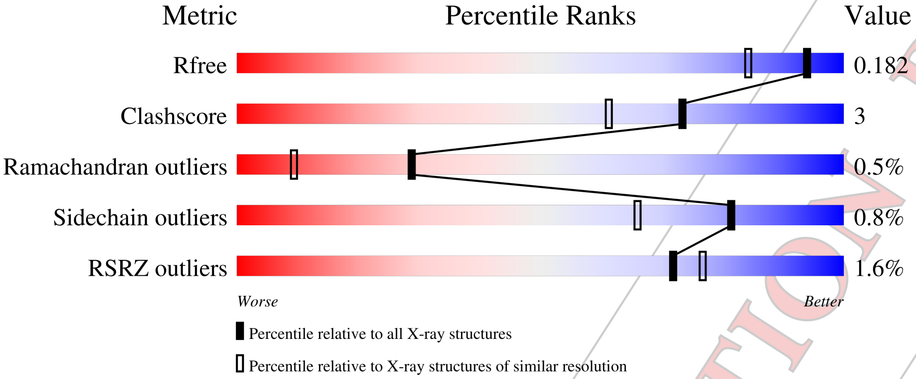

Metric	Whole archive (#Entries)	Similar resolution (#Entries, resolution range(Å))
R_{free}	130704	2936 (1.50-1.50)
Clashscore	141614	3144 (1.50-1.50)
Ramachandran outliers	138981	3066 (1.50-1.50)
Sidechain outliers	138945	3064 (1.50-1.50)
RSRZ outliers	127900	2884 (1.50-1.50)

The table below summarises the geometric issues observed across the polymeric chains and their fit to the electron density. The red, orange, yellow and green segments of the lower bar indicate the fraction of residues that contain outliers for >=3, 2, 1 and 0 types of geometric quality criteria respectively. A grey segment represents the fraction of residues that are not modelled. The numeric value for each fraction is indicated below the corresponding segment, with a dot representing fractions <=5% The upper red bar (where present) indicates the fraction of residues that have poor fit to the electron density. The numeric value is given above the bar.

Mol	Chain	Length	Quality of chain
1	A	223	3% 92% 8%
1	B	223	91% 8%
2	C	2	50% 50%
3	D	3	33% 67%

PRELIMINARY VALIDATION REPORT

2 Entry composition (i)

There are 5 unique types of molecules in this entry. The entry contains 4327 atoms, of which 0 are hydrogens and 0 are deuteriums.

In the tables below, the ZeroOcc column contains the number of atoms modelled with zero occupancy, the AltConf column contains the number of residues with at least one atom in alternate conformation and the Trace column contains the number of residues modelled with at most 2 atoms.

- Molecule 1 is a protein.

Mol	Chain	Residues	Atoms					ZeroOcc	AltConf	Trace
			Total	C	N	O	S			
1	A	223	1729	1090	286	343	10	0	17	0
1	B	223	1752	1104	288	350	10	0	22	0

- Molecule 2 is an oligosaccharide called 2-acetamido-2-deoxy-beta-D-glucopyranose-(1-4)-2-acetamido-2-deoxy-beta-D-glucopyranose.

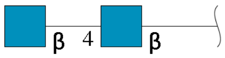

Mol	Chain	Residues	Atoms				ZeroOcc	AltConf	Trace
			Total	C	N	O			
2	C	2	28	16	2	10	0	0	0

- Molecule 3 is an oligosaccharide called alpha-D-mannopyranose-(1-4)-2-acetamido-2-deoxy-beta-D-glucopyranose-(1-4)-2-acetamido-2-deoxy-beta-D-glucopyranose.

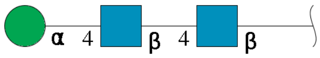

Mol	Chain	Residues	Atoms				ZeroOcc	AltConf	Trace
			Total	C	N	O			
3	D	3	39	22	2	15	0	0	0

- Molecule 4 is COPPER (II) ION (three-letter code: CU) (formula: Cu).

PRELIMINARY VALIDATION REPORT

Mol	Chain	Residues	Atoms	ZeroOcc	AltConf
4	A	1	Total Cu<br>1 1	0	0
4	B	1	Total Cu<br>1 1	0	0

- Molecule 5 is water.

Mol	Chain	Residues	Atoms	ZeroOcc	AltConf
5	A	366	Total O<br>366 366	0	0
5	B	411	Total O<br>411 411	0	0

PRELIMINARY VALIDATION REPORT

3 Residue-property plots (i)

These plots are drawn for all protein, RNA, DNA and oligosaccharide chains in the entry. The first graphic for a chain summarises the proportions of the various outlier classes displayed in the second graphic. The second graphic shows the sequence view annotated by issues in geometry and electron density. Residues are color-coded according to the number of geometric quality criteria for which they contain at least one outlier: green = 0, yellow = 1, orange = 2 and red = 3 or more. A red dot above a residue indicates a poor fit to the electron density (RSRZ > 2). Stretches of 2 or more consecutive residues without any outlier are shown as a green connector. Residues present in the sample, but not in the model, are shown in grey.

- Molecule 1:

Chain A:

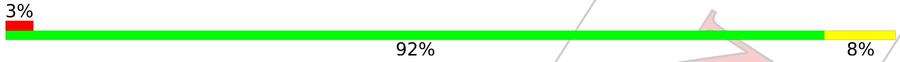

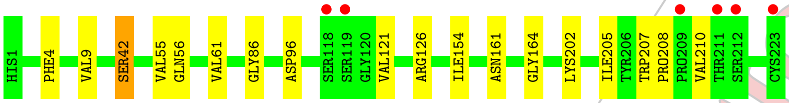

- Molecule 1:

Chain B:

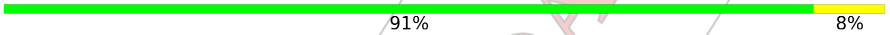

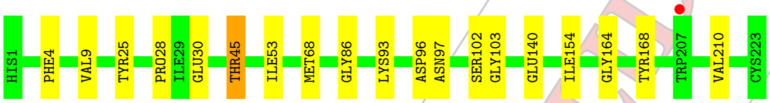

- Molecule 2: 2-acetamido-2-deoxy-beta-D-glucopyranose-(1-4)-2-acetamido-2-deoxy-beta-D-glucopyranose

Chain C:

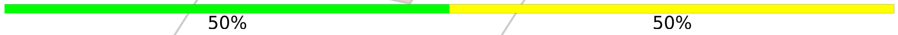

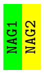

- Molecule 3: alpha-D-mannopyranose-(1-4)-2-acetamido-2-deoxy-beta-D-glucopyranose-(1-4)-2-acetamido-2-deoxy-beta-D-glucopyranose

Chain D:

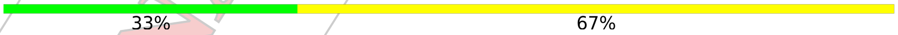

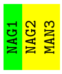

PRELIMINARY VALIDATION REPORT

4 Data and refinement statistics (i)

Property	Value	Source
Space group	P 1 21 1	Depositor
Cell constants<br>a, b, c, α, β, γ	67.67Å 42.21Å 69.72Å<br>90.00° 98.91° 90.00°	Depositor
Resolution (Å)	12.61 – 1.50<br>12.61 – 1.50	Depositor<br>EDS
% Data completeness<br>(in resolution range)	100.0 (12.61-1.50)<br>100.0 (12.61-1.50)	Depositor<br>EDS
R_{merge}	(Not available)	Depositor
R_{sym}	(Not available)	Depositor
$< I/\sigma(I) >$ [1]	4.37 (at 1.50Å)	Xtriage
Refinement program	phenix.refine 1.14_3260, PHENIX 1.14_3260	Depositor
R, R_{free}	0.156 , 0.182<br>0.156 , 0.182	Depositor<br>DCC
R_{free} test set	3110 reflections (4.98%)	wwPDB-VP
Wilson B-factor (Å^2)	11.8	Xtriage
Anisotropy	0.214	Xtriage
Bulk solvent k_{sol}(e/Å^3), B_{sol}(Å^2)	0.32 , 45.3	EDS
L-test for twinning[2]	$< \|L\| > = 0.51$, $< L^2 > = 0.34$	Xtriage
Estimated twinning fraction	0.009 for l,-k,h	Xtriage
F_o,F_c correlation	0.97	EDS
Total number of atoms	4327	wwPDB-VP
Average B, all atoms (Å^2)	16.0	wwPDB-VP

Xtriage's analysis on translational NCS is as follows: *The largest off-origin peak in the Patterson function is 9.05% of the height of the origin peak. No significant pseudotranslation is detected.*

[1]Intensities estimated from amplitudes.

[2]Theoretical values of $< |L| >$, $< L^2 >$ for acentric reflections are 0.5, 0.333 respectively for untwinned datasets, and 0.375, 0.2 for perfectly twinned datasets.

PRELIMINARY VALIDATION REPORT

5 Model quality ⓘ

5.1 Standard geometry ⓘ

Bond lengths and bond angles in the following residue types are not validated in this section: NAG, MAN, CU

The Z score for a bond length (or angle) is the number of standard deviations the observed value is removed from the expected value. A bond length (or angle) with $|Z| > 5$ is considered an outlier worth inspection. RMSZ is the root-mean-square of all Z scores of the bond lengths (or angles).

Mol	Chain	Bond lengths		Bond angles	
		RMSZ	#$\|Z\|$ >5	RMSZ	#$\|Z\|$ >5
1	A	0.41	2/1803 (0.1%)	0.57	0/2462
1	B	0.41	0/1844	0.64	0/2515
All	All	0.41	2/3647 (0.1%)	0.61	0/4977

Chiral center outliers are detected by calculating the chiral volume of a chiral center and verifying if the center is modelled as a planar moiety or with the opposite hand.A planarity outlier is detected by checking planarity of atoms in a peptide group, atoms in a mainchain group or atoms of a sidechain that are expected to be planar.

Mol	Chain	#Chirality outliers	#Planarity outliers
1	A	0	2
1	B	0	2
All	All	0	4

All (2) bond length outliers are listed below:

Mol	Chain	Res	Type	Atoms	Z	Observed(Å)	Ideal(Å)
1	A	42[A]	SER	C-O	5.05	1.32	1.23
1	A	42[B]	SER	C-O	5.05	1.32	1.23

There are no bond angle outliers.

There are no chirality outliers.

All (4) planarity outliers are listed below:

Mol	Chain	Res	Type	Group
1	A	56[B]	GLN	Mainchain
1	A	96[B]	ASP	Mainchain
1	B	45[A]	THR	Mainchain
1	B	45[C]	THR	Mainchain

PRELIMINARY VALIDATION REPORT

5.2 Too-close contacts (i)

In the following table, the Non-H and H(model) columns list the number of non-hydrogen atoms and hydrogen atoms in the chain respectively. The H(added) column lists the number of hydrogen atoms added and optimized by MolProbity. The Clashes column lists the number of clashes within the asymmetric unit, whereas Symm-Clashes lists symmetry-related clashes.

Mol	Chain	Non-H	H(model)	H(added)	Clashes	Symm-Clashes
1	A	1729	0	1706	8	0
1	B	1752	0	1737	15	0
2	C	28	0	25	0	0
3	D	39	0	33	0	0
4	A	1	0	0	0	0
4	B	1	0	0	0	0
5	A	366	0	0	0	1
5	B	411	0	0	11	1
All	All	4327	0	3501	23	1

The all-atom clashscore is defined as the number of clashes found per 1000 atoms (including hydrogen atoms). The all-atom clashscore for this structure is 3.

All (23) close contacts within the same asymmetric unit are listed below, sorted by their clash magnitude.

Atom-1	Atom-2	Interatomic distance (Å)	Clash overlap (Å)
1:B:96[B]:ASP:OD1	5:B:456:HOH:O	1.78	1.00
1:B:93[A]:LYS:HE3	1:B:103[A]:GLY:O	1.80	0.82
1:B:30:GLU:OE2	5:B:643:HOH:O	2.01	0.77
1:B:102[B]:SER:O	5:B:536:HOH:O	2.06	0.73
1:B:25[B]:TYR:CE1	5:B:476:HOH:O	2.48	0.65
1:B:97[B]:ASN:OD1	5:B:538:HOH:O	2.13	0.65
1:B:25[B]:TYR:CD1	5:B:476:HOH:O	2.56	0.59
1:A:205:ILE:HA	1:A:210:VAL:CG2	2.32	0.58
1:B:45[C]:THR:HG22	5:B:553:HOH:O	2.06	0.55
1:A:9[B]:VAL:HG12	1:A:61:VAL:HG12	1.88	0.54
1:A:9[A]:VAL:HG13	1:A:55[A]:VAL:HG13	1.92	0.52
1:B:68[B]:MET:HG2	5:B:492:HOH:O	2.09	0.50
1:A:121:VAL:HB	1:A:126:ARG:HH12	1.78	0.48
1:B:9:VAL:HG21	1:B:53:ILE:HG21	1.96	0.47
1:B:210:VAL:HG22	5:B:607:HOH:O	2.16	0.46
1:A:161:ASN:HA	1:A:202:LYS:HE3	1.99	0.44
1:A:9[A]:VAL:CG1	1:A:55[A]:VAL:HG13	2.48	0.44
1:B:140:GLU:HG2	5:B:804:HOH:O	2.19	0.42
1:B:86:GLY:HA3	1:B:154:ILE:O	2.20	0.42

Continued on next page...

PRELIMINARY VALIDATION REPORT

Continued from previous page...

Atom-1	Atom-2	Interatomic distance (Å)	Clash overlap (Å)
1:A:86:GLY:HA3	1:A:154:ILE:O	2.20	0.42
1:A:207:TRP:HA	1:A:208:PRO:HA	1.96	0.41
1:B:28:PRO:HB3	1:B:168:TYR:CZ	2.56	0.40
1:B:96[B]:ASP:CG	5:B:456:HOH:O	2.43	0.40

All (1) symmetry-related close contacts are listed below. The label for Atom-2 includes the symmetry operator and encoded unit-cell translations to be applied.

Atom-1	Atom-2	Interatomic distance (Å)	Clash overlap (Å)
5:A:708:HOH:O	5:B:713:HOH:O[1_554]	2.11	0.09

5.3 Torsion angles (i)

5.3.1 Protein backbone (i)

In the following table, the Percentiles column shows the percent Ramachandran outliers of the chain as a percentile score with respect to all X-ray entries followed by that with respect to entries of similar resolution.

The Analysed column shows the number of residues for which the backbone conformation was analysed, and the total number of residues.

Mol	Chain	Analysed	Favoured	Allowed	Outliers	Percentiles	
1	A	238/223 (107%)	230 (97%)	7 (3%)	1 (0%)	34	13
1	B	245/223 (110%)	238 (97%)	6 (2%)	1 (0%)	34	13
All	All	483/446 (108%)	468 (97%)	13 (3%)	2 (0%)	29	13

All (2) Ramachandran outliers are listed below:

Mol	Chain	Res	Type
1	B	164	GLY
1	A	164	GLY

5.3.2 Protein sidechains (i)

In the following table, the Percentiles column shows the percent sidechain outliers of the chain as a percentile score with respect to all X-ray entries followed by that with respect to entries of similar resolution.

PRELIMINARY VALIDATION REPORT

The Analysed column shows the number of residues for which the sidechain conformation was analysed, and the total number of residues.

Mol	Chain	Analysed	Rotameric	Outliers	Percentiles	
1	A	195/178 (110%)	192 (98%)	3 (2%)	65	39
1	B	201/178 (113%)	200 (100%)	1 (0%)	88	78
All	All	396/356 (111%)	392 (99%)	4 (1%)	81	57

All (4) residues with a non-rotameric sidechain are listed below:

Mol	Chain	Res	Type
1	A	4	PHE
1	A	42[A]	SER
1	A	42[B]	SER
1	B	4	PHE

Sometimes sidechains can be flipped to improve hydrogen bonding and reduce clashes. There are no such sidechains identified.

5.3.3 RNA (i)

There are no RNA molecules in this entry.

5.4 Non-standard residues in protein, DNA, RNA chains (i)

There are no non-standard protein/DNA/RNA residues in this entry.

5.5 Carbohydrates (i)

5 monosaccharides are modelled in this entry.

In the following table, the Counts columns list the number of bonds (or angles) for which Mogul statistics could be retrieved, the number of bonds (or angles) that are observed in the model and the number of bonds (or angles) that are defined in the Chemical Component Dictionary. The Link column lists molecule types, if any, to which the group is linked. The Z score for a bond length (or angle) is the number of standard deviations the observed value is removed from the expected value. A bond length (or angle) with $|Z| > 2$ is considered an outlier worth inspection. RMSZ is the root-mean-square of all Z scores of the bond lengths (or angles).

Mol	Type	Chain	Res	Link	Bond lengths			Bond angles		
					Counts	RMSZ	$\#\|Z\| > 2$	Counts	RMSZ	$\#\|Z\| > 2$
2	NAG	C	1	1,2	14,14,15	0.21	0	17,19,21	0.44	0

PRELIMINARY VALIDATION REPORT

Mol	Type	Chain	Res	Link	Bond lengths			Bond angles		
					Counts	RMSZ	#|Z| > 2	Counts	RMSZ	#|Z| > 2
2	NAG	C	2	2	14,14,15	2.04	4 (28%)	17,19,21	1.94	6 (35%)
3	NAG	D	1	1,3	14,14,15	0.32	0	17,19,21	0.41	0
3	NAG	D	2	3	14,14,15	1.26	1 (7%)	17,19,21	1.65	4 (23%)
3	MAN	D	3	3	11,11,12	1.05	2 (18%)	15,15,17	1.13	2 (13%)

In the following table, the Chirals column lists the number of chiral outliers, the number of chiral centers analysed, the number of these observed in the model and the number defined in the Chemical Component Dictionary. Similar counts are reported in the Torsion and Rings columns. '-' means no outliers of that kind were identified.

Mol	Type	Chain	Res	Link	Chirals	Torsions	Rings
2	NAG	C	1	1,2	-	0/6/23/26	0/1/1/1
2	NAG	C	2	2	-	0/6/23/26	0/1/1/1
3	NAG	D	1	1,3	-	0/6/23/26	0/1/1/1
3	NAG	D	2	3	-	4/6/23/26	0/1/1/1
3	MAN	D	3	3	-	2/2/19/22	1/1/1/1

All (7) bond length outliers are listed below:

Mol	Chain	Res	Type	Atoms	Z	Observed(Å)	Ideal(Å)
2	C	2	NAG	O5-C1	-4.87	1.35	1.43
3	D	2	NAG	O5-C1	-3.25	1.38	1.43
2	C	2	NAG	C1-C2	-3.05	1.47	1.52
2	C	2	NAG	O7-C7	-2.74	1.17	1.23
2	C	2	NAG	C2-N2	-2.41	1.42	1.46
3	D	3	MAN	O5-C5	2.32	1.48	1.43
3	D	3	MAN	C1-C2	2.18	1.57	1.52

All (12) bond angle outliers are listed below:

Mol	Chain	Res	Type	Atoms	Z	Observed(°)	Ideal(°)
2	C	2	NAG	C8-C7-N2	3.88	122.67	116.10
3	D	2	NAG	C6-C5-C4	-3.58	104.63	113.00
2	C	2	NAG	O7-C7-C8	-3.41	115.73	122.06
2	C	2	NAG	C2-N2-C7	3.36	127.69	122.90
3	D	2	NAG	O6-C6-C5	-3.31	99.92	111.29
2	C	2	NAG	C4-C3-C2	3.01	115.43	111.02
3	D	3	MAN	C1-O5-C5	2.92	116.14	112.19
3	D	2	NAG	O3-C3-C2	-2.58	104.12	109.47
2	C	2	NAG	O4-C4-C5	-2.24	103.74	109.30
2	C	2	NAG	C1-C2-N2	2.13	114.12	110.49

Continued on next page...

PRELIMINARY VALIDATION REPORT

Continued from previous page...

Mol	Chain	Res	Type	Atoms	Z	Observed(°)	Ideal(°)
3	D	2	NAG	C1-O5-C5	2.05	114.97	112.19
3	D	3	MAN	O2-C2-C3	-2.02	106.08	110.14

There are no chirality outliers.

All (6) torsion outliers are listed below:

Mol	Chain	Res	Type	Atoms
3	D	2	NAG	C4-C5-C6-O6
3	D	2	NAG	C8-C7-N2-C2
3	D	2	NAG	O7-C7-N2-C2
3	D	2	NAG	O5-C5-C6-O6
3	D	3	MAN	O5-C5-C6-O6
3	D	3	MAN	C4-C5-C6-O6

All (1) ring outliers are listed below:

Mol	Chain	Res	Type	Atoms
3	D	3	MAN	C1-C2-C3-C4-C5-O5

No monomer is involved in short contacts.

The following is a two-dimensional graphical depiction of Mogul quality analysis of bond lengths, bond angles, torsion angles, and ring geometry for oligosaccharide.

PRELIMINARY VALIDATION REPORT

Oligosaccharide Chain C

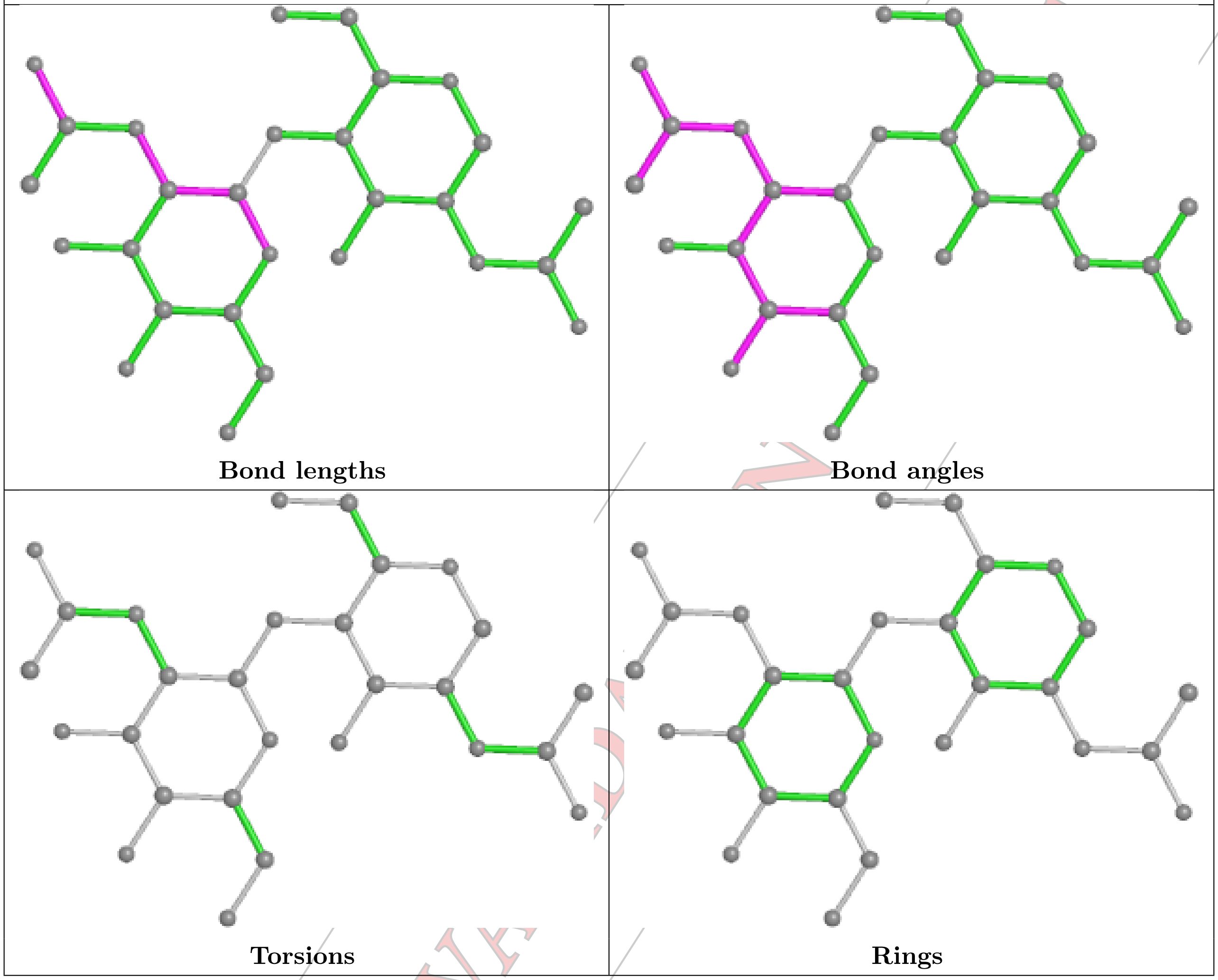

PRELIMINARY VALIDATION

Oligosaccharide Chain D

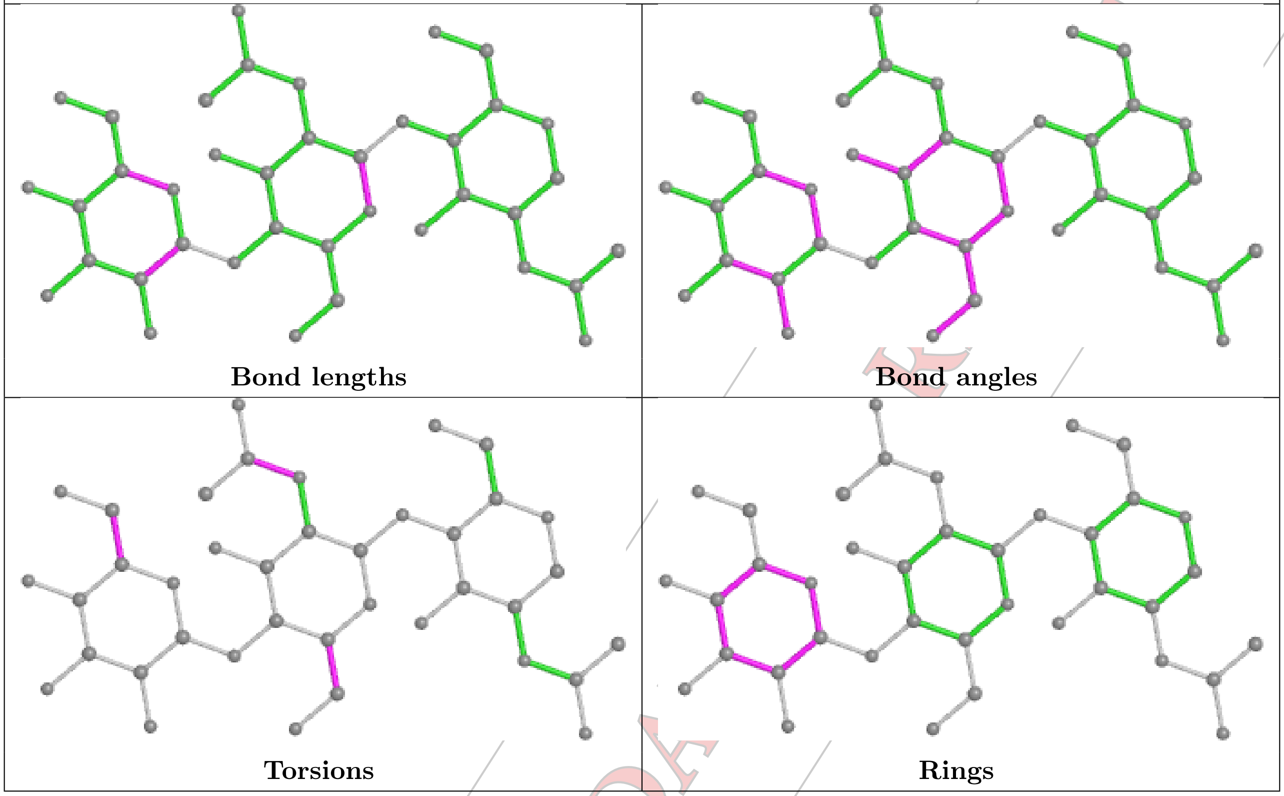

5.6 Ligand geometry (i)

Of 2 ligands modelled in this entry, 2 are monoatomic - leaving 0 for Mogul analysis.

There are no bond length outliers.

There are no bond angle outliers.

There are no chirality outliers.

There are no torsion outliers.

There are no ring outliers.

No monomer is involved in short contacts.

5.7 Other polymers (i)

There are no such residues in this entry.

5.8 Polymer linkage issues (i)

There are no chain breaks in this entry.

PRELIMINARY VALIDATION REPORT

6 Fit of model and data (i)

6.1 Protein, DNA and RNA chains (i)

In the following table, the column labelled '#RSRZ> 2' contains the number (and percentage) of RSRZ outliers, followed by percent RSRZ outliers for the chain as percentile scores relative to all X-ray entries and entries of similar resolution. The OWAB column contains the minimum, median, 95th percentile and maximum values of the occupancy-weighted average B-factor per residue. The column labelled 'Q< 0.9' lists the number of (and percentage) of residues with an average occupancy less than 0.9.

Mol	Chain	Analysed	<RSRZ>	#RSRZ>2			OWAB(Å^2)	Q<0.9
1	A	223/223 (100%)	-0.15	6 (2%)	54	59	7, 14, 27, 44	0
1	B	223/223 (100%)	-0.45	1 (0%)	92	94	6, 11, 20, 38	1 (0%)
All	All	446/446 (100%)	-0.30	7 (1%)	72	77	6, 12, 24, 44	1 (0%)

All (7) RSRZ outliers are listed below:

Mol	Chain	Res	Type	RSRZ
1	B	207	TRP	4.6
1	A	119	SER	3.1
1	A	118	SER	3.0
1	A	209	PRO	2.9
1	A	211	THR	2.6
1	A	223	CYS	2.3
1	A	212	SER	2.1

6.2 Non-standard residues in protein, DNA, RNA chains (i)

There are no non-standard protein/DNA/RNA residues in this entry.

6.3 Carbohydrates (i)

In the following table, the Atoms column lists the number of modelled atoms in the group and the number defined in the chemical component dictionary. The B-factors column lists the minimum, median, 95th percentile and maximum values of B factors of atoms in the group. The column labelled 'Q< 0.9' lists the number of atoms with occupancy less than 0.9.

Mol	Type	Chain	Res	Atoms	RSCC	RSR	B-factors(Å^2)	Q<0.9
3	MAN	D	3	11/?	0.72	0.25	48,51,57,64	0
2	NAG	C	2	14/?	0.85	0.17	23,34,42,49	0

Continued on next page...

PRELIMINARY VALIDATION REPORT

Continued from previous page...

Mol	Type	Chain	Res	Atoms	RSCC	RSR	B-factors(Å^2)	Q<0.9
3	NAG	D	2	14/?	0.90	0.15	18,30,35,42	0
3	NAG	D	1	14/?	0.91	0.12	13,18,45,50	0
2	NAG	C	1	14/?	0.92	0.10	14,21,31,35	0

The following is a graphical depiction of the model fit to experimental electron density for oligosaccharide. Each fit is shown from different orientation to approximate a three-dimensional view.

Electron density around Chain C:

2mF$_o$-DF$_c$ (at 0.7 rmsd) in gray
mF$_o$-DF$_c$ (at 3 rmsd) in purple (negative)
and green (positive)

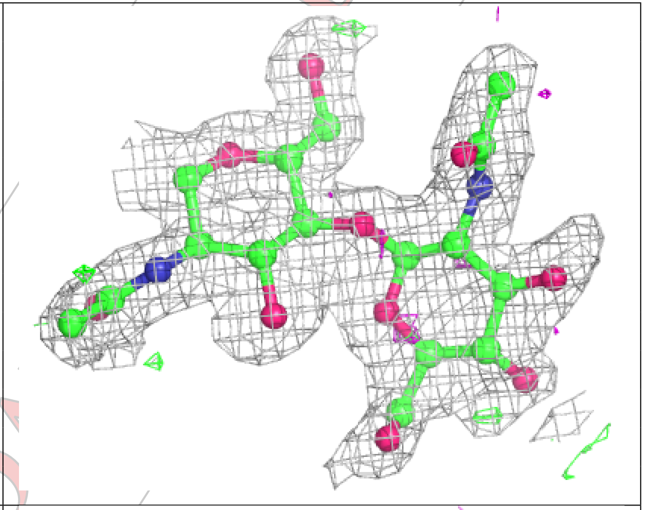

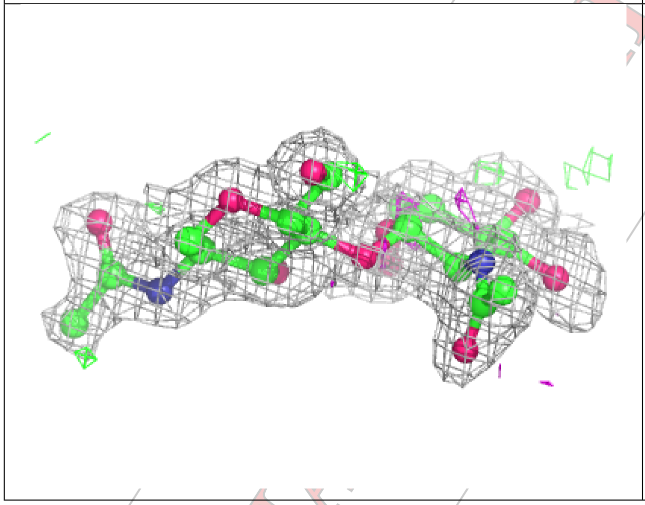

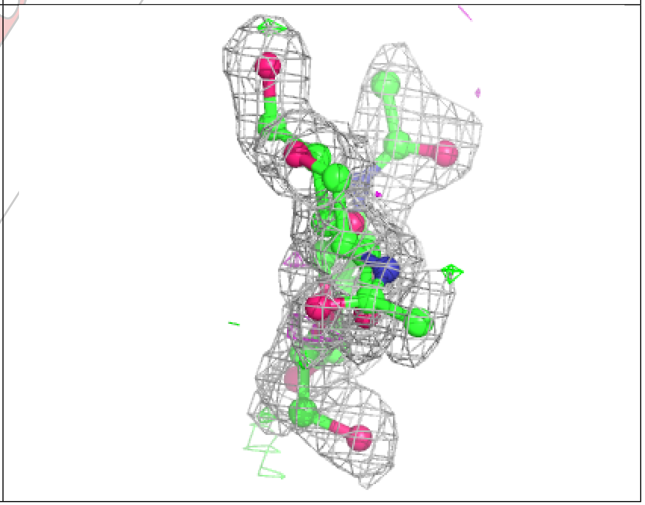

PRELIMINARY REPORT

Electron density around Chain D:

2mF$_o$-DF$_c$ (at 0.7 rmsd) in gray
mF$_o$-DF$_c$ (at 3 rmsd) in purple (negative)
and green (positive)

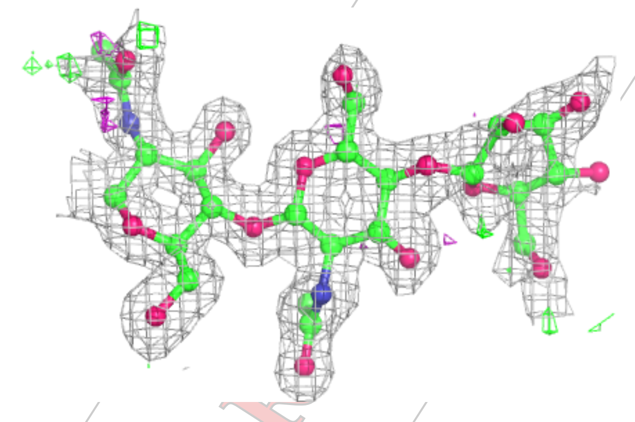

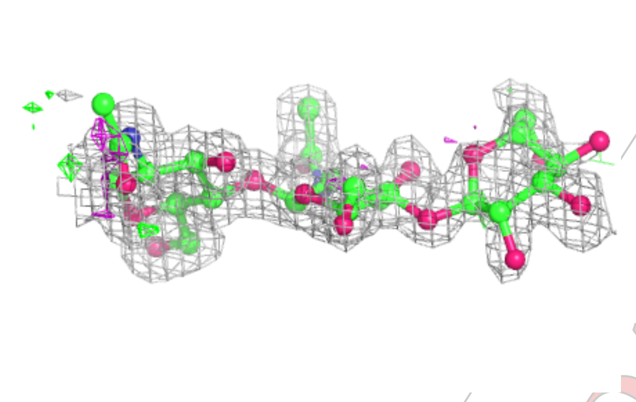

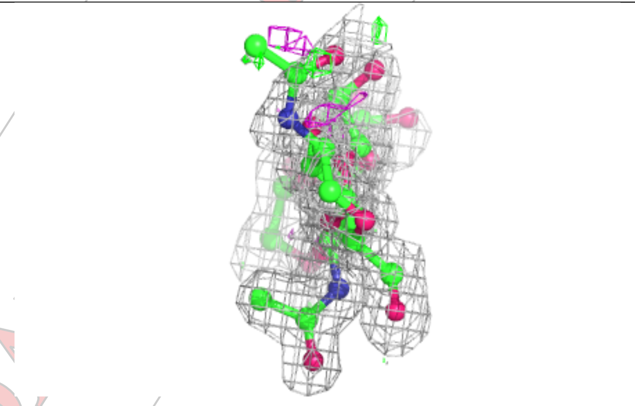

6.4 Ligands (i)

In the following table, the Atoms column lists the number of modelled atoms in the group and the number defined in the chemical component dictionary. The B-factors column lists the minimum, median, 95th percentile and maximum values of B factors of atoms in the group. The column labelled 'Q< 0.9' lists the number of atoms with occupancy less than 0.9.

Mol	Type	Chain	Res	Atoms	RSCC	RSR	B-factors(Å^2)	Q<0.9
4	CU	A	301	1/?	1.00	0.03	12,12,12,12	0
4	CU	B	301	1/?	1.00	0.02	11,11,11,11	0

6.5 Other polymers (i)

There are no such residues in this entry.

PRELIMINARY VALID
